# Supplementary figures and images for: Linked surveillance and genetic data uncovers programmatically relevant geographic scale of Guinea worm transmission in Chad
Source: PLoS Negl Trop Dis. 2021 Jul 26;15(7):e0009609. doi: 10.1371/journal.pntd.0009609 (PMC8341693; doi:10.1371/journal.pntd.0009609)

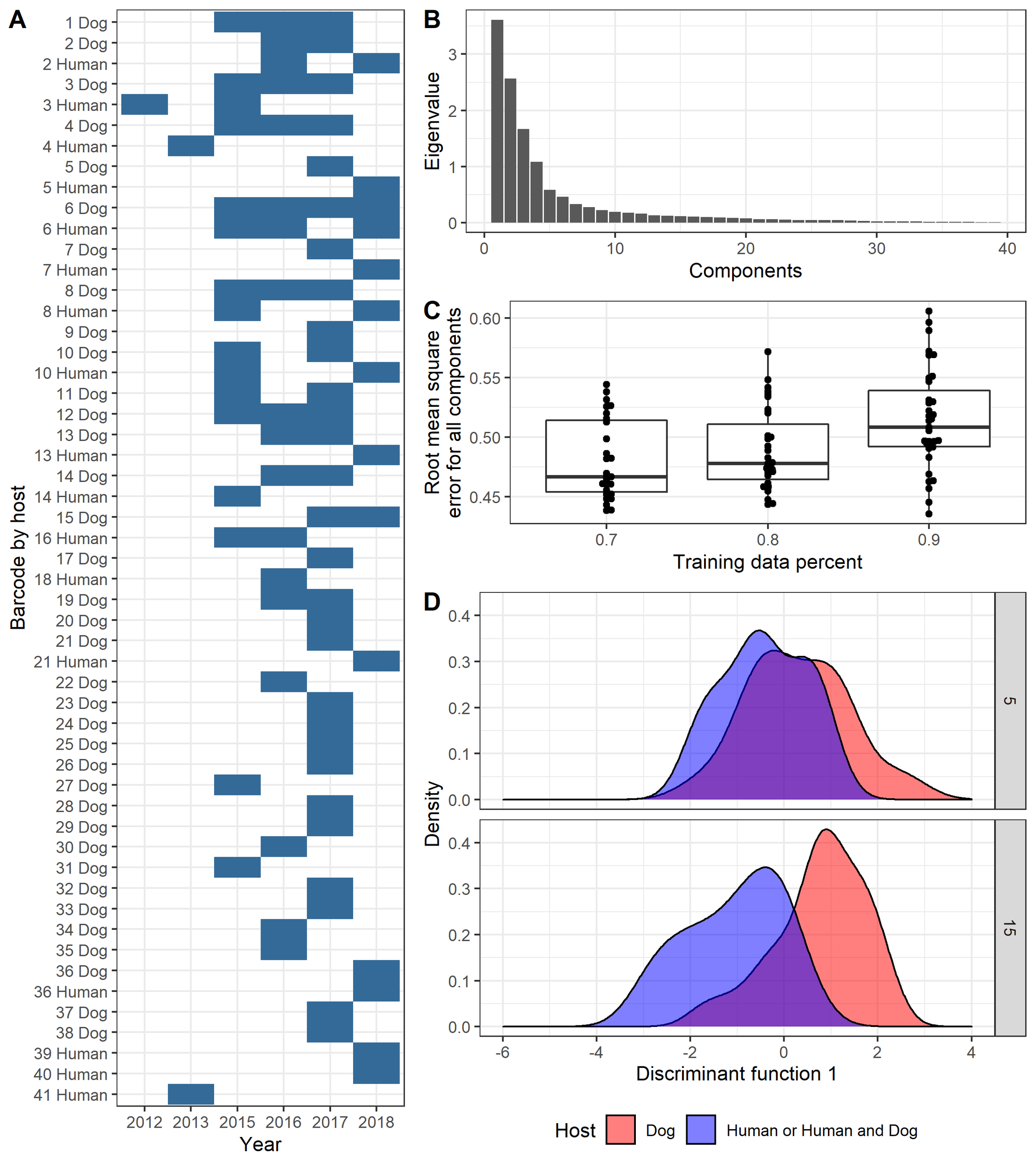

Supplement: S1 Fig — A. The presence of different barcodes per host by year. A blue-filled cell next to each barcode is indicative that the barcode was represented at least once in the respective year. Refer to S2 Table for sample counts of each barcode by species. B. DAPC eigenvalues for principal components with all 41 unique barcodes. A drop in variance is observed from 4 to 5 components, but cross-validation suggests 15 components provide the highest classification success. C. Root mean square error (RMSE) ranges for components with different training set percentages. While using 90% of the data as the training set produces the highest RMSE distribution, it obtains the lowest RMSE when compared to the 70% or 80% training set scenarios. D. DAPC analyses by number of components. Barcodes found in samples from humans only were grouped with barcodes found in samples from humans and dogs to create more even groups with barcodes found only in samples from dogs (humans only = 5 barcodes, both = 12, dogs only = 24). (TIF) [file pntd.0009609.s004.tif]

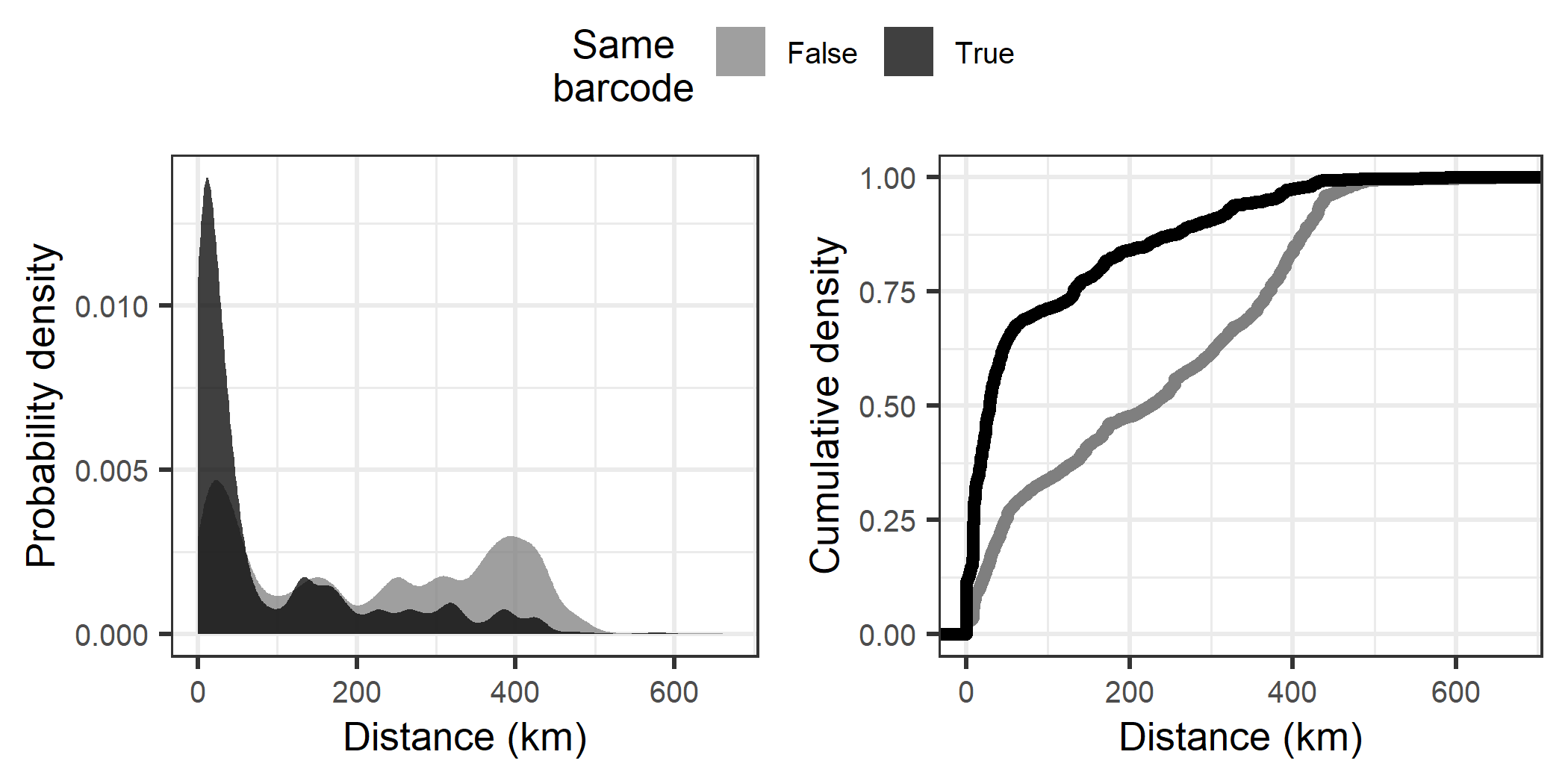

Supplement: S2 Fig — Number of pairwise comparisons for identical barcodes = 13,967, for non-identical barcodes = 76,558. (TIF) [file pntd.0009609.s005.tif]

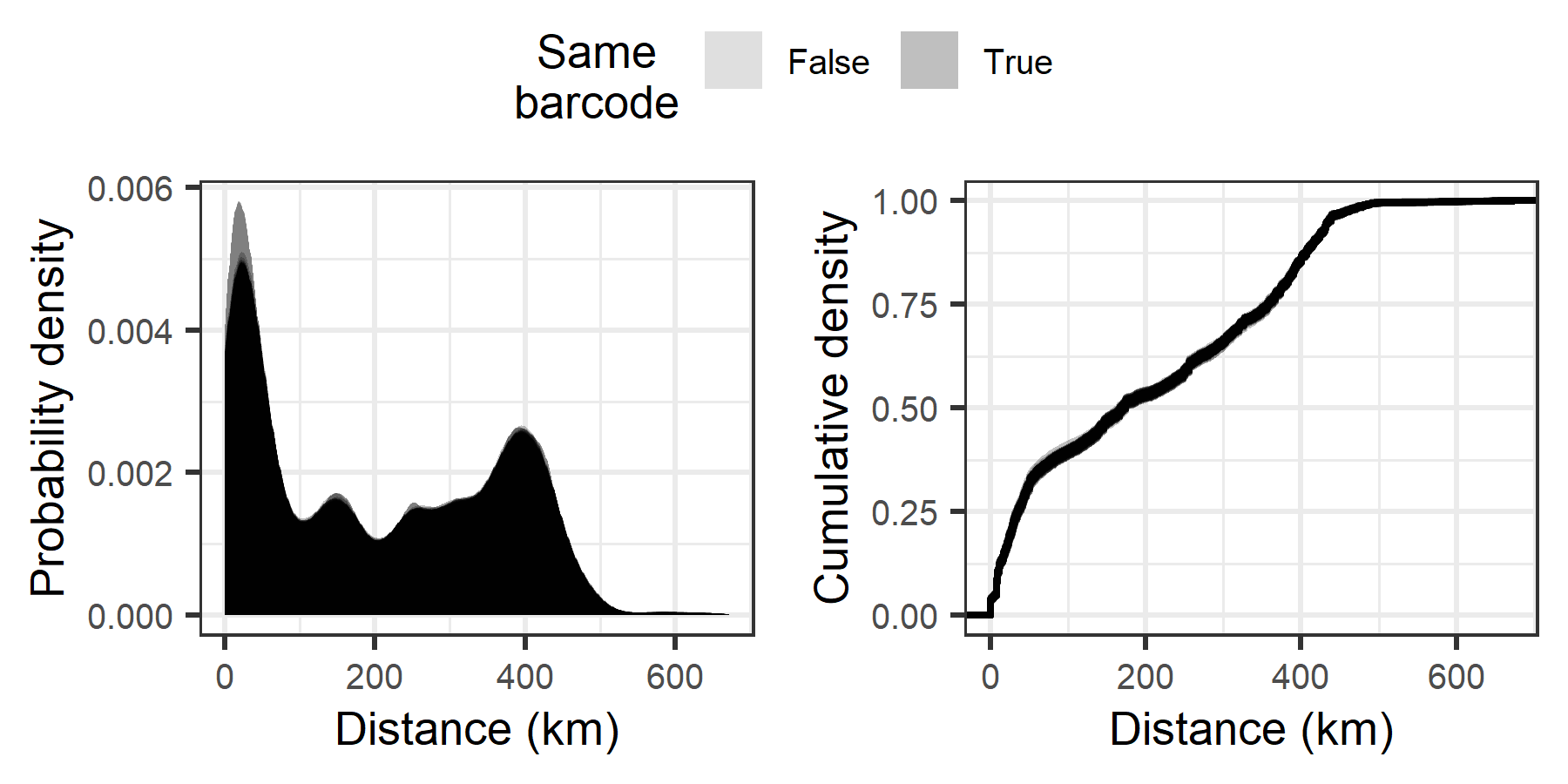

Supplement: S3 Fig — The x-axis is the permuted distance between pairs of worms and the y-axis represents the density for population genetic similarity scores for all worms (n = 426). Each line represents worm pair distance permutation (n = 100) for the population. The lines for identical and non-identical barcodes are consistent between permutations and overlap. (TIF) [file pntd.0009609.s006.tif]

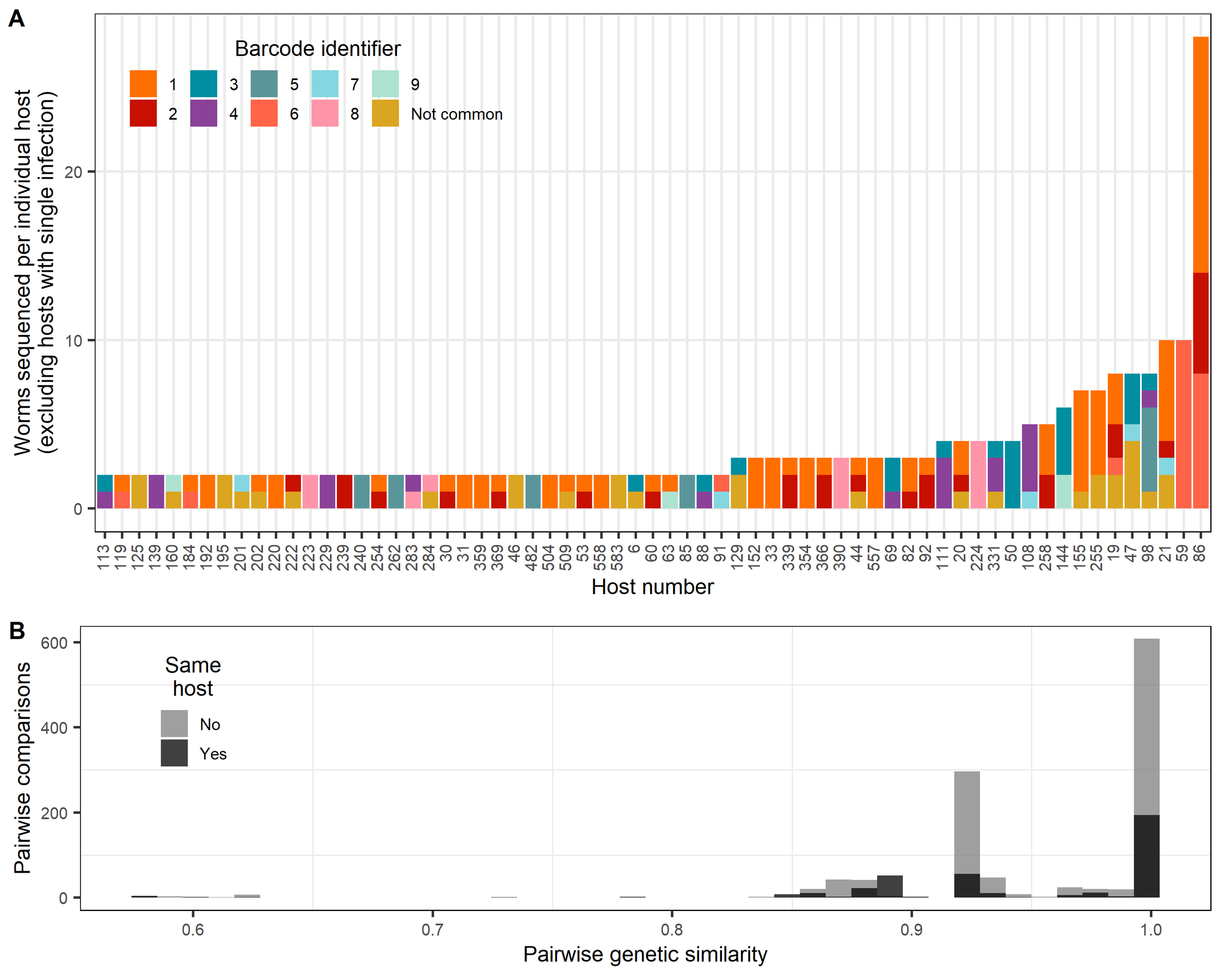

Supplement: S4 Fig — A. Each multi-infected host with the number of worms pertaining to each barcode set. “Not common” refers to barcodes found in less than 10 samples in the population for visual clarity. B. Distributions of genetic similarity between worms with the same reported GPS coordinates, colored by whether the pair is obtained from the same or different hosts. (TIF) [file pntd.0009609.s007.tif]

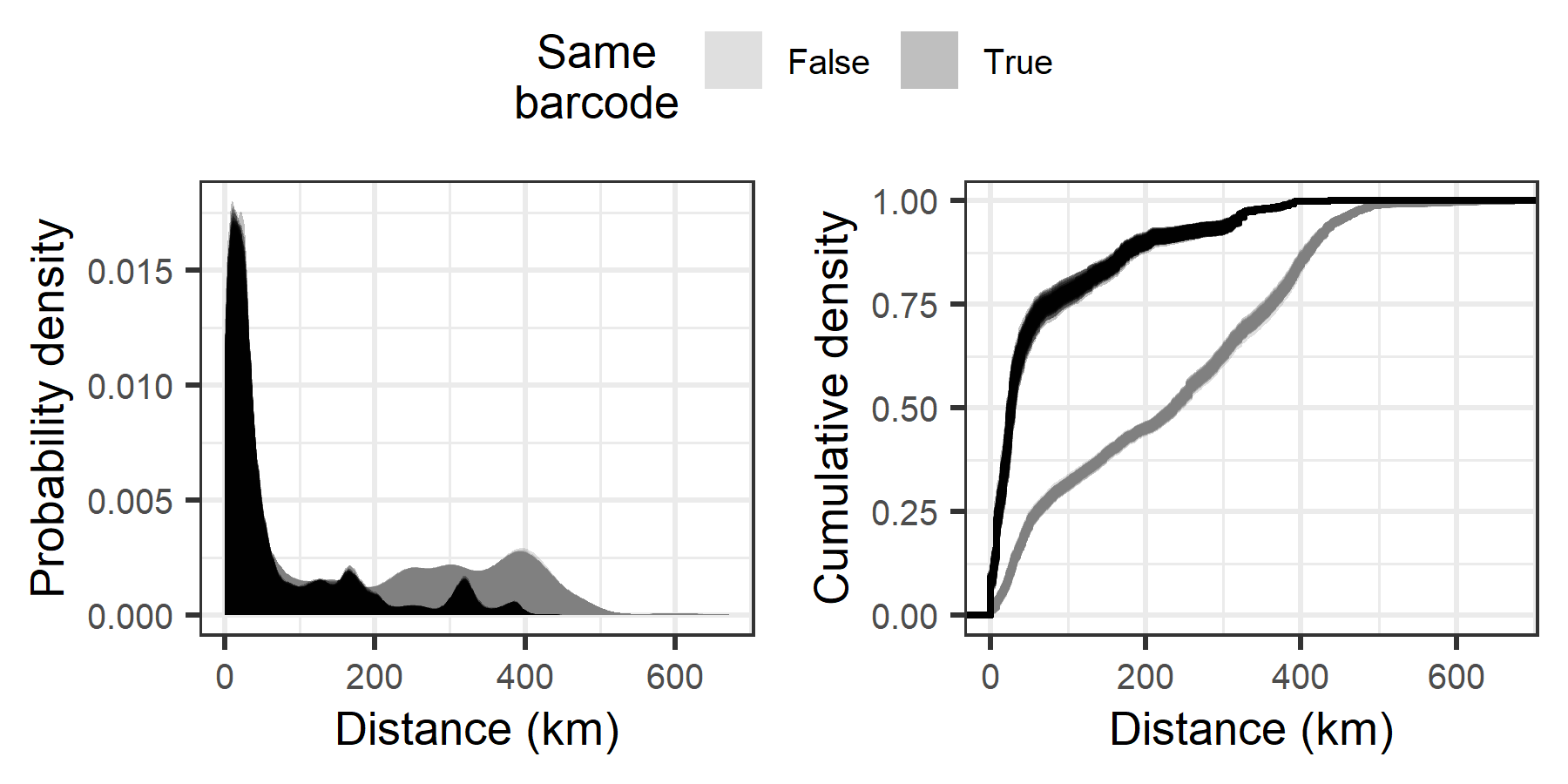

Supplement: S5 Fig — The x-axis is the distance between pairs of worms and the y-axis represents the density for population genetic similarity scores with one worm per host (n = 282). Each line represents a bootstrap (n = 100) of a single worm per host. (TIF) [file pntd.0009609.s008.tif]

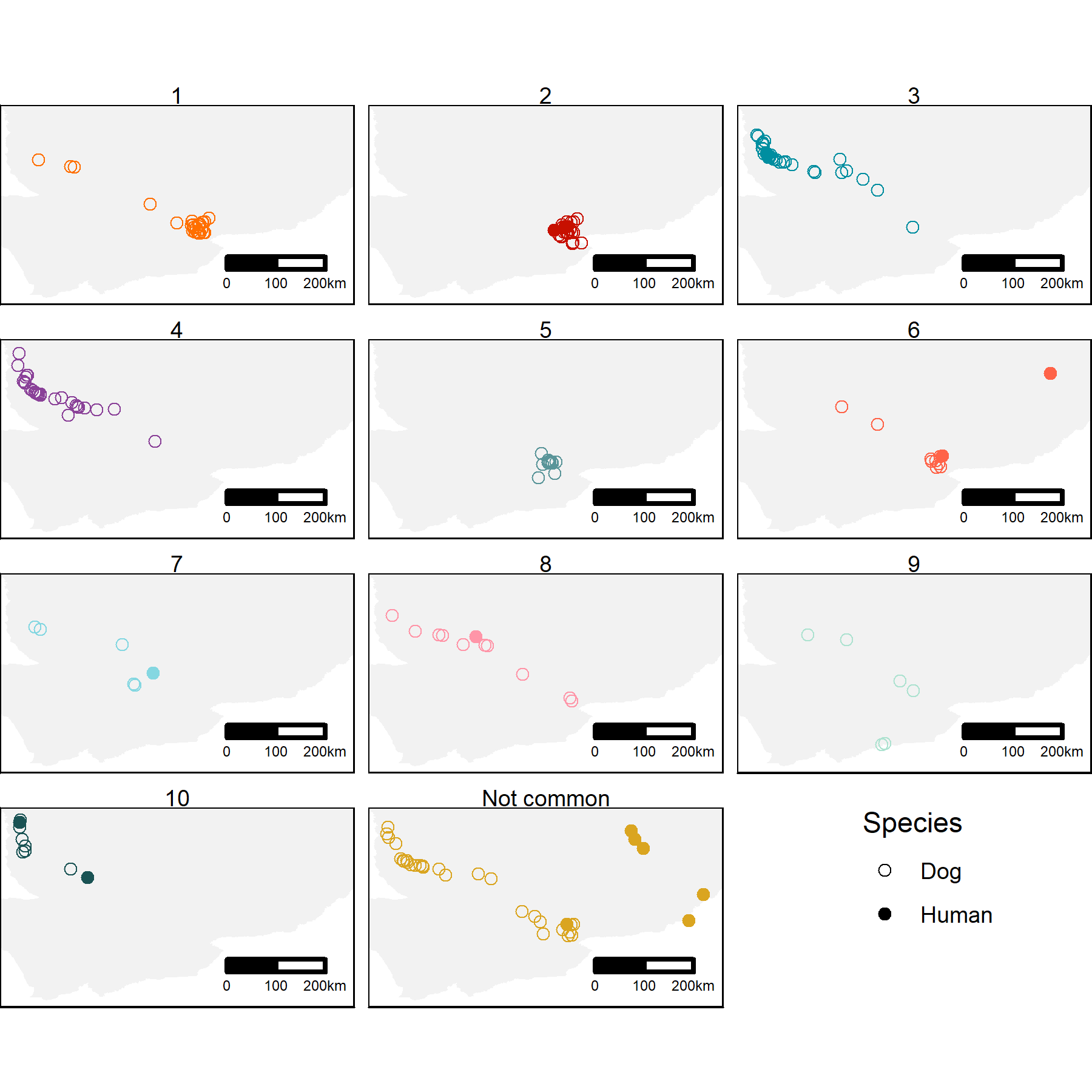

Supplement: S6 Fig — Samples are colored by their barcode identifier, and shapes represent the host species. Barcodes 3, 4, and 10 have similar spatial distributions. Barcodes 2 and 5 have similar spatial distributions and are highly focal. Some barcodes span a very large geographic area, which suggests they are ancestral sequences that have diffused over time or are transmitting due to human behaviors. (TIF) [file pntd.0009609.s009.tif]

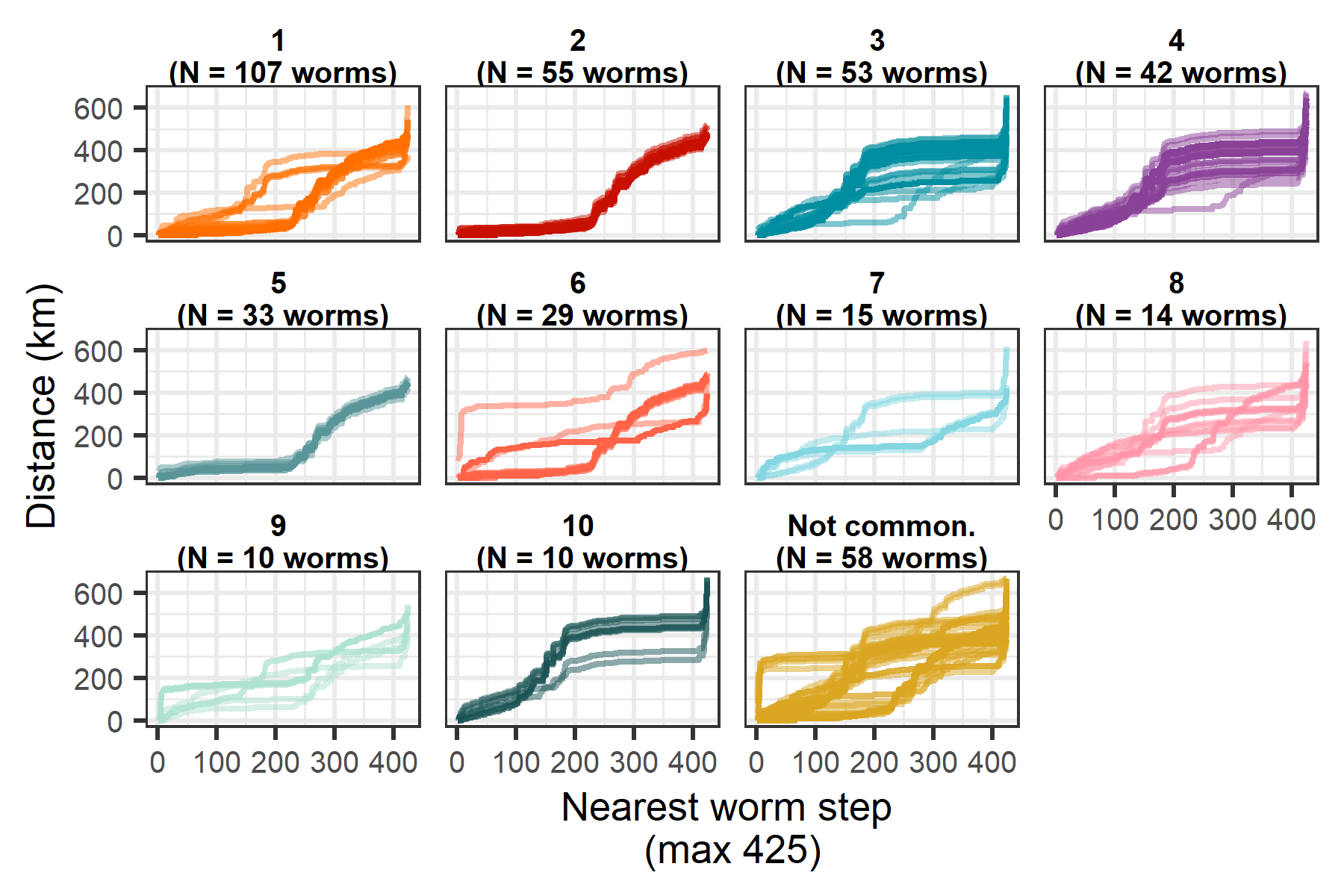

Supplement: S7 Fig — The geographic distance between worms relative to a single worm were organized in ascending order. The x-axis is the cumulative worm count by distance and the y-axis is the cumulative distance from each index worm. Spans of a flattened curve are indicative of geographic stretches that do not contain any samples and support the lack of barcode diversity observed in certain geographic distances in Fig 3. (TIF) [file pntd.0009609.s010.tif]

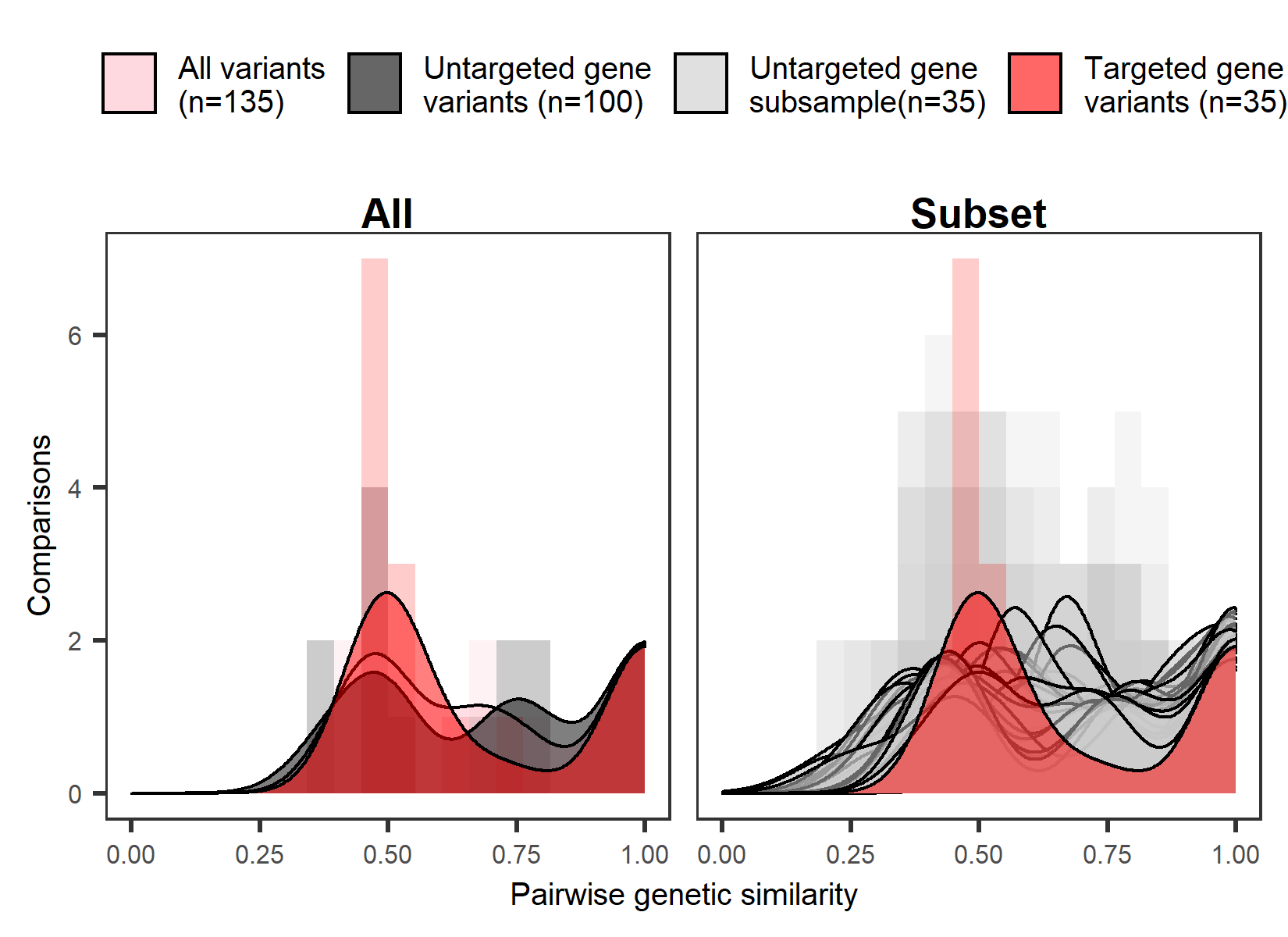

Supplement: S8 Fig — The x-axis is the measured genetic similarity for variants within genes targeted by the amplification protocol (n = 35) and extending to the rest of the mitochondrial genome (n = 100), and y-axis is the number of pairwise comparisons (19*19 = 361). Filled regions show the smoothed density estimates for histograms. (TIF) [file pntd.0009609.s011.tif]

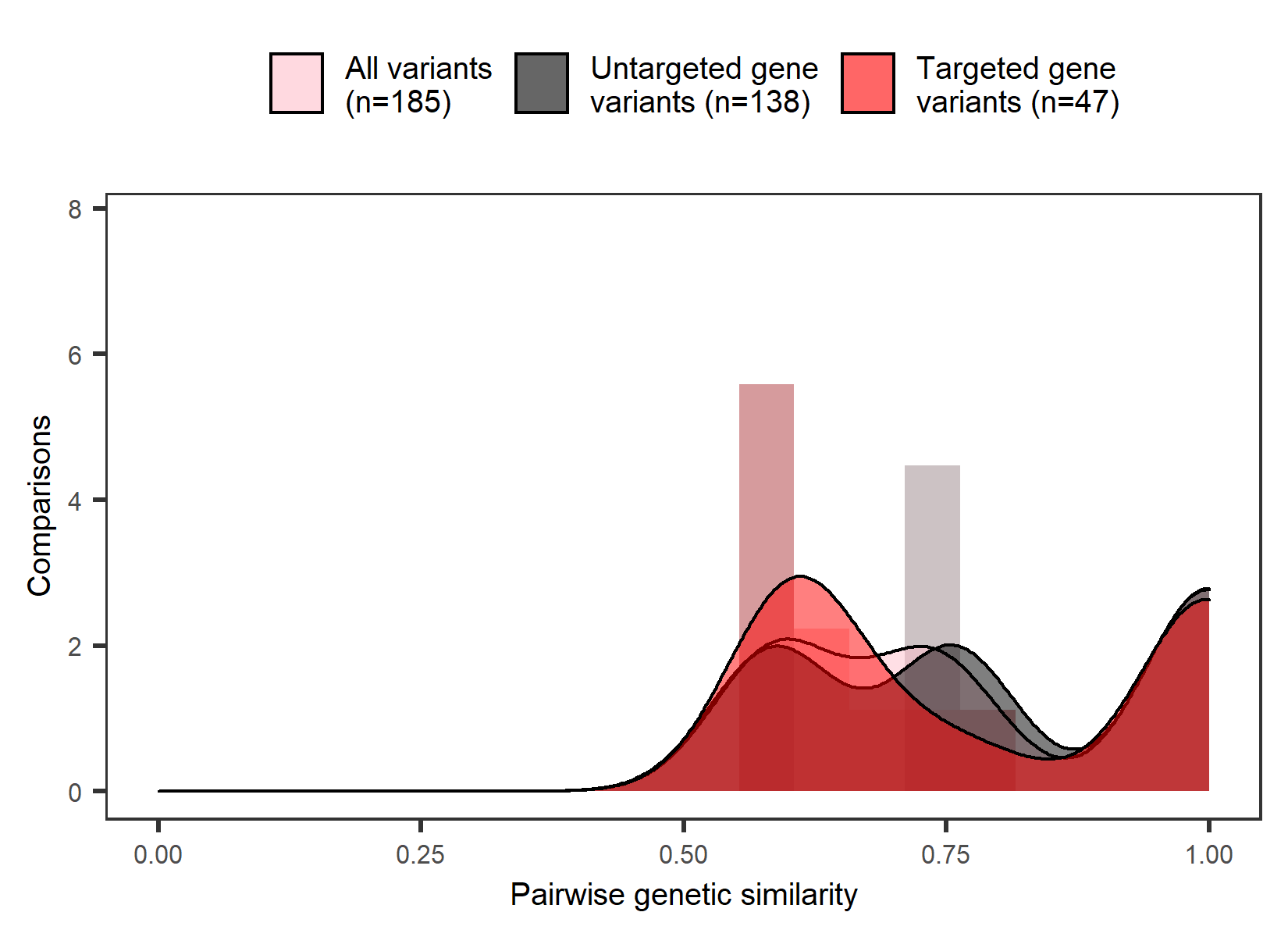

Supplement: S9 Fig — The x-axis is the measured genetic similarity for variants within genes targeted by the amplification protocol (n = 47) and extending to the rest of the mitochondrial genome (n = 138), and y-axis is the number of pairwise comparisons (19*19 = 361). Filled regions show the smoothed density estimates for histograms. (TIF) [file pntd.0009609.s012.tif]
