# Supplementary material for: Linked surveillance and genetic data uncovers programmatically relevant geographic scale of Guinea worm transmission in Chad
Source: PLoS Negl Trop Dis. 2021 Jul 26;15(7):e0009609. doi: 10.1371/journal.pntd.0009609 (PMC8341693; doi:10.1371/journal.pntd.0009609)
Supplement: Attachment — Submitted filename: GuineaWormPhylodynamics_ResponseToReviewers_v2.pdf [file pntd.0009609.s016.pdf]

We are grateful to the editors and reviewers for their thoughtful comments that have significantly improved the manuscript.

We have provided a point-by-point response to the comments below. Changes in the revised manuscript are highlighted in blue. To briefly summarize how we addressed recurring comments from reviewers:

1) We added details describing how true variant singletons were distinguished from noise. True singleton variants were retained in the molecular barcode generated from all variants identified in the sampled population.
2) We removed the supplemental phylogenetic analysis from our study following a recommendation to use a better population analysis method for our goal to distinguish barcodes by host species.
3) For the complete mitochondrial genome analyses in an independent Guinea worm population, we added the motivation for exploring more variants across the mitochondrial genome. Additionally, we added supporting details that “within loci” covers any variants that fall within the genomic ranges of the three CO3, cytB, and ND3-5 loci targeted in the spatial analyses. “Outside loci” covers any variants found in coding and non-coding regions of the genome outside of the ranges for the three loci.
4) A “Data Availability” section with the accession numbers for sequencing data has been provided. In-country partners are the owners of the epidemiological data and cannot be shared without their approval. We have added this statement along with the contact information for other researchers interested in getting data access to this section.

From the editor:
The reviewers emphasized a few points that I also noticed and that need to be addressed. First among these is the point about “singletons” and how they were treated. I found this very hard to follow but understanding what you mean is important for comprehension of later analyses. A simple, brief, explanation is likely possible and I hope to see that in a revised version.

Thank you for this important comment. We have made changes to the manuscript to improve the clarity of the description of singletons. Specifically, singleton variants identified at positions were checked for randomness across samples. Two samples that had more than 50% of the singleton variants in two different genes were considered sequencing and alignment artifacts and were removed from the study. Six singletons that were found in different samples across the three mitochondrial loci amplified in the study were retained as true variants.

For the complete mitochondrial genome analyses, we perform the same analyses with singleton variants included and singleton variants excluded due to the low number of samples (19). We checked the genetic pairwise differences between worms was not an artifact of single variants due to the large number of variants (>100) relative to the population size. The robust changes in the shape of the pairwise genetic similarity distribution with the inclusion or exclusion of singleton variants suggests that the distribution of relatedness is not an artifact of including more, lower frequency variants.

The mitochondrial genome in animals is not subject to recombination: it is effectively a single locus. Different parts of the mt genome may accumulate mutations at different rates, but all should tell the same story, subject to the range of error. So, I was confused at first about use of the word “locus” and the idea that some variants were apparently outside loci and others inside. It became clear that you were referring to a protein-coding gene as a “locus” and therefore regarded variants in non-coding regions as “outside” the locus. I found this usage confusing. It would be better to replace “locus” with “protein-coding gene” or similar.

Thank you for this observation. Locus is used to describe the genomic ranges of the four genes, CO3, cytB, and ND3-5 that were targeted for amplification. Variants identified ‘outside the loci’ are any variants that fall in any portion of the genome that does not overlap the genomic ranges of the amplified genes. We have updated the manuscript to provide this definition for within and outside the loci.

Other small points.
Line 7. I don’t think that “dracunculiasis” should start with an upper-case letter.
Correct capitalization has been updated in the manuscript.

Line 10. Hyphenate “decade-long”.
The manuscript has been updated with this edit.

Line 79. “Alleles”? I would have called them “variants”, as elsewhere.

We have updated the manuscript to be consistent with variant terminology.

Line 232."…30 human hosts…"
The manuscript has been updated to include "worms from [species] host"

Line 233. "Of" at the end of the line seems unnecessary.
Additional word removed from the manuscript.

Line 266. What is meant by "Only four barcodes from this set (7, 21, 40 and 41) were observed as a singular event." Maybe "only four barcodes were each found in a single worm"? In places, the language is over-complicated: this is one example.
This section of the manuscript was rewritten and condensed for clarity. The counts of individual barcodes and their respective host(s) are referenced in S1 Table. We have also attempted to address other instances of over-complicated language throughout the manuscript.

Line 423. Something is wrong here. The word "between" should maybe be deleted.
This sentence has been rewritten.

Caption to Fig. 2 has a couple of typos.
Typos have been corrected in all manuscript figures.

Reviewer's Responses to Questions
Key Review Criteria Required for Acceptance?
As you describe the new analyses required for acceptance, please consider the following:
Methods
-Are the objectives of the study clearly articulated with a clear testable hypothesis stated?
-Is the study design appropriate to address the stated objectives?
-Is the population clearly described and appropriate for the hypothesis being tested?
-Is the sample size sufficient to ensure adequate power to address the hypothesis being tested?
-Were correct statistical analysis used to support conclusions?
-Are there concerns about ethical or regulatory requirements being met?

Reviewer #1: The objectives of the study are articulated; the study design is appropriate; the population is clearly described with a sample size that is excellent for a population genetic study.

(1) Section 0.2.1 The description of how singletons were handled was a bit confusing. If I understand correctly, two worms both had "inflated" singletons (how many singletons count as inflated?), which suggests sequencing errors, and were excluded. For the remaining worms, six sites contained singletons and were removed from downstream analyses as they are not informative about relatedness between individual worms.
Thank you for bringing up this point on clarity. We have updated this section to better describe how variants were identified, criteria for determining which variants are true signals versus sequencing noise, and retaining the true variants to inform population diversity.

(2) Section 0.2.2 The use of the term “untargeted” can be removed as it's confusing—the entire mitochondrial genome was used, not just the portions that were not amplified and sequenced, so just saying “complete mitochondrial genome” is sufficient. Were optical duplicate reads and reads that mapped to more than one location in the genome removed in the analysis? If not, then this would need to be done; if it is part of the pipeline, please state this explicitly.

Thank you for pointing out the confusing terminology. We intended to highlight the difference between the targeted amplicon method of the three loci and the shotgun NGS approach from the downloaded samples from Durrant et al. 2020. We have updated the title to “complete mitochondria genome” and clarified the shotgun portion in the methods for this section. Only deduplicated, uniquely mapped reads were used in this analysis and we have added the deduplicated and unique mapping specification to the pipeline description.

(3) Section 0.3 In the phylogenetic and population genetic research communities, UPGMA has been regarded for decades as a poor estimator of phylogenetic relatedness. Other methods are expected to be more accurate if the input data are sufficiently informative (maximum likelihood, Bayesian approaches, or even neighbor joining). For their phylogenetic analysis, the authors assume the simplest model of sequence evolution. This is an assumption that needs to be tested prior to running the analysis. There is no attempt to measure statistical support for the tree; in a maximum likelihood framework, bootstrapping can provide as a commonly used metric for statistical support, while in a Bayesian framework, posterior probabilities indicate the strength of support for a particular relationship. Finally, changes over time cannot be explored on the basis of this tree because the tree is not rooted, which is an essential component to directional analysis. Phylogenetic methods are used viral epidemiology because viruses evolve very quickly within a single host. This is not true for parasites. I suspect that a phylogenetic tree built using solid phylogenetic methods would end up being a polytomy (i.e., no strong support for relationships between haplotypes). As an alternative to a phylogenetic approach, I might recommend instead applying population genetic methods that examine differences/changes in allele frequencies (i.e., the proportion of haplotypes in a given year). For example, discriminant analysis of principal components (DAPC) has easy-to-follow tutorials available online (Jombart et al. 2010). This could also be applied to produce a lovely visual comparison of how human vs dog haplotypes cannot be discriminated. That said, my intent here is not to push one particular method, but rather, to encourage the authors to increase the sophistication of whatever method they do use so that their conclusions are robust and well-supported statistically.

Thank you for this very thoughtful comment pointing out the limitations of UPGMA. Sequenced samples predominantly spanned three years with a few exceptions. This is relatively short compared to the 10-14 month life cycle of a worm from infection to emergence and the low mutation rate of the mitochondrial genome. We first tried UPGMA given that it’s a simple model of evolution and thought it would sufficient to distinguish any samples that might be different due to importation events or geographic isolation. We also achieved similar results implementing a neighbor joining clustering method.

We now recognize the limitations to those approaches and have implemented DAPC as suggested by the referee. We agree that a population genetic method is more appropriate than even an improved phylogenetic tree algorithm. The results from DAPC support the previous conclusions that there is a significant overlap in genetic similarity between worms collected from humans and dogs. We have added a new methods section outlining this procedure and a new figure (S1 Figure). We again thank the reviewer for their suggestion.

(4) Section 0.5.2 The authors calculate an expected diversity in the sample based on the mutation rate/generation x the length of the mitochondrial genome. This is a mis-application of this kind of mutation rate—it is not a substitution rate. The mutations Denver et al. quantify occur during oogenesis/gamete development, and any expected diversity that could be calculated would be between a mother and her offspring. It can't be used simply to get an estimate of population diversity. The usefulness of the mutation rate estimate based on C. elegans for population genetics is that it can be used to parameterize coalescent models, which are extremely useful for a range of questions in population genetics and in some applications in phylogenetics.

Thank you for allowing us to clarify the goal of using this mutation rate to place our downstream choices in context. We used the mutation rate from C. elegans to approximate the number of variants in a barcode that could link direct transmission events (i.e. maternal and child pair) in the event of a spontaneous mutation. The extrapolation of acquiring less than one mutation per generation suggests two worms with a single base pair difference in the molecular barcodes are likely not connected by a recent lineage. We have revised section 0.5.2 to highlight our intended goal. We removed the statement equating an average number of variants per samples collected to the identity groupings since the expected mutations per generation is sufficient.

(5) Section 0.5.3 If I understand correctly, f is an average uncorrected pairwise distance between communities. This is a metric that has been previously described in the population genetic literature and should be cited.

Thank you for highlighting the inconsistent language. We have updated the method section language to be explicit that the pairwise geographical distance is the basis for the analyses and not a pairwise genetic distance. Sample pairs are grouped by exact identity or at least a single base pair difference, which differs from other genetic distance methods that account for degrees of differentiation within and between populations.

(6) Section 0.6 When comparing the diversity of COIII, cytb, and ND2-5 to other parts of the genome, there is no consideration of why variation may be higher in other regions of the genome based on genome annotation. The reason this is important is that some regions of the genome evolve differently than other regions. For example, regions that are non-coding (less of the mitochondrial genome than nuclear in animals) tend to have a higher diversity than coding regions. In contrast, some genes are expected to be under stronger selective constraints than

others. Genes that code for rRNA/tRNA tend to evolve differently than genes that code for proteins. Finally, primers for PCR-based studies are designed to bind to conserved regions to ensure robust and repeatable PCR across diverse samples. Thus, comparing the diversity as has been done here is not informative about future marker/barcode development. Rather than re-run these analyses taking into account how evolution is expected to act differently on variation in different parts of the mitochondrial genome, another option is to simply remove these analyses from the paper entirely. The take-home message is that sequencing more of the genome results in more information to distinguish worms and that some regions of the mitochondrial genome might be more variable than others, neither of which is novel or unexpected, and the same point could be made in the discussion by citing existing literature.

Thank you for this thoughtful response to this section. We agree that our results of finding more variants with a larger genomic range is not novel. We have framed the section to highlight the motivation for these analyses specifically for Guinea worm. Applying standard shotgun sequencing approaches is difficult in Guinea worm due to sample collection protocols that can result in degraded parasite DNA and microbial contamination.

The original loci (CO3, cytB and ND3-5) were targeted because of their variation and ability to amplify. It was unclear before our study whether incorporating more regions of the genome into analyses would justify a new protocol development, which requires time and financial support. In showing that the pairwise genetic similarity distributions differ when including additional coding and non-coding regions, it supports the recommendation for an improved protocol.

Reviewer #2: I find the methods confusing and not easy to follow. References are lacking for many of the methods used. It is also not clear as to how the Sanger sequences were checked and edited to rule out errors. The terminology needs to be reviewed in places to articulate what is an analysis term versus a biological term. For example "worm pairs" is used a lot and this must relate to between sample analyses rather than pairs of worms?

Thank you for bringing these points to our attention. We have added clarity to highlight that the measurements in our study are based on identity groupings correlated to true geographic distance, and genetic similarity is based on Hamming distances. We will refer to worm pairs as pairwise comparisons between worms to clear up terminology.

No ethics are detailed and as several of the samples are from humans this needs including in the methods. Also, anything related to live animals.

Thank you for this comment. The study uses national case surveillance data, de-identified case surveys, and extracted worms that are routinely collected from a confirmed case. We have added an “Ethics statement” section to the method section confirming extraction and data de-identification for human subjects.

Reviewer #3: Yes to all of the above except the last question. I see no ethical or regulatory issues.

Results
-Does the analysis presented match the analysis plan?
-Are the results clearly and completely presented?
-Are the figures (Tables, Images) of sufficient quality for clarity?

Reviewer #1: The results as presented are adequate.

Reviewer #2: I feel the results could be much clearer. There is some very good data but unless you have a modelling background then it is not easy to follow. The main outputs from this study will be of interest in terms of the biology of transmission and so the methods and results should be more tailored to that audience.
Thank you for this broad comment about readability. We have simplified language in the results section to improve clarity.

Sequence data should also be made available on a database such as Genbank.
The data been made available on Genbank and we have added accession numbers to the article under the new "Data Availability" section.

Reviewer #3: Figures, Supplementary Figures, and Supplementary Information:

Please see the marked up copy with specific suggestions. There are several typos within the legends and some suggestions (like the addition of scale bars to maps), that may improve the figures. Also, in the current formatting, I found the figures to be too small. For example, it was hard to see distribution of the dots in Figure 1a and 1c. In addition, the labels in S4 were hard to read.
We'd like to thank Reviewer 3 for their time and effort to provide thorough comments on grammar and typos that were missed during editing in the main manuscript and the S1 Appendix. We have updated the manuscript accordingly and improved figures where possible based on suggestions. Due to the close geographic proximity of samples in the study, increasing the size of the dots for each sample overpowered the ability to distinguish samples in certain regions in Figure 1. The replication of these geographic distributions in S6 Figure for individual barcodes helps counteract some of the limitations of capturing all samples in a single figure. Changing the landscape of S4 Figure to increase the host number labels did not fit page limits.

Conclusions
-Are the conclusions supported by the data presented?

-Are the limitations of analysis clearly described?
-Do the authors discuss how these data can be helpful to advance our understanding of the topic under study?
-Is public health relevance addressed?

Reviewer #1: The authors make a convincing case based on their analyses about the utility of parasite genetic data to inform strategies for elimination.

Reviewer #2: The conclusions are discussed together with the limitations but better structure is needed for this particularly as the limitations seem to far outweigh the conclusions.

Reviewer #3: Yes to all of the above.

Editorial and Data Presentation Modifications?
Use this section for editorial suggestions as well as relatively minor modifications of existing data that would enhance clarity. If the only modifications needed are minor and/or editorial, you may wish to recommend "Minor Revision" or "Accept".

Reviewer #1: (No Response)

Reviewer #2: See more detailed comments below

Reviewer #3:

Abstract
1) Background, consider hyphenating "ten-year absence".
The addition of a hyphen was to the abstract.

2) Methodology, specify "dog and human hosts" given the mention of paratenic hosts in the previous sentence.
The addition of "dog and human hosts" was added to the abstract.

3) Methodology, rephrase the last sentence to improve quality. A suggestion is in the attached manuscript.
Thank you for the recommendation. Sentence has been updated in the manuscript.

4) Methodology, replace "three loci on" with "three loci in"
The word change has been updated in manuscript.

5) Consider revising the last sentence to improve clarity. A suggestion is in the attached manuscript. Also, this part of the abstract makes the least sense as there is no reference to "worm

pairs” in earlier parts of the abstract. More of a transition to this part of the study in the Abstract would improve clarity.

Thank you for the suggestion for the last sentence. We added the motivation for the complete genome analyses as a transition for the results.

6) Conclusions/Significance, revising a sentence will improve clarity – see suggestion in the attached manuscript.

Thank you for the suggestion. The sentence has been updated in the manuscript.

Introduction

1) Line 8, specific that these losses are due to Dracunculiasis

Added “losses due to Dracunculiasis” to this sentence.

2) Line 10, hyphenate “decade-long”

The addition of a hyphen was to the introduction.

3) Line 27, hyphenate “genome-wide”

The addition of a hyphen was to the introduction.

4) Line 38, delete “directly”. Using genetics to track the movement of parasites is an indirect way of assessing movement. You are making inferences which is based on host sample size, parasite sample size, variability of loci and number of loci, etc.

Thank you for pointing out this distinction. We have changed “directly” to “indirectly” in this sentence.

5) Line 43, improve clarity by revising sentence.

Methods and Materials

1) Line 69-71, clarify what “samples overlapped” means – the individual worm was part of the previous study? Also, delete extra “.”

The sample overlap was in reference to samples that were a part of a previous study. We have updated the language to make this clearer.

2) Line 77-78 add the unit “bp” behind each reported range as the current formatting initially caused confusion.

The updated the sentence to specify genomic range in the manuscript.

3) Line 82, was comparing singleton counts among sequences the only method of quality control? Given that Sanger sequencing was used, were chromatograms examined for the sequences that were excluded?

Thank you for allowing us to clarify this point. Consensus sequences from chromatograms were obtained as previously described in Thiele et al 2018, referenced in the previous section. We did

have a few (4) samples with ambiguous calls, now mentioned in the section. These sequences were then aligned to the reference genome to confirm the correct sequence was targeted. The variants that were identified once in the population were checked against the worm that harbored the variant. Two worms with 50% of the singleton variants identified in the population in separate loci were excluded from variant discovery and analyses.

4) Can line 83 ("Singleton variants identified in these two samples were excluded") be deleted if these two sample were excluded (see previous line)?
Simplified with the above clarification.

5) Line 84-85. Why was the position missing a base call? Was this a quality issue or a deletion or insertion?
Thank you for bringing up this point about our definition of missing. The ranges in this study were defined by the alignment ranges of all samples. A missing base call is due to technical variation of amplification and fall at the start and end of ranges. We have updated this section with this detail, with clarification that positions with a missing base or an ambiguous call in more than 2 samples were not considered candidates for the molecular barcode. Indels were not observed otherwise.

6) Line 90, revise the subheading to help remind readers why this part is included in the study.
We have added the motivation for this section in the results.

7) Line 92, revise this sentence. A suggestion is provided in the attached manuscript.
Thank you for the suggestion. "Identified as Dracunculus" was added to this sentence.

8) Line 149, it is not clear what constitutes a "data-driving, empirical form".
Thank you for pointing out that this was unclear. This sentence has been updated to be direct that the function form is derived from the pairwise geographic distance between identical and non-identical barcodes.

9) Line 179, what does this symbol mean, or is this a requirement of the journal? Replace with "see section 0.5.4?" Do this in other places is applicable.
The symbol has been updated to "see section" throughout the manuscript.

10) Line 203, clarify what "population worm cases" are. How does one go from 459 to 425 worms?
We have updated "population worm cases" to be clearer with "worm samples with matched coordinate data". The drop out of samples from the genetic comparison to the spatial analyses is described in Section 0.7.

11) Line 217, add a unit here to clarify that this is bp. Also, is the reference to PlasmoDB correct? If so, more explanation is needed.

Thank you for catching this reference error. The gene coordinates were pulled from ENA refence genome used for alignment. We have corrected this in the manuscript.

12) Line 238-252, the methods associated with the barcode accumulation curve analysis should be included in the Methods section rather than the Results section to foreshadow the result.
Thank you for the suggestion. We believe these accumulation curves help guide our recommendation to sequence all available worms but constitute a separate section to remain as the supplement. We have added a reference to the accumulation curve supplemental material in the methods section 0.3.1 to preface the methods prior to the results.

13) Reorder the methods to follow the order of the Results section. Put 0.2.2 last.
Thank you for the suggestion. While moving the methods for the complete mitochondrial DNA section does flow with the results, we paired "Alignment and variant identification" into a single section to ensure that the differences between calling variants were easy to compare.

Discussion

1) Line 357, expand on why it is important to recognize diversity – especially this type of genetic diversity "mtDNA) in the discussion.

Thank you for bringing this to our attention. This sentence was meant as a precaution for imposing interventions in boundaries identified from this study might not be sufficient for barcodes that have a broader range. We have rewritten this sentence to be more explicit on this point.

2) Line 396, replace "lead" with "led"
This typo has been corrected in the manuscript.

3) Line 404, move "other" to in front of "parasites".
The placement of "other" was updated in this sentence.

4) As someone who works on non-model parasites, I was curious as to what constitutes "a tailored set of phylogenetic or phylodynamic methodologies".
Thank you for allowing us to clarify this point. This sentence was a preface the unknown mutation rate to build time-corrected phylogenies. We have removed this sentence from the manuscript to eliminate redundancy.

5) Line 423, What is "between resolution". Consider revising as "better resolution of genetic similarity between Guinea worms in Chad. Why would this only improve the resolution in Chad? Wouldn't it improve it analyses of genetic similarity in other countries too?

We have updated our manuscript to improve the idea of better resolution. We mean that there are different distributions of genetic similarity, not a shift in the distribution, suggesting more sites

will change how samples are related. We agree more sites would improve any Guinea worm analyses in any country from principle and have removed “in Chad” from this statement.

Summary and General Comments
Use this section to provide overall comments, discuss strengths/weaknesses of the study, novelty, significance, general execution and scholarship. You may also include additional comments for the author, including concerns about dual publication, research ethics, or publication ethics. If requesting major revision, please articulate the new experiments that are needed.

Reviewer #1: In this paper, the authors use an impressive number of samples from Chad to explore the relationships of genetic variation to geographic variation with the goal of building a model that can be used to estimate the risk of recrudescence should apparent elimination of Guinea worm in humans be achieved. This is an admirable and excellent goal, and the introduction and discussion are well-written and make an excellent argument for genetic epidemiology as a valuable approach. The authors convincingly build a case that identifying correlations between genetic diversity and geographic distance has practical applications for elimination of parasites. This research is a good first step.

However, the paper lacks diversity in citations of other literature on population genetics of parasites, primarily relying on previously published work on Guinea worms, and this is reflected in some problematic aspects of their analysis. There are some analyses that would need careful attention before I would recommend publication of the manuscript as-is. In particular, the methods used to generate the phylogenetic tree are inadequate, and the data need to be re-analyzed or replaced with a population genetic approach for answering the same questions (i.e., regarding dog and human sample clustering and changes in genetic diversity over time); the exploration of genetic diversity in the mitochondrial genome as a whole will need additional analysis to add more depth, or could be removed entirely.

Reviewer #2: This is an important study that aims to investigate the transmission dynamics of Guinea Worm to support eradication efforts. There is a large dataset analysed providing important and novel data, however the paper lacks clarity and is hard to follow with the key points lost within the analyses. Details are shown below and I suggest the authors revise the paper to make it more clear to the non-modelling audience.

Abstract
- worm pairs ? do you mean samples or worm pairs.
We have updated all instances of “worm pairs” to “pairs of worms” in the manuscript.

Line 16: re-phrase the line. Genetic sequencing is not a tool for control but supports control through the understanding of transmission. Also change “Genetic sequencing” to “genetic analysis”. Sequencing is just one of many methods.

“Sequencing” was updated to “analysis” in the manuscript.

Methods
A section on sample information is needed. E.g. what were the hosts for the 712 worms ? Dates of collections etc. A supplementary table would be useful.

We include the year and host for the 459 worms that were successfully sequenced at three loci and included in the study as summary Figure 1A. Specific details about cases, such as dates tied to names, need approval from the CGWEP .

Lines 66-71 – make it more clear about the data from the samples as it is currently confusing. Maybe add a table of what DNA regions are available for each region. Also, was the data from reference 10 used in the analysis or was new data obtained from the samples used both studies. If the data was used then accession numbers need to be included etc. Make this much more clear. Add more information about how the data was obtained and edited. It is in reference 10 but good also to include some basic information and not just the reference. Include the loci etc. In the introduction it should also be stated that you are building on the data already analysed in reference 10.
The 459 samples included in variant discovery had successful sequencing at the CO3, cytB, and ND3-5 loci as mentioned in Methods 0.3.1. We have included the accession numbers for all previously published and new samples in this study user the “Data Availability” section. We mention the inclusion of previously published samples in the concluding paragraph of the introduction.

Line 74-89 – A typical sanger sequencing approach that the data was generated by allows for sequence editing and checking using sequence chromatograms. Can you confirm that this was done for the sequence data prior to alignment and allow you to produce contigs for each sample which can then be analysed. The methods described are confusing e.g. what do the genomic ranges relate too ? why is there missing data ? missing data would be due to sequence error and not as a variant ? you would not normally see deletions within a species ? What do you mean by singletons and why are some discarded and some kept ? I am also assuming that your barcode gives you a genotype so you identified 41 genotypes within the 459 samples ? It might also be worth investigating the SNP’s in terms of synonymous versus non-synonymous mutations. Was there also clear differences in the frequency of the different genotypes.

Thank you for this thoughtful comment. We have expanded the analyses to summarize calling methods from chromatograms as described in Thiele et al 2018. The ranges refer to the genomic location on the mitochondrial genome that the reads spanned after alignment to the reference genome. Missing positions are driven by amplification and alignment variation at the start and end of the which were identified from the population. We had a few samples (4) that had ambiguous base calls which we have now mentioned in this section. We were previously considering any non-ATCG base as “missing” but has been updated more details. With the described exclusion of positions with more than two missing or ambiguous positions as

candidates, we eliminate the variable start and end site positions in the molecular barcode. We do not observe indels in other regions of the gene.

We also added details of how true singleton variants were distinguished from noisy variants; six true variants were retained in the molecular barcode. The barcode is a genotype representation of variants identified these three loci.
We agree there is interesting biology regarding the type of mutations identified, however for modeling groups of identical and non-identical barcodes, we would treat a variant in either mutation class similarly. If I understood correctly about genotype meaning molecular barcode from this comment, there are differences in the frequency of barcodes. The frequency of different barcodes by host species is provided in S1 Table.

Lines 90 109 – so you need to explain that this is NGS data as this is annotated and aligned in a different way to the sanger data, particularly in the way that errors and quality is assessed. This data also needed to be included in some sort of sample table showing what data comes from what sample and what methods etc. It is unclear if the whole mt genome was used in the analyses or just the target genes.

Thank you for allowing us to clarify the sequencing methods. The information for these samples can be found in the ENA accession metadata for the Durrant et al. 2020 for mitochondria samples from Chad. We have provided a link to the Git repository with the sample accession numbers to the “Data Availability” section. These samples were not included in the construction of the molecular barcode and spatial analyses; they were independently assessed to investigate the value of additional markers in Guinea worm. The complete genome was used in the analyses regarding variant discovery and pairwise genetic similarity of the population. We compared the pairwise genetic similarity calculated from variants found in the amplified genes, outside the amplified genes, and all variants.

It is not clear why the barcode method is better than a full sequence analysis, with model selection, bootstrapping, AA changes etc.
Following a recommendation from Reviewer 1, we have dropped the phylogenetic analyses from the supplement and pivoted to another method that achieves the goal of barcode comparison between hosts.

Line 131 – this GPS data should also be added to the sample master file so the full data is available.
Thank you for this point. The GPS data are protected under data sharing agreements by the country of Chad. We will provide a matrix of the geographic distance between pairs that are necessary for the spatial correlations to be transparent within specified limits.

Line 139 – refer to a table that details to number of worms analysed per host and what the hosts were.

We specify 65 of the 245 hosts in this study had multiple worms in Results Section 0.10.1. The hosts with multiple worms are plotted in S4 Fig for reference.

Line 144 -explain what you mean by yearly transmission cycle. Is it to do with pre-patent periods or seasonality etc.
Thank you for the clarification on the yearly transmission cycle. We updated this sentence to be clearer about the yearlong pre-patent period of Guinea worm infection.

Spatial Analysis – would the spatial analysis be based on water bodies that are connected to allow spread not just distance between water bodies that allow movement of hosts between water bodies.
This is an excellent point based on the transmission mode of Guinea worm. We only have information on the village coordinates upon case confirmation. We do not have enough epidemiological information about movement of humans and dogs to infer the body of water that may have caused the infection.

Line 169 – you refer to sequencing error here. Sequencing error should be very low for the Sanger data so it is confusing as to why more sequence editing was not done. Also, could a genetic clustering analysis (e.g. a haplotype network or similar helps with grouping population clusters which could then have been analysed spatially. Also was the clustering analysed in terms of host ?

Thank you for bringing up this point. Sequencing error should be very low for Sanger sequencing. DNA degradation can be an issue in Guinea worm. The sequences were aligned to the reference genome to confirm we had targeted the correct regions for each worm across all loci. No sequences were edited in this study, but ambiguous calls were checked. Given the short time scale for the analyses over a few years in relation to the expected mutation rate in terms of lineage identity, having 100% identity is ideal for transmission tracking within the limitations of mitochondrial DNA. In sensitivity analyses, we allowed for a single base pair difference to be considered for identity to account for potential sequence errors (despite an expectation for this to be low) and/or a potential underestimation of the mutation rate that is unknown in Guinea worm.

Analyses for human and dog hosts were computed separately but not included in this study. There are few human host samples in this study; we are not adequately powered to perform the analyses on humans between identical and non-identical barcodes. Dog only analyses mirrored the analyses presented in the manuscript because they dominate the sample population. Please see the results below for dogs only.

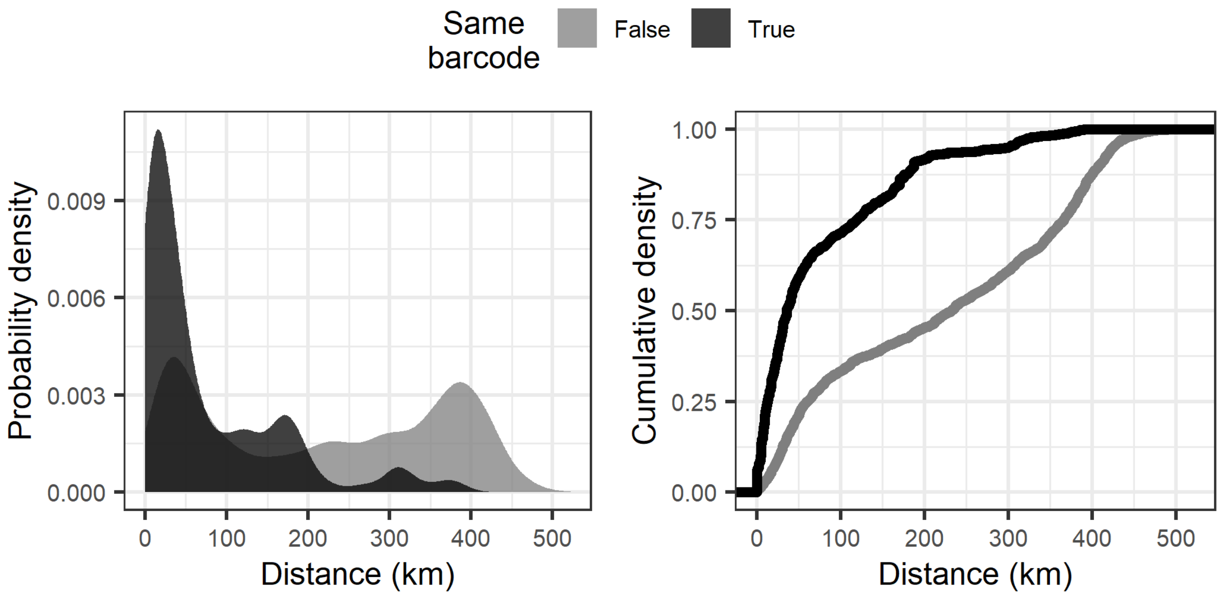

Line 194 – refrain from using the term worm pairs as this suggests male and female pairs. Use a different terminology.
Thank you for bringing up this terminology distinction. We have updated instances of "worm pairs" to "pairs of worms" throughout the manuscript.

Sensitivity analyses – is this the correct term or should it be statistical analyses.
Thank you for this point. While all the methods in the paper are statistical analyses, the sensitivity analyses are those that test the assumptions of our barcode grouping and other epidemiological features that may confound results.

What does distance vector relate to – is it the disease vector (copepod). Do try and help the readers that are not modellers.
Thank you for bringing up this point. We have changed that sentence to be clear that the distance vector is the all-by-all pairwise geographic distances.

Paragraph 202 – are there references to these methods used. You also need to be more clear on how this is done and refrain from complexed terminology e.g. what is the index worm and are the population worm cases just samples ?
Thank you for highlighting the complexity of this section as originally written. We have rewritten this section to remove specific terminology and reference that a technique is applied to each worm in the study.

Paragraph 214 – more references need to be included for bit of the analyses used. It is difficult to understand what data the variants outside of the loci is based on ?

Thank you for allowing us to clarify this point. We have expanded the section to clarify that variants outside loci are coding and non-coding regions that are not in the CO3, cytB, and ND3-5 loci used to characterize Guinea worm in the spatial analyses of the study.

Line 232 – Figure 1A says number of sequences per host for each year but this should be number of samples analysed there will be more than one sequence for each sample?
We have updated the figure to reflect the number or sequenced samples per year.

Line 234 – case coordinates means geographical location of the infection ? make this more clear.
Case coordinates refers to the geographical location the reported case after emergence; the location of the infection is unknown. This sentence was updated for clarity.

Line 241 – this Binomial analyses should be detailed in the methods ?
We have added a reference to the appendix containing the binomial analyses to the methods section 0.3.1.

Line 267 – the analyses are based on mitochondrial DNA so diversity will be high and increase with sample size as seen in the dogs. Based on the mt DNA you do not know if the worms are genetically identical, they are the same genotype based on maternal inheritance but you cannot tell if they are clones ?
Thank you for bringing up this excellent point. We do not know if worms in a pair are genetically identical beyond the variants identified in the sampled population at these positions. Will update to state identical barcodes.

Line 278 – what is a sequencing mutation ? do you mean error ?
We have updated "mutation" to "error" in the manuscript.

Line 309 – this is the information that was needed in the methods.
This information is replicated in the methods.

Figure S4 – add better scale to the A – is the lower limit 1 – also the last host has lots of worms – this kind of extreme should note some discussion. The Y axis numbers are also unclear. For B use yes and no instead of false and true as false tends to mean unreliable.
Thank you for the suggestions to improve our figure. We have specified plot legend the graph only contains hosts with more than one infection. The Y axis is the number of sequenced worms collected per host. Figure B has been updated from True/False to No/Yes.

Figure S6 – you have non common barcodes shown too so change the figure title.
Thank you for catching the not common barcodes exclusion in the title. We have updated the title to specify the non-common barcode geographies also.

Line 326 – do you mean single variants – variants found in only one worm suggests you get variation within the worm ?

We have updated this sentence to be clearer that we are repeating the analyses without variants identified in a single worm in the population.

Line 327 – I think you need to make it more clear what you mean by within and outside loci.
The distinction for with and outside loci have been added to method section 0.6.

Line 343 – you did not analyse 459 sequences but 459 samples ?
This sentence has been updated to samples instead of sequences.

Line 345- is quantitative bounds the correct term ? do you mean boundaries ?
Bounds has been updated to "boundaries" in the manuscript.

In your discussion on limitations why was a full phylogenetic analysis not performed on the data and it was just based on the mutation barcode – this could have provided resolution to the analyses.
Due to the low mutation rate of the mitochondrial genome, we originally chose to build a tree from the variants due to partial clonality of the molecular barcodes. However, we have pivoted to a better population genetics approach to compare the barcodes between host species and have removed the phylogenetic analyses from the supplement.

Also data should also be made available through a data base such as Genbank.
We have uploaded the sequences to Genbank and list the accession numbers in the new 'Data Availability' section.

I think the discussion could be tightened to show the clear findings better. There are clearly many limitations to the study and these are discussed but I do not find the discussion well structured.

Thank you for this suggestion. We balance the limitations of the marker set used to characterize the population with sensitivity analyses to support the conclusions while providing evidence for other sequencing technologies. We believe the current structure will allow individuals that guide programmatic decisions to evaluate the strengths and current limitations of genomic surveillance in Guinea worm.

Generally the paper shows important findings and advances the needed analyses for understanding the animal reservoirs for or the zoonotic transmission of Guinea Worm but the paper is very hard to follow, with non-uniform terminology and also terms that mean several things e.g. could relate to the biology of the parasite or the analyses. The authors need to consider the audience and provide clearer methods and results.
Thank you for this comment. We have updated various portions of the paper for clarity to address inconsistent terminology between fields and simplify methods to improve readability.

Reviewer #3: This manuscript builds upon previous molecular-ecology approaches to better understand the distribution of genetic diversity of Dracunculiasis in Chad. The dataset is expanded by considering more loci and more samples in space and time. The results show that worms with the same mtDNA multi-locus barcode tend to be clustered within a relatively small geographic range (within 50 km). However, these same barcodes are not partitioned by year or by host suggesting some fluid transmission dynamics. These findings are the result of comprehensive analyses and complimented by a further exploration of what these patterns would be like if the entire genome was analyzed. Perhaps unsurprisingly, modelling with genomes suggested that more loci would give better resolution into the genetic relationships among samples and would improve the resolution that genetic data could provide for programmatic surveillance and decision-making.

This manuscript stands out for its comprehensive set of analyses to investigate the validity of its various results. Also, I enjoyed reading the Discussion section. I recommend publication pending minor revisions. I included a list of these comments, but also copies of the MS and the supplemental materials with my comments annotated throughout.

Editor Revision Round 2

I have flagged this in the system as “major revision”, but that might be excessive. Some specifics. But you need to read the entire paper for flow and consistency. It remains verbose and could benefit from skilled editing.

I’ll refer to line numbers in the document PNTD-D-20-01771_R1_trackedChanges.pdf.

We’d like to thank the editors for guiding further improvements of our paper before sending for a second round of review. The new tracked changes document, which includes many of the recommendations below, is in reference to the original submission.

Abstract (no lines numbers for the abstract in the document).

Wrong use of the word “paratenic”

Thank you for highlighting the use of paratenic in this sentence. The rise in dog cases suggested that there may be another paratenic host (e.g. fish or frogs) that are facilitating transmission.

Are you sure you mean “…genetically identical worm populations…”?

Thank you for this comment. Genetically identical worm populations refers to worms that share an identical molecular barcode. We have included ‘genetically identical’ after the description of genotypes to clarify that identity is defined by the genotypes from the molecular barcode.

The sentence “we investigated the value of…” should be reviewed. And it is hardly surprising that the addition of a longer region of the mt genome will distinguish more lineages.

We agree that capturing larger portion of the genome will uncover more combinatorial variants to define a lineage. The main manuscript was expanded to include the motivation for this work; it supports the value of protocol development to include more markers than a novel insight. We have updated this section of the abstract to include “as expected” and the protocol development.

In the third line of the “conclusions/significance” section of the abstract, is “co-occurrence” better than “overlap”? I have trouble envisaging two worms overlapping.

Overlap changed to co-occurrence.

Line 23. You talk about “phylogenetic methodologies…”. But the phylogeny previously in the paper has been removed. Do you want to retain this part of the introduction?

Thank you for highlighting this point. While the phylogenetic analysis is not relevant for our point of tracking recent transmission, this referenced literature does highlight the relatedness of worms in Chad on larger time scales.

Line 66. Something is missing after “successfully”. Should it be “amplified”? “sequenced”?

Added amplified and sequenced to this sentence.

Line 70. Maybe “reported in previous literature”?

Updated “included in” to “reported in”

Lines 75, 98 and elsewhere. The adjectival form “mitochondrial” would be better.

Mitochodria update to mitochondrial in the manuscript.

Senetence starting on line 77. Is it needed? The sentence that follows seems to provide necessary information.

Line 78. Phrasing. I would say “The regions sequenced were from sites 3,690 to 4,308 for CO3…etc.” See also around lines 250-253.

Sentence updated to remove introduction sentence and specify genomic ranges.

Lines 84-93. Here, the attempt to clarify the way in which singletons were dealt with has not made things much clearer. Please revisit this section. I quite like “inflated singleton counts”, which you have removed in this revision. The next few points also relate to this section.

Thank you for highlighting this point. We expanded this section to provide further detail on candidate variant positions and included “inflated singletons” and their definition in the explanation.

Line 86. The sentence starting “Non-singleton variants were retained if…” confuses me. If a base (variant?) is present in "all but one sample", then surely that one sample represents a singleton?

Line 87. The sentence starting “Four samples had an other position…” does not make much sense.

Thank you for bringing up these points on lines 86 and 87. We have expanded on the details of this section to specify an “other” designation at a position (true missing due to amplication variation at start and end of gene ranges, or an ambiguous call). The “all but one sample” was referring to additional filtering of candidate variant positions after the removal of inflated singletons where a sample had more than one missing base or ambiguous call at that position. We allowed for two samples in the population have the designation of other to allow for both a missing and ambiguous call at one site, which has been corrected in the manuscript. This allowed us to retain informative variant positions in the barcode for the population while allowing some flexibility for technical variation in a minority of samples.

We coded ambiguous and missing bases similarly in the barcode and have corrected that the “missing” bases are ambiguous base calls and not deletions from the Sanger reads. We updated the sentence describing that 4 samples had an ambiguous call in a position in the barcode that represent 3 barcodes in the study. This was the highlight that the barcodes does not contain missing positions, and a small fraction of samples (<1%) were affected by technical variation.

Line 89. “These criteria”. Given the new material added to the paragraph, the reader might have trouble working out what criteria these are.

We removed “These criteria” from the revision because the sentences describing the positions, removal of inflated variants, and allowing for the inclusion on an ambiguous base is described.

Line 92. Remove “see”.

Removed additional word from sentence.

Line 95. Surely it is not Dracunculus (note spelling and lack of italics) samples that were publicly available, but rather Dracunculus mitochondrial genomes?

Thank you for this correction, it is Guinea worm mitochondrial sequences. The sentence has been updated.

Line 126. Rather than “of the molecular barcodes” I would say “based on the molecular…”

Updated to “based on the molecular…”

Line 131. Should the word not be “discriminant”?

Updated discriminate to discriminant.

Line 159. This sentence needs attention. I don’t think you can refer to a “pre-patent cycle”. Maybe something like “Here, given the annual life-cycle of guinea worm, …”

Thank you for this point. We followed a suggestion from reviewer 2 to clarify that the prepatent cycle is indeed the correct terminology since we are specifying the time between host infection and skin breakthrough by the adult female worm to release eggs.

Line 164. “…is a the…”?

Extra word “a” removed from the sentence.

Line 170 and elsewhere. How often do you need to say “the functional form of f”? I found the phrase at least three times. And should “f” be in italics or not? Be consistent.

Thank you for noticing this repetition. We have removed the extra instances of this phrase and removed the italicization of f to be consistent.

Lines 175-186. Present and past tenses both used. Reporting what you did, the past tense is better. This switching between tenses is throughout the Methods and Results sections. Please check and be consistent. Results should also be in the past tense: reporting what you actually did.

Thank you for pointing the tenses out to us. The manuscript has been updated to the past tense in the Method and Results sections. Additional updates in the

Line 205. The sentence starting “Note” is hard to follow.

The sentence has been rewritten for clarity.

Line 214. “criteria” is a plural form.

Updated criteria to criterion.

Line 222 “worms pairs”?

Worm pairs updated to pairs of worms.

Line 238.”The number … was counted…”

Were updated to was.

Lines 247-256. Revisit and try to increase brevity and clarity.

Thank you for highlighting the length of this section. We have rewritten this section.

Lines 2720273. “…for each reported case”?

“The” updated with “each” in this sentence.

Line 273. The map in Fig. 1 does not identify the Chari River.

Thank you for highlighting this point. We will continue to search for river shape files that meet the journal’s attribution requirements.

Line 360. I would replace “with” with “using”.

“With” updated to “using”.

Lines 399-403. Some of this seems to be stated also around lines 377-379. Check for repetition.

Thank you highlighting this repetition. The second instance of describing the distribution of pairwise similarity is the sensitivity analyses excluding singleton variants that held the same trend when including variants. We have added the new figure after the sentence to clarify that sentence that is does refer to a different analysis.

Line 413. Maybe “Knowing the geographical range of transmission”?

Added geographical to the original sentence.

Line 436. Maybe “…and this suggests…”?

This added to the original sentence.

Line 516. “Sequencing more historical samples could either decrease genetic diversity…”. I don’t follow this.

Thank you for bringing up this point. We have updated this sentence to highlight that sequencing more worm samples could either help with sample size estimation or support the need to sequence all worms.

Lines 521-524. This is an example of a very wordy sentence. Try to edit it (and others) to a more digestible form.

Thank you for highlighting a wordy sentence as an example. We have tried to edit similarly structured sentences with this guidance.

Fig 2B, caption. Would better phrasing be “For each barcode, pairwise distances among all pairs of worms with that barcode were calculated“?

Thank you for this suggestion. This figure does show the pairwise geographic distances on a per barcodes basis. The original caption specifies that each molecular barcode has a different number of pairwise comparisons used in the estimate due to uneven representation of barcodes in the study, as provided for the population analyses.

Fig. 3, caption. Maybe “… does not drive apparent spatial connectedness”?

Apparent added to caption title.
