# Supplementary material for: Linked surveillance and genetic data uncovers programmatically relevant geographic scale of Guinea worm transmission in Chad
Source: PLoS Negl Trop Dis. 2021 Jul 26;15(7):e0009609. doi: 10.1371/journal.pntd.0009609 (PMC8341693; doi:10.1371/journal.pntd.0009609)
Supplement: Attachment — Submitted filename: GuineaWormPhylodynamics_20210409_trackedChanges_Reviewer3.pdf [file pntd.0009609.s017.pdf]

Linked surveillance and genetic data uncovers programmatically relevant geographic scale of Guinea worm transmission in Chad

Jessica V. Ribado[1], Nancy Li[1], Elizabeth Thiele[2], Hil Lyons[1], James A. Cotton[3], Adam Weiss[4], Philippe Tchindebet Ouakou[5], Tchonfienet Moundai[5], Hubert Zirimwabagabo[4], Sarah Anne J. Guagliardo[4,6], Guillaume Chabot-Couture[1], Joshua L. Proctor[1*]

1 Institute for Disease Modeling, Global Health Division of the Bill and Melinda Gates Foundation, Seattle, WA, USA
2 Vassar College, Poughkeepsie, NY, USA
3 Wellcome Sanger Institute, Hinxton, Cambridgeshire, UK
4 The Carter Center, Atlanta, GA, USA
5 National Guinea Worm Eradication Program, Ministry of Public Health, N'Djamena, Chad
6 Centers for Disease Control and Prevention, Atlanta, GA, USA

* Corresponding author
Email: jproctor@idmod.org

Abstract

Background

Guinea worm (*Dracunculus medinensis*) was detected in Chad in 2010 after a supposed ~~ten year~~ ten-year absence, posing a challenge to the global eradication effort. Initiation of a village-based surveillance system in 2012 revealed a substantial number of dogs infected with Guinea worm, raising questions about paratenic hosts and cross-species transmission.

Methodology/Principal Findings

We coupled genomic and surveillance data from 2012-2018 cases to investigate the modes of transmission between dog and human hosts and the geographic connectivity ~~for genetically similar worm populations~~of worms. Eighty-six variants across three loci ~~on~~ in the mitochondrial genome identified 41 genetically distinct worm genotypes. Spatiotemporal modeling ~~reveals genetically identicalworms are~~ revealed worms with the same genotype ('genetically identical') were within a median range of 18.6 kilometers of each other, but largely within approximately 50 kilometers. Genetically identical worms ~~vary~~ varied in their degree of spatial clustering, suggesting there may be different factors that favor or constrain transmission. Each worm ~~is~~ was surrounded by five to ten genetically distinct worms within a 50 kilometer radius. ~~In an independent population, we show that more variants revealed in whole mitochondrial genome data improved the discrimination between worm pairs~~ As expected, we observed a change in the genetic similarity distribution between pairs using markers across the mitochondrial genome of worms in an independent population.

Conclusions/Significance

In the largest study linking genetic and surveillance data to date of Guinea worm cases in Chad, we show genetic ~~similarity~~ identity and modeling can contribute to understanding local transmission. The ~~overlap of genetically distinct~~ co-occurrence of genetically non-identical worms in quantitatively identified transmission ranges highlights the necessity for genomic tools to link cases. The improved discrimination between ~~worm pairs~~ pairs of worms from variants identified across the complete mitochondrial genome ~~indicates expanding~~ confirms that expanding the number of genomic markers could link cases at a finer scale. These results suggest that scaling up genomic surveillance for Guinea worm may provide additional value for programmatic decision-making critical for monitoring cases and intervention efficacy to achieve

elimination.

Author summary

The global eradication effort for Guinea worm disease has dramatically decreased the global burden of the disease and enabled 187 countries to be certified by the World Health Organization to be free of endemic transmission. However, several countries continue to have endemic transmission; in Chad, a long absence of reported cases was interrupted with the identification of Guinea worm cases. Surveillance and intervention efforts have substantially scaled up in Chad. Here, we study the value of increasing genomic surveillance as a tool for programmatic evaluation of surveillance and intervention efforts in Chad. Linking surveillance and genomic samples, parsimonious spatial models help reveal a consistent geographic clustering of similar genetic sequences across Chad. We also demonstrate that expanding the sequencing can offer better resolution for distinguishing Guinea worm samples. In this retrospective study, we found evidence that scaling up genomic surveillance can be an important monitoring and evaluation tool for the eradication program in Chad.

Introduction

The eradication campaign for dracunculiasis, Guinea worm disease, has made substantial progress since the first set of World Health Assembly resolutions aimed at elimination efforts passed during the International Drinking Water Supply and Sanitation Decade (1981-1990) [1]. The Guinea worm eradication campaign has decreased the global burden of disease by more than 99% [2], enabled the World Health Organization (WHO) to certify 187 countries as free from endemic ~~Dracunculiasis~~ dracunculiasis transmission [3], and decreased the significant economic loss associated with Dracunculiasis in rural settings [1]. Despite the substantial progress, endemic transmission has persisted in Angola, Chad, Ethiopia, Mali, and South Sudan [2]. In Chad, a ~~decade long~~ decade-long absence of reported cases was interrupted with a detected resurgence of Guinea worm cases in 2010 [4]; moreover, human cases continue to be reported [5] with a growing understanding of the role of animal reservoirs in transmission [6–10]. The stalled progress in Chad has led to a substantial increase in surveillance and programmatic efforts [11] as well as investments in research and novel interventions [12, 13].

Genetic ~~sequencing~~ analysis is an important tool for the control of other pathogens. In the case of polio eradication, for example, these tools have been used to detect silent transmission across geographic areas and thus inform programmatic decision-making [14–16]. The inclusion of strain differentiation techniques into programmatic decision-making for poliomyletis is feasible due to the continual mutation events in the virus genome, a growing global library of samples and isolates, and a mature set of mathematical and statistical methodologies to track specific lineages. For parasites, such as Guinea worm, well-established phlyogenetic methodologies to infer high-fidelity ancestry and lineages do not yet exist [10, 17]. Nonetheless, genetic data collected from Guinea worms in Chad has already revealed research and programmatically relevant insights: human and dog hosts share a common genetic population suggesting transmission between species [10]. ~~Genome wide~~ Genome-wide data from a much smaller sample of worms confirms this finding [12]. We build upon those research insights using linked genetic and surveillance data from Chad to investigate the geographic connectivity of genetically defined worm populations.

Spatial epidemiological models have been a key tool for inferring the connectivity of different populations and providing insights into the propagation of infectious diseases. Spatial models, such as the gravity [18] and Levý flight [19], have been utilized to describe the movement of humans and animals as a key component to the spread of

infectious diseases [20–26]. These models and sophisticated inference algorithms have helped characterize the movement of pathogens and provide insights for programmatic decision-making [27, 28]. Ge[illegible]etic data has the potential to bypass the need for proxy human movement data by ~~*directly*~~*indirectly* using ~~the movementof pathogens via the genetic linkage and geographic metadata~~pathogen movement [29] even in a partially sampled transmission network [16, 30, 31]. Using genetic and epidemiological data to inform infectious disease models is a growing field, which encompasses phylodynamics [32–35]. Similar to phylogenetic methodologies, phylodynamic analyses and models are most mature for virus and bacteria pathogens [16, 27, 30, 31, 36]. However, there have been recent innovations of using the biological characteristics of parasites clonally propagating in order to build phylodynamic models of malaria parasite movement within neighborhoods of Thiés, Senegal [37]. We demonstrate that probabilistic spatial models informed by Guinea worm genetic data can reveal new insights into the geographic connectivity of worm populations in Chad.

In this article, we investigate the programmatic potential of leveraging genetic data for enhanced surveillance efforts by performing a retrospective analysis and modeling of the epidemiological and genomic data collected in Chad from 2012-2018, excluding 2014. We leverage both previously reported [10] and new genetic sequences linked with surveillance data to build spatial models that reveal a consistent geographic connectedness of worm populations in Chad. ~~We also demonstrate the additional~~ We also confirm the value of expanding the number of l[illegible]equenced for Guinea worm in order to provide higher resolution in linkages between samples. These results are followed by a discussion on the implications of increasing the scope of genetic sequencing for programmatic surveillance and decision-making.

Materials and methods

0.1 Ethics statement

The World Health Organization and the country ministries of health have sanctioned the extraction of emergent adult worms to contain and treat Guinea worm infections. Trained program and ministry staff extract female worms from active cases and are stored in ethanol as described in Eberhard et al. [53].Samples from human cases are anonymized prior to study inclusion.

~~Materials and methods~~

0.2 DNA extraction and sequencing

Extraction was attempted on 712 worms collected in Chad from 2012-2018, excluding samples from 2014 due to availability. Whole genomic DNA was extracted from 5-15mm sections of adult female worm tissue with one of two methods: a modified Puregene DNA extraction protocol as detailed in Thiele et al. 2018 [10], or with the DNeasy Blood and Tissue Kit (Qiagen, Germantown, MD, #69582) according to the manufacturer's protocol for tissue extraction. Five hundred and ninety-five samples ~~were successfully sequenced~~ had successful amplification and sequencing for at least one ~~locus on the mitochondria genome~~of the three targeted mitochondrial loci (CO3, cytB, and ND3-5). Sanger sequencing and base calls from chromatograms were performed as detailed in Thiele et al. 2018 [10]. ~~There are 461 samples with~~ Four hundred and sixty-one samples had successful sequencing at all three targeted loci ~~(CO3, cytB, and ND3-5) in our study~~. Thirty three of these samples ~~overlapped with previous studies on~~ were reported in previous literature on the genomic characterization in Chadian Guinea worms ~~.~~ [10]. The remaining 428 samples have not been published previously.

0.3 Alignment and variant identification

0.3.1 Targeted CO3, cytB, ND3-5 loci

Sequences ~~of targeted loci~~ for CO3, cytB, and ND3-5 for 461 worms were aligned to the *Dracunculus medinensis* mitochondria genome version JN555591.1 from the European Nucleotide Archive with the BWA v0.7.17 software package [38] ~~. Genomic ranges for each locus were determined from the start and end positions of all samples. Ranges were~~ to confirm amplification of the correct loci. Amplification fragments spanned the genomic coordinates of 3,690 ~~-~~to 4,308 for CO3, 2,628 ~~-~~to 3,234 for cytB, and 12,562 ~~-~~to 14,523 for ND3-5. ~~Four hundred and sixty-one samples with successful alignment at each locus were considered for variant discovery. Alleles~~ Bases (A, C, G, T, or other for ambiguous or missing) were counted for each position in the ~~identified ranges . To avoid artifacts from poor sequencing or alignment, singleton variants were recorded with their respective sample. Two samples with inflated singleton counts in cytB and ND3-5~~ alignment ranges for the population. Missing bases were concentrated at the beginning and/or ends of the gene ranges due to amplification variation. Positions in the alignment ranges were excluded from ~~this study. Singleton variants~~

~~identified in these two samples were excluded. Six singleton variants were identified in non-excluded samples. Remaining variants were retained if a position was missing a base call for at most one sample. Four samples had a missing position in retained variants. These criteria~~ variant discovery if more than two samples with successful amplification for that gene had the designation of missing or ambiguous.

Positions with at least two different bases (excluding other) were variant candidates and checked for irregularities. Two worms samples exhibited highly irregular singleton characteristics, contributing more than 50% of the singleton variants in a single gene. These two worm samples were excluded from variant discovery and analyses. From the remaining worms, we identified 86 variant positions concatenated to create a molecular barcode. Forty-one unique barcodes were identified across 459 worms. Six variants in the barcode were singletons in the population. Three barcodes contained an ambiguous base 'N', assigned to four samples in the population. Barcodes were assigned an identifying number based on the number of samples belonging to each barcode. For methods describing the accumulation of barcode diversity in the population at different loci refer to S1 Appendix.

0.3.2 ~~Untargeted complete~~ Complete mitochondria genome

~~Nineteen publicly available , untargeted mitochondria DNA sequences~~ Next-generation shotgun sequencing from nineteen publicly available *Dracunculus medinensis* mitochondrial genomes were downloaded from the European Nucleotide Archive (PRJEB1236) [12]. Low quality bases with a minimum mapping quality of 20 were trimmed from the ends of reads. Trimmed reads were aligned to the *D. medinensis* mitochondria genome with the BWA v0.7.17 software package [38]. Variants were called on de-duplicated, uniquely aligned reads following best practices outlined by GATK v4.1.4. Known variants are typically recommended to correct sequencing errors that lead to spurious variant calls. A set of known variants is not available for the *D. medinensis* mitochondria genome. Instead, bootstrap base recalibration was done using higher confidence calls (QD $>$2.0, FS $>$60.0, MQ $<$40.0, MQRankSum $<-$12.5, ReadPosRankSum $<-$8.0) until the final calls converged (2-3 steps) [39]. Final variants were filtered using the following input parameters for GATK: FS $<=$ 13.0 or missing, SOR $<$3.0 or missing, and $-3.1 <=$ ReadRankPos, BaseQRankSum, MQRankSum, ClippingRankSum $<=$ 3.1 [12]. Given the low sample size for recalibration, orthogonal variant calling was performed using the bcftools program [40]. Genotype maximum likelihoods were determined using the mpileup command with parameters specifying a

minimum mapping quality of 20 and ploidy of 1. For both variant call sets, variants found within positions 5950-6670 were removed due to high heterozygosity and 2× mapping coverage. For all other variants, a minimum depth of 10 reads was required across all samples to be included in downstream analyses.

0.4 ~~Phylogenetic analysis of worms collected from humans and dogs~~

~~Phylogenetic trees were constructed using the ultrametric unweighted pair group method with arithmetic mean (UPGMA) [?]. We assumed that worms were evolving at similar rates and each base frequency is equally likely at each position. Jukes Cantor distance, the simplest substitution model meeting our assumptions, was used to calculate genetic distance between barcodes. Samples were clustered using agglomeration by average distance. Genetic distance and UPGMA clustering were calculated with R package phagorn v2.5.5 [?]~~

0.4 Clustering of molecular barcodes

Discriminant analysis of principal components (DAPC) was used to infer population structure based on the molecular barcodes using the R package adegenet v2.1.3 [41–43]. Populations were defined by host identity. Barcodes found in samples from humans only were grouped with barcodes found in samples from humans and dogs to create more even groups (humans only = 5 barcodes, both = 12, dogs only = 24). To investigate whether the barcodes differentiated by host species, we specified one discriminant axis for the two classifications. We tested the inclusion of 2, 5, 10 principal components on discrimination results. The results were comparable across the tested principal component range, with overlapping distributions in the first discriminant axis.

0.5 Linking genomic samples to epidemiological data

We linked the sequenced samples with corresponding surveillance data using national case reports and standardized surveys, described in detail in [44]. Case data was collected from an active and passive village-based surveillance system by the Chad Guinea Worm Eradication Program (CGWEP) and the Ministry of Public Health (MOHP) [45]. Summary case reports at the village level ~~are~~ were compiled across surveillance sites by the CGWEP program. Information on infected dogs was collected

through a standardized survey with the self-identified owner. Where available, global position system (GPS) coordinates from the standardized surveys were validated with village coordinates from the national case reports. The GPS coordinates were consistent across national case reports and standardized surveys for 207 samples. In instances where coordinates differed between the standardized surveys and the national case report, the national case report coordinates were assigned (181 samples). For cases reported with a village name in the standardized survey but without GPS coordinates, the GPS coordinates associated with the village name in the national case report was assigned to that sample (2 samples). For cases reported with a village name and GPS coordinates, but the village name was not in the national case report, standardized survey case coordinates was assigned to that sample (37 samples). Four hundred and twenty-six worms were linked across 245 hosts. ~~Sixty-five of the 245 hosts were infected by more than one worm. Multi-infected hosts had an median of four parasites, however, up to 24 parasites in a single host were observed.~~

0.6 Probabilistic spatial models of Guinea worm connectivity

0.6.1 Spatial models

Spatial movement models have demonstrated that distance is an important factor influencing the spread of pathogens. [21–23, 30]. Here, due to the yearly ~~transmission~~ prepatent cycle of the Guinea worm parasite [1], we ~~specify~~ specified a spatial model similar to a probabilistic diffusion model for a discrete spatial network representing the villages affected by Guinea worm in Chad. The model describes the probability of a worm being transmitted from village i to village j based on ~~pairwise genetic similarities f and distance between pairs~~ the geographic distance d between a pair of worms such that $p_{ij} = C_i \mathrm{f}(d_{ij})$~~where~~. C_i is a scaling factor and ~~f is a data-driven, empirical form directly computed from the Guinea worm data from Chad~~ f is informed by the pairwise geographic distance between pairs of worms with identical or non-identical barcode identity. Similar to other parametric formulations such as the diffusion, gravity [22], or Lévy flight models [30], our non-parametric formulation could be used to predict where a future linked Guinea worm case might appear.

0.6.2 ~~Pairwise genetic similarity~~ Genetic identity and geographic distance between samples

To infer ~~the functional form of f~~ f in the model above, we ~~utilize the genetic similarity~~ used the genetic identity between samples and the associated GPS coordinates for villages. ~~The genetic similarity is calculated for each unique worm pair as 1 - (number of discordant bases between barcodes/total number of bases compared) ; this similarity metric accounts for samples having a different number of resolved bases. Discordant bases were equally penalized (= 1) regardless of the base.~~We estimated the number of variant differences across the mitochondrial genome that may connect a direct lineage (i.e. a maternal-offspring pair of worms) from a spontaneous mutation rate. Without a known organismal mutation rate, we ~~assume~~ assumed a similar mutation rate as the *Caenorhabditis elegans* spontaneous mitochondrial mutation rate estimated at $1.05e-7$ site/generation [46]. From this rate, we ~~expect~~ expected 0.0015 mutations for the $14,628$ base pair *Dracunculus medinensis* mitochondrial genome between generations. ~~We have 86 variant sites across 459 worms, suggesting an average of 0.187 mutations per sample in the population. Both the average mutation per sample and the extrapolated mutation per generation from the *C. elegans* less than one, indicating a single base pair is sufficient to distinguish barcode sets.~~ Given this extrapolated generational mutation rat[illegible]s than one, worms were grouped by exact barcode identity. We did not make any assumptions of pairwise genetic distance from models of DNA evolution for analyses comparing genetic similarity between pairs of worms. We calculated the pairwise genetic similarity as 1-the Hamming distance from the molecular barcodes to obtain the number of shared variants [47]. The haversine distance was used to compute the geographic distance between each pair of worm samples d using the R package geosphere v1.5 [48].

~~Partitioning samples according to genetic similarity requires caution. Even grouping samples that are different by one base pair, either from a real mutation or sequencing error, creates an algorithmic issue. For example, if a genetic sequence that is one base pair different from two other sequences, but the discordance is at different base pair positions, then this three sample group would not be a single base pair different for all pairwise comparisons. In this work, we enforce that the groupings maintain the required base-pair differences by only including the pairwise comparisons that satisfy the requirement; note that this is different than grouping all of the samples with a base-pair discordance and then performing all of the pairwise comparisons. We use these relaxed similarity thresholds to identify shared identity between barcode~~

~~pairs to investigate the impact of geographic clustering (§0.6.4).~~ Grouping barcodes by exact identity may exclude pairs of samples that share a recent lineage in the event of an underestimated mutation rate or an unlikely, but possible, sequencing error. We tested the impact of a single base pair difference in barcodes between a pair of worms on geographic clustering (see section 0.6.4). Relaxing thresholds on a pairwise basis avoided issues clustering barcodes with more than a single base pair different for all pairwise comparisons. For example, barcodes A and B had two base pair differences, but barcode A and C and barcode B and C each have a single base pair difference.

0.6.3 Spatial models by molecular barcode sets

To characterize ~~the functional form f~~ f, we partition the samples according to their genetic ~~similarity~~identity. Samples ~~that are identical are grouped into molecular barcodes~~with identical barcodes are grouped; for each barcode k, all of the pairwise geographic distances within the set are used to compute a barcode functional form ~~f_k~~ f_k. The set of functions ~~f_k~~ f_k are then averaged to produce a single ~~f~~ f. We also investigated the impact of partitioning samples with a less restrictive ~~criteria~~ criterion based on the assumed mutation rate as described above. Similar to research in the comparison of power-law distributions [49], we ~~utilize~~ utilized the empirical cumulative densities ~~f~~ f using stat_ecdf and compare distributions using the Kolmogorov-Smirnov test with base R v3.6.3 [50]. We ~~plot~~ plotted the empirical distributions as smoothed kernel density distributions using the function stat_density contained in the R package ggplot2 v3.3.0 [51].

0.6.4 Sensitivity analyses

We investigated the results by performing a series of sensitivity analyses including statistical bootstrapping. Distance permutations between ~~worms pairs~~ pairs of worms included 100 iterations of sampling without replacement for the ~~distance vector~~the all-by-all pairwise geographic distances. Multi-infected host bootstrap subsampling included 100 iterations of choosing a single case worm at random for each host. In instances where only one case is observed per host, that case was consistently the representative. Each iteration was considered an independent distribution and analyzed as described above. In addition, we also tested the impact of relaxed similarity thresholds for defining matched identity of the molecular barcodes between pairs on the geographic clustering.

0.6.5 Analyzing the diversity of genetic samples by geography

~~Population worm cases (426) were ordered by ascending distance in reference to a single worm case, herein referred to as the index worm for each sample comparison. Cumulative barcode diversity scores were calculated for each worm in the study as the index case. Each worm case barcode that did not match the index case augmented the barcode diversity by one. When two worms were equidistant from the index case, the highest cumulative score was used as the barcode diversity for that distance metric.~~ A cumulative barcode diversity score was calculated for each worm with GPS coordinate data (426 worms). The number of unique barcodes was counted for an expanding radius around each worm until every other worm in the study was included in the radius. We also investigated the effect of opportunistic sampling on barcode geographic clustering by comparing the cumulative distance from ~~an index~~ each case to every other worm in the population. ~~From each index case, we expect~~ From each worm, we expected the number of samples to increase at variable rates depending on sample clusters. Deviations from an increase (i.e. a sustained flat line) between samples would indicate geographic breaks between sample clusters.

0.7 Analyzing sensitivity of additional loci outside of CO3, cytB, and ND3-5

Variants ~~were considered to be within loci if found within the gene ranges provided by PlasmoDB for each gene;~~ in the complete mitochondrial genome were grouped by whether the variant was in the CO3~~(~~, cytB, and ND3-5 loci ranges ('within loci') or in other regions of the genome ('outside of loci', including non-coding regions). Loci bounds were determined by the start and end positions of genes in the *Dracunculus medinensis* mitochondria genome version JN555591.1 instead of the amplicon alignment ranges due to the differences in sample processing and sequencing technology. The genomic ranges for the loci are $3,778$-$4,543$ ~~), cytB (~~for CO3, $2,619$-$3,720$ ~~), and ND3-5 (~~for cytB, and $12,550$-$14,467$ ~~). Note, these mitochondrial coordinates slightly differed from the ranges identified by target loci sequencing.~~ for ND3-5. Pairwise genetic similarity was calculated from barcodes created with all variants, variants within loci, or variants outside of loci~~as described in the previous section.~~ . For bootstrap subsampling tests, variants outside of loci ranges were randomly selected to contain the same number of variants found within loci to create a new barcode for genetic similarity comparisons. ~~Genetic similarity tests~~ Pairwise

genetic similarity comparisons for the different ~~regions~~ loci groups were robustly tested across two variant calling pipelines (GATK and bcftools mpileup, see ~~DNA alignment and variant calling~~0.3.2) with all variants and ~~singletons excluded.~~ singleton variants excluded. Smooth kernel density distributions were generated with ggplot2 v3.3.0 option "stat_density". A two sided Kolmogorov-Smirnov test was used to compare genetic similarity distributions of different loci classes with base R v3.6.3 [50].

Results

0.8 Genetic and epidemiological characteristics of Guinea worm cases.

Four hundred and fifty-nine worms (30 worms from humans hosts, 429 worms from dog hosts) collected from 2012-2018 were successfully sequenced (Fig 1A). Four hundred and twenty-six ~~of~~ successfully sequenced worms were matched to ~~case~~ GPS coordinates for each reported case. Worm cases mainly clustered along the Chari River (Fig 1A). Identical mitochondrial sequences were observed for cases across years (Fig 1B). Eighty six variants were identified in the CO3, cytB, and ND3-5 loci of 618, 606, and $1,961$ base pairs, respectively. The concatenation of the 86 variants resulted in 41 unique molecular barcodes (S1 Table).

Barcode accumulation curves suggest the 41 barcodes in this study ~~are not saturating~~ did not saturate the potential diversity of the unobserved population ~~given the proportion of low frequency barcodes in the observed population. We studied the discovery of new barcodes beyond the 41 observed with extrapolations up to 5000 samples using a~~ . We estimated the number of barcodes that may be uncovered with more sequencing. A standard negative binomial and ~~a statistical,~~ an empirical Bayes approach [52] ~~. The negative binomial procedure suggests~~ estimated we may observe 25-40 additional barcodes ~~and the empirical Bayes approach suggests we may observe up to 15 additional barcodes~~ for a sampled population size of 5000. Despite the differences between methods, both ~~indicate~~ indicated that modestly increasing the number of samples sequenced is not likely to capture the complete underlying genetic diversity. ~~Moreover, both methods indicate extrapolating to an even larger number of sequenced samples, 5000 or larger, may not be enough due to the~~ However, the extrapolation was hindered by the available data; it is unclear whether the abundance of low-frequency barcodes in the ~~sampled population. However, the extrapolation is hindered by the available data; see~~ currently sampled population are representative of

the population. See S1 Appendix for further methodological details and numerical investigations.

0.9 ~~Phylogenetic similarity between host species.~~

0.9 Molecular barcode similarity and persistence between host species.

~~There is not a consistent trend of interspecies transmission for barcodes shared across host species. Phylogenetic analysis of all available Guinea worm barcodes collected from humans and dogs identifies clades of repeated or highly similar barcodes. There is no clear signal of unilateral transmission between host species (S1 Fig~~ Twelve of the 41 identified barcodes ~~are~~ were shared between human and dog hosts. There were more unique dog barcodes driven by the high number of reported cases in dogs (Fig 1A). From the years included in this study it does not appear worms were shifting from one host species to the other (S1 FigA). Barcodes were likely to appear in ~~both species at the same time~~ dogs and humans in the same year (barcodes 2, 6, 8, 10, 36), dogs a year prior to humans (barcodes 5, 7, 13, 21), or in humans a year prior to dogs (barcodes 3, 4, 14). ~~There are more unique dog barcodes driven by the high number of reported cases in dogs (Fig 1A); however, barcodes found exclusively in one species do not form separate clades. The average nucleotide similarity (π) between human and dog barcodes ranged from 0.6-1.0, similar to within host nucleotide similarity ranges. Human samples are the sole genetic representative for 2012, 2013, and 2018 cases with 1, 2, and 11 samples. Only four barcodes from this set (7, 21, 40 and 41)were observed as a singular event.~~ In addition, DAPC of barcodes highlighted a challenge to discriminate between lineages of human and dog hosts suggesting they share a genetically similar Guinea worm parasite population (S1 FigB).

0.10 Genetically identical ~~worms~~ barcodes are geographically clustered.

0.10.1 Population level comparisons

~~Pairwise genetic similarity scores for all worms with GPS data available reveals genetically identical barcodes are~~ Genetically identical barcodes were spatially clustered in Chad. Identical barcodes ~~are~~ were within a median 18.6 kilometer range (standard deviation = 82.5 kilometers), and often within an approximately 50 kilometer

radius (Fig 2A). Non-identical barcodes were more evenly distributed within a median 222.2 kilometers (standard deviation = 157.5 kilometers), ~~however spread across a 400 kilometer radius,~~ with slight inclination to be clustered within 100 kilometers or 400 kilometers (Fig 2A). The spatial distributions between identical and non-identical barcode distributions ~~are~~ were statistically different (Kolmogorov–Smirnov test, p < 0.001). ~~Reclassifying worm pairs~~ Reclassifying pairs of worms with a single variant difference to account for potential ~~elevated~~ underestimated mutation rates or a sequencing ~~mutation~~ error as identical robustly replicated geographical clustering within a 50 kilometer radius (S2 Fig). Permuting the distance between ~~worm pairs~~ pairs of worms removed spatial clustering for identical worms (S3 Fig).

Geographic clustering of barcodes ~~is not due solely~~ was not due to the similarity of parasites within hosts that carry multiple infections. Sixty-five of the 245 hosts had multiple worm infections, with a median of 4 worms (range = 2-24) per multi-infected host (S4 Fig). Worms captured from the same host were not always genetically identical. The distribution of genetic similarity for worms ~~with~~ from the same host did not differ from worms with the same GPS coordinates from different hosts (S4 Fig). Bootstrap subsampling of a single worm from each host maintained spatial clustering with low variability (S5 Fig).

0.10.2 Individual barcode comparisons

Individual barcodes ~~show~~ showed variation in their geographical range (Fig 2B). For example in Fig 1C, cases with Barcode 2 ~~are~~ were found in the southeast mouth of the Chari River, while cases with Barcode 3 and 4 ~~are~~ were spread along the entire river. The geographic range for samples in each common barcode can be seen in S6 Fig. We replicated the smooth kernel density estimates for 10 of the 38 barcodes; note that three of the barcodes do not contain associated GPS locations for any sample. The geographic variability ~~is not dependent on~~ was not correlated to the total number of worms assigned to a barcode (S1 Table). Two of the barcodes seen in Fig 2B represented with pink and mint ~~are~~ were more similar to the null distribution and found in a broader geographic range in Chad.

The variation in geographical ranges ~~is~~ for barcode groups was not driven by a geographical isolation~~of cases sharing barcodes~~. For most worms, we ~~observe~~ observed a steep increase in unique barcodes up to 200 kilometers (Fig 3). Worms with a slow increase in the unique barcodes after 200 kilometers ~~correspond~~ corresponded to the human cases observed furthest east in Fig 1A. ~~These index worms follow~~ These worms

followed a similar steep increase once the radius includes samples along the Chari River. The plateau of diversity observed in most worms around 18-20 unique barcodes is an artifact of opportunistic sampling proximity (S7 Fig).

0.11 Expanding genetic markers can improve sensitivity for comparing worm populations.

The extraction protocol of Guinea worm from an infected host results in degraded parasite DNA and a microbial mixture that is problematic for standard extraction and untargeted sequencing protocols. This limitation has dampened large scale genetic studies of Guinea worm using additional markers on the mitochondrial and nuclear genome. It was unclear whether variants in other regions of the mitochondrial genome would significantly alter population worm relatedness to consider protocol development for other markers. To determine the value of including variants outside of the loci targeted in this study, we compared variants in other regions of the mitochondrial genome from an independent set of 19 worm samples with successful DNA extraction and shotgun sequencing [12]. ~~Variants were called across the mitochondrial genome from publicly available data for 19 worms from Chad [12].~~

Including mitochondrial variants outside of CO3, cytB, and ND3-5 loci ~~increased the discrimination between worm pairs in an independent population~~ changed the distribution of genetic similarity between pairs of worms(Fig 4). Forty-seven variants were found within ~~the~~ loci sampled in this study, and 129 variants outside of ~~the samples loci range~~loci. ~~A shift in the genetic similarity distribution in any direction suggests the new loci can alter the genetic relatedness of a sampled population. We observed a shift in the probability function mass from 0.55 similarity for within loci variants to a second mass at 0.80 for outside loci variants.~~ Variants within loci differentiated two peaks in the density estimates compared to all variants and variants outside of loci that differentiated three peaks in the density estimates. The distribution of genetic similarity between worms using the 47 loci variants ~~is~~ were statistically different than the distribution of genetic similarity using the 129 non-loci variants (Kolmogorov–Smirnov test, $p < 0.001$). The broader pairwise similarity distribution of outside loci ~~is~~ was robust to the number of variants in the pairwise calculation (Fig 4B). Distributions of randomly ~~subsampling~~ subsampled 47 ~~non-loci~~ outside loci variants compared to the distribution of 47 loci variants showed that in some instances the population was less genetically identical with major peaks shifted to the left, and maintaining a higher density ~~of similarity~~ in the 0.75-0.85 pairwise genetic

similarity range. However, because of the small sample size without accompanying GPS coordinates of this independent population, we ~~cannot~~ could not extrapolate the effect of barcode sets on the spatial links between identical and non-identical pairs.

Despite requiring a minimum of 10 reads for each variant at a position to ensure true variant signals, we additionally confirmed the change in pairwise genetic similarity excluding variants observed in one sample in the population. Excluding variants found in only one worm, we ~~drop~~ dropped from 176 variants to 135 variants for comparison across the mitochondrial genome. ~~The distribution of outside loci genetic similarity is still broadened in relation to within loci, but the effect is less pronounced (S8 Fig, Kolmogorov–Smirnov test p = 0.001). Distributions of randomly subsampled 35 non-loci variants compared to the distribution of 35 loci variants had a less pronounced decrease in sequence similarity compared to the inclusion of singletons, but confirmed a higher density of similarity in the 0.75-0.85 range.~~The shape of the pairwise distribution including variants identified outside of the CO3, cytB, and ND3-5 loci had three mass peaks compared to two mass peaks when only considering variants within the CO3, cytB, and ND3-5 loci (S8 Fig, Kolmogorov–Smirnov test p = 0.001). This robust trend also observed when including singleton variants showed the pairwise distance between pairs or worms was not driven by unique variants in the population. Variants were called with an orthogonal method to circumvent any issues with genotype calling on a small sample size in GATK. More variants across the mitochondrial genome ~~are~~ were identified calling with bcftools than GATK (185 versus 175 variants). Variants calls with bcftools replicated the different distributions for variants within loci and outside loci with a ~~secondary~~ new mass peak around 0.75 pairwise genetic similarity. (S9 Fig, Kolmogorov–Smirnov test, p = 0.001).

Discussion

To our knowledge, this is the largest retrospective study linking surveillance and genetic data to understand Guinea worm transmission in Chad to date, encompassing samples from 2012-2018, excluding 2014. Knowing the geographical range of transmission for genetically linked samples has the potential to be an important monitoring and evaluation tool for the campaign. We identified 41 unique molecular barcodes from the 459 Chadian worm ~~sequences~~samples. Among the 459 samples, 426 samples had associated GPS data. The analyses in this study provide quantitative ~~bounds~~ boundaries on the geographic range of most transmission within a median 18.6

kilometer radius, and often within an approximately 50 kilometer radius. These results suggest the most effective interventions should consider case sweeps, water monitoring, and abatement approximately 20 kilometers around a reported case to reduce the spread of genetically related parasites. The population distribution falling under 50 kilometers implies that extending these efforts from 20-50 kilometers would largely dampen the dispersion of genetically related worms, with diminishing returns for efforts greater than 50 kilometers from a reported case. However, worms linked by barcode identity are not geographically isolated populations. ~~Each sample is approximately~~ Samples are surrounded by worms representing five to ten different barcodes within a 50 kilometer radius (Fig 3), and individual barcodes can vary in their transmission range from three to 150 kilometers. ~~It is important to consider the genetic diversity of worms in an area when guiding geographically~~ Geographically imposed interventions from these results need to consider the dispersion range variation for cases identified in areas with co-occurring lineages.

The modeling results presented in this article are consistent with previous analyses, but also expand the scope of geographic and genetic relatedness. ~~The phylogenetic analysis of barcodes created from all 459 samples in this work~~ Overlapping barcodes of worms collected from human and dog hosts supports the earlier conclusion by Thiele et al. [10] that humans and dogs share a similar Guinea worm population in their analysis of 75 samples (S1 FigA). ~~There is not a clear temporal trend between host species and suggests~~ Barcodes did not consistently shift from one host species over time, suggesting a fluid transmission of *D. medinensis* between humans and dogs. Previous genetic analyses using spatial principal components analysis had identified a geographic trend of genetic relatedness down the Chari River in Chad [10, 44, 53]. The research in this article ~~has expanded the scope of~~ broadens that analysis by revealing that genetically identical and near-identical samples cluster geographically for multiple areas across Chad. (Fig 1C).

The findings of this work ~~are also aligned~~ also align with the known epidemiology of the disease and biology of the parasite. Recent surveillance efforts involving the collaring and GPS tracking of dogs in Chad ~~show~~ showed dogs visit water sources within a 10 kilometer range, with variation across study sites [54]. ~~In our study, the substantial mass of the genetic similarity distributions around 18.6 kilometers usually within~~ The substantial geographic distance distribution for genetically identical pairs of worms under 50 kilometers are broadly in agreement with the dog roaming range ~~including the observed~~ and variation by geography ~~; see~~ (Fig 2~~B for reference~~). Both of these research

efforts support the current epidemiological intuition and hypotheses about geographic connectivity of infections and the role of dogs as a reservoir [10, 45, 54]. ~~These analyses provide quantitative bounds on the geographic range of local transmission.~~

~~Identifying~~ The numerous sets of identical or nearly identical ~~genetic sequences~~ molecular barcodes among the 459 samples ~~is also expected~~was expected. We anticipated some barcode sets would be maintained throughout the few years represented in this study due to the parasite biology of a year-long prepatent period and the suspected low spontaneous mutation rate (see section 0.8). ~~Direct maternal mitochondrial inheritance ensures genetic markers on this region are informative of lineage, especially considering the small estimated spontaneous mutation rate or chance of a sequencing error (§0.8). While identifying identical or nearly identical molecular barcodes does not allow us to directly infer transmission events or ancestry, the persistence of barcodes across available years, coupled with the parasite biology, suggests a sustained population of worms related through transmission.~~ Even though the probability of having observed a direct transmission event in the available genetic samples is quite low (given the total number of reported cases), the molecular barcodes demonstrate distinct value even though the transmission network is only partially observed. The direct maternal inheritance of mitochondrial DNA and the persistence of barcodes across available years suggests a sustained population of worms are related through transmission. Taken together, these findings strongly suggest an epidemiological connection identified using the genetic data and can help inform the local epidemiology of Guinea worm in Chad.

There are several limitations to the analysis and modeling in this article. The samples that were sequenced and included in this analysis ~~, specifically for worms collected from dog hosts,~~ were retrospectively selected in order to span the geography of Chad and not through a systematic sampling frame. As mentioned, this ~~lead~~ led to an absence of 2014 samples in this study. We ~~have mitigated the challenges posed by constraints on the data and methodologies by evaluating the~~ evaluated the robustness of each conclusion ~~through a series of sensitivity analyses~~ with sensitivity analyses to address the challenges posed by data constraints and methodologies (Fig 1 and 2) ~~.~~ We demonstrated the geographic clustering results ~~are~~ were not sensitive to ~~the fact that many of the hosts were multiply infected with Guinea worm~~ dogs with multiple worm infections which could have artificially inflated the geographic proximity between ~~sample pairs~~ pairs of worms (S5 Fig).

In addition, the characteristics of the *Dracunculus medinensis* genome is not as

well-understood as ~~other~~ viruses, bacteria, or other parasites. ~~There does not exist a set of tailored phylogenetic or phylodynamic methodologies for Guinea worm parasites [10,55]. With either a comprehensive~~ Without knowledge of baseline genetic diversity from Chad, a known or the mutation rate for Guinea worm, ~~or both, we could~~ we could not more accurately assess the genetic similarity threshold to ~~cluster worms based on the mutation biology of the parasite~~group lineages. Despite these constraints, we showed that allowing for a single base pair difference ~~between worm pair barcodes~~ in barcodes between a pair of worms maintained spatial clustering ~~of identical and nearly identical barcodes relative to unrelated barcodes that have~~ relative to two or more ~~mutations between pairs in the population~~ base pair differences in barcodes between a pair of worms (S2 Fig). ~~However, a more resolved characterization between pairs of samples or barcodes is potentially~~ Lineage representation is likely limited by the use of three mitochondrial loci coupled with the small sample size relative to the number of observed and unobserved total cases from 2015-2017. ~~To test this hypothesis, we investigated~~ In an independent population of ~~genetic samples that had more~~ samples with complete coverage of the mitochondrial genome~~(§0.11). We were able to identify that more variants are observed~~ , we confirmed variants in other regions of the mitochondrial genome ~~(§0.11), which was confirmed~~ changed the distribution of population pairwise genetic relatedness (see section 0.11). This trend was replicated with a combination of bootstrapping and variant callers. Due to small sample sizes of available whole ~~mtDNA~~mitochondrial DNA, we were unable to conclude whether the extra variants ~~improve the genetic differentiation within barcode sets and refine the~~ refine geographic clustering. The substantial number of additional variants in the mitochondrial genome strongly suggests that access to more of the genome will ~~allow better between resolution of genetic similarity for Chadian~~ better resolve identical lineages for Guinea worm.

Despite these limitations, the modeling and analyses in this article have important implications for policy makers and elimination programs. From 2010-2019, there ~~has been~~ was a concurrent increase in both reported cases and surveillance efforts~~; a~~ . A complete characterization of the genetic diversity could help distinguish whether Guinea worm prevalence ~~is actually increasing or is~~ was increasing or a consequence of improved surveillance. ~~Although there is uncertainty of how genomic signatures are impacted by interventions and the potential temporal lags caused by the year-long life cycle, the~~ The continued appearance of genetically identical worms across years suggests genomic data is informative for understanding transmission, surveillance, and even

interventions. Monitoring the genetic landscape could provide programmatic evidence for the effectiveness of geographically localized interventions by observing the potential elimination of barcode lineages. Sustained barcodes are particularly useful in instances where case reports may be disrupted due to insecurity or inaccessibility.

A comprehensive bank of all genomic samples paired with geographic data would allow a broader set of analyses to help the elimination program~~for~~; for example, outbreak analysis, importation versus local circulation characterization, and potentially reveal unknown animal reservoirs. ~~With the available genetic data, we~~ We cannot provide programmatic guidance on a specific number of samples that should be collected and sequenced to capture the genetic diversity in Chad due to the high diversity of the *currently* sampled population (S1 Appendix). ~~Sequencing more historical samples could either decrease genetic diversity and orphaned pairs or support the high diversity of the population . The former would suggest a subset of samples could be sequenced to characterize Guinea worm diversity, while the latter would require sequencing a majority or all of the identified worms~~If a subset of historical samples share barcodes with the currently sampled population we would likely be powered to define a sample size for genetic surveillance. Conversely, more unique barcodes in historical samples would support the need to sequence all available worm samples due to the high diversity of the population. ~~Given current surveillance protocols , samples of the parasite are already collected~~ Current surveillance protocols already collect emerging worms in addition to a standardized survey for all reported cases. ~~Thus, sequencing a~~ Given the reported case counts and access to high-throughput sequencing technologies, it is tractable to sequence a large subset or all of the available retrospective and prospective Guinea worm samples~~is feasible with respect to the availability of genetic material, an established sequencing protocol, and access to efficient high-throughput sequencing technologies. The confirmed improvement in genetic resolution~~ .

The confirmed change in population pairwise genetic similarity across the whole mitochondrial genome suggests ~~switching sequencing technologies for the proposed scale up of genetic sequencing of worms~~ expanding the marker set is an important future research direction. ~~More broadly, coupling these findings with innovations on~~ An expanded marker set would complement innovations constructing phylogenies from whole genome sequencing of the Guinea worm larvae [55] and microsatellites of the worm nuclear genome [10]~~, a~~. A holistic program of sequencing ~~technologies~~ strategies and analytic methodologies will help translate research insights into programmatic input for the elimination of Guinea worm in Chad. Furthermore, additional analyses of the

full range of epidemiological data collected by the CGWEP alongside linked genomic data are warranted, and may further elucidate transmission dynamics in Chad. The parasite genome has the potential to be an integral tool for the end-game strategy in Chad and beyond.

Acknowledgments

We would like to thank Dr. Fernando Torres for his helpful discussions regarding Guinea worm eradication efforts and Dr. Albert Lee for modeling discussions. This work would not be possible without the field and surveillance teams of the national Guinea worm eradication program in Chad. Thank you for the efforts of all involved to collect the epidemiological data and samples.

Funding statement

~~JVR, GCC, HL, and JLP would like to thank Bill and Melinda Gates for their active support of~~ This publication is based on research funded in part by the Bill & Melinda Gates Foundation, including but not limited to models and data analysis performed by the Institute for Disease Modeling ~~and their sponsorship through the Global Good Fund.~~ at the Bill & Melinda Gates Foundation by JVR, NL, HL, GCC, JLP. JAC was supported by funding from The Carter Center and Wellcome, via their core support for the Wellcome Sanger Institute (grant WT206194). The funders had no role in study design, data collection and analysis, decision to publish, or preparation of the manuscript.

Data availability

Genomic sequences are available on Genbank as MW257241 - MW257699 for CO3, MW257700 - MW258158 for cytB, and MW258159 - MW258617 for ND3-5 loci. The epidemiological data containing the case coordinates must be requested from and approved by the Chad Ministry of Public Health (contact via Tchonfienet Moundai, tchomcalvin@yahoo.fr). Analysis code can be accessed at https://github.com/InstituteforDiseaseModeling/GWSpatialGenetics.

Disclaimer

The findings and conclusions in this report are those of the author(s) and do not necessarily represent the official position of the Centers for Disease Control and Prevention/the Agency for Toxic Substances and Disease Registry.

References

1. Biswas G, Sankara DP, Agua-Agum J, Maiga A. Dracunculiasis (guinea worm disease): eradication without a drug or a vaccine. Philosophical Transactions of the Royal Society B: Biological Sciences. 2013;368(1623):20120146.

2. Hopkins DR, Weiss AJ, Roy SL, Zingeser J, Guagliardo SAJ. Progress Toward Global Eradication of Dracunculiasis—January 2018–June 2019. Morbidity and Mortality Weekly Report. 2019;68(43):979.

3. World Health Organization: Dracunculiasis eradication;. https://www.who.int/dracunculiasis/certification/en/.

4. Sreenivasan N, Weiss A, Djiatsa JP, Toe F, Djimadoumaji N, Ayers T, et al. Recurrence of Guinea worm disease in Chad after a 10-year absence: risk factors for human cases identified in 2010–2011. The American Journal of Tropical Medicine and Hygiene. 2017;97(2):575–582.

5. Center for Disease Control and Prevention: Morbidity and Mortality Weekly Report. Progress Toward Global Eradication of Dracunculiasis- January 2018 -June 2019;. https://www.cdc.gov/mmwr/volumes/68/wr/mm6843a5.htm.

6. Callaway E. Dogs thwart effort to eradicate Guinea worm. Nature News. 2016;529(7584):10.

7. Eberhard ML, Cleveland CA, Zirimwabagabo H, Yabsley MJ, Ouakou PT, Ruiz-Tiben E. Guinea worm (Dracunculus medinensis) infection in a wild-caught frog, Chad. Emerging Infectious Diseases. 2016;22(11):1961.

8. Molyneux D, Sankara DP. Guinea worm eradication: Progress and challenges—should we beware of the dog? PLoS Neglected Tropical Diseases. 2017;11(4).

9. Cleveland CA, Eberhard ML, Thompson AT, Smith SJ, Zirimwabagabo H, Bringolf R, et al. Possible role of fish as transport hosts for Dracunculus spp. larvae. Emerging Infectious Diseases. 2017;23(9):1590.

10. Thiele EA, Eberhard ML, Cotton JA, Durrant C, Berg J, Hamm K, et al. Population genetic analysis of Chadian Guinea worms reveals that human and non-human hosts share common parasite populations. PLOS Neglected Tropical Diseases. 2018;12(10):e0006747.

11. The Carter Center and the Center for Disease Control and Prevention: Guinea worm Wrap-Up, #267—April 7 2020;. https://www.cartercenter.org/resources/pdfs/news/health_publications/guinea_worm/wrap-up/267.pdf.

12. Durrant C, Thiele EA, Holroyd N, Doyle SR, Sallé G, Tracey A, et al. Population genomic evidence that human and animal infections in Africa come from the same populations of Dracunculus medinensis. PLOS Neglected Tropical Diseases. 2020;14(11):e0008623.

13. Garrett KB, Box EK, Cleveland CA, Majewska AA, Yabsley MJ. Dogs and the classic route of Guinea Worm transmission: an evaluation of copepod ingestion. Scientific Reports. 2020;10(1):1–5.

14. Pallansch MA, Sandhu HS. The eradication of polio—progress and challenges. New England Journal of Medicine. 2006;355(24):2508–2511.

15. Cowger TL, Burns CC, Sharif S, Gary Jr HE, Iber J, Henderson E, et al. The role of supplementary environmental surveillance to complement acute flaccid paralysis surveillance for wild poliovirus in Pakistan–2011–2013. PLOS ONE. 2017;12(7).

16. Famulare M, Hu H. Extracting transmission networks from phylogeographic data for epidemic and endemic diseases: Ebola virus in Sierra Leone, 2009 H1N1 pandemic influenza and polio in Nigeria. Int Health. 2015;7(2):130–138.

17. Dalmat R, Naughton B, Kwan-Gett TS, Slyker J, Stuckey EM. Use cases for genetic epidemiology in malaria elimination. Malaria Journal. 2019;18(1):163.

18. Tinbergen J. Shaping the world economy; suggestions for an international economic policy. vol. 46. Twentieth Century Fund, New York; 1962.

19. Bayati BS. Fractional diffusion-reaction stochastic simulations. The Journal of Chemical Physics. 2013;138(10):104117.

20. Brockmann D, Hufnagel L, Geisel T. The scaling laws of human travel. Nature. 2006;439(7075):462–465.

21. Riley S. Large-Scale Spatial-Transmission Models of Infectious Disease. Science. 2007;316(5829):1298–1301. doi:10.1126/science.1134695.

22. Balcan D, Colizza V, Gonçalves B, Hu H, Ramasco JJ, Vespignani A. Multiscale mobility networks and the spatial spreading of infectious diseases. Proc Natl Acad Sci USA. 2009;106(51):21484–21489. doi:10.1073/pnas.0906910106.

23. Yingcun X, Bjornstad ON, Grenfell BT. Measles Metapopulation Dynamics: A Gravity Model for Epidemiological Coupling and Dynamics. Am Nat. 2004;164(2):267–281. doi:10.1086/422341.

24. Jandarov R, Haran M, Bjornstad O, Grenfell B. Emulating a gravity model to infer the spatiotemporal dynamics of an infectious disease. J R Stat Soc Ser C Appl Stat. 2014;63(3):423–444. doi:10.1111/rssc.12042.

25. Viswanathan GM. Ecology: Fish in Levy-flight foraging. Nature. 2010;465:1018–1019.

26. Humphries NE, Weimerskirch H, Queiroz N, Southall EJ, Sims DW. Foraging success of biological Lévy flights recorded in situ. Proc Natl Acad Sci USA. 2012;109(19):7169–7174. doi:10.1073/pnas.1121201109.

27. Lemey P, Rambaut A, Bedford T, Faria N, Bielejec F, Baele G, et al. Unifying Viral Genetics and Human Transportation Data to Predict the Global Transmission Dynamics of Human Influenza H3N2. PLOS Pathogens. 2014;10(2):1–10. doi:10.1371/journal.ppat.1003932.

28. Backer JA, Wallinga J. Spatiotemporal analysis of the 2014 Ebola epidemic in West Africa. PLOS Computational Biology. 2016;12(12):1–17.

29. Klepac P, Metcalf CJE, McLean AR, Hampson K. Towards the endgame and beyond: complexities and challenges for the elimination of infectious diseases; 2013.

30. Gustafson KB, Proctor JL. Identifying spatio-temporal dynamics of Ebola in Sierra Leone using virus genomes. Journal of the Royal Society Interface. 2017;14(136):20170583.

31. Didelot X, Fraser C, Gardy J, Colijn C. Genomic Infectious Disease Epidemiology in Partially Sampled and Ongoing Outbreaks. Mol Biol Evol. 2017;34(4):997. doi:10.1093/molbev/msw275.

32. Rasmussen DA, Ratmann O, Koelle K. Inference for Nonlinear Epidemiological Models Using Genealogies and Time Series. PLOS Computational Biology. 2011;7(8):1–11. doi:10.1371/journal.pcbi.1002136.

33. Ypma RJF, van Ballegooijen WM, Wallinga J. Relating Phylogenetic Trees to Transmission Trees of Infectious Disease Outbreaks. Genetics. 2013;doi:10.1534/genetics.113.154856.

34. Rasmussen DA, Boni MF, Koelle K. Reconciling Phylodynamics with Epidemiology: The Case of Dengue Virus in Southern Vietnam. Molecular Biology and Evolution. 2013;31(2):258.

35. Rasmussen DA, Volz EM, Koelle K. Phylodynamic Inference for Structured Epidemiological Models. PLOS Computational Biology. 2014;10(4):1–16.

36. Jombart T, Cori A, Didelot X, Cauchemez S, Fraser C, Ferguson N. Bayesian Reconstruction of Disease Outbreaks by Combining Epidemiologic and Genomic Data. PLOS Computational Biology. 2014;10(1):1–14. doi:10.1371/journal.pcbi.1003457.

37. World Helath Organizaton: Technical consultation on the role of parasite and anopheline genetics in malaria surveillance; 2019. https://www.who.int/malaria/mpac/mpac-october2019-session7-report-consultation-on-genomics.pdf.

38. Li H, Durbin R. Fast and accurate short read alignment with Burrows–Wheeler transform. Bioinformatics. 2009;25(14):1754–1760. doi:10.1093/bioinformatics/btp324.

39. Van der Auwera GA, Carneiro MO, Hartl C, Poplin R, del Angel G, Levy-Moonshine A, et al. From FastQ Data to High-Confidence Variant Calls: The Genome Analysis Toolkit Best Practices Pipeline. Current Protocols in Bioinformatics. 2013;43(1):11.10.1–11.10.33. doi:10.1002/0471250953.bi1110s43.

40. Li H. A statistical framework for SNP calling, mutation discovery, association mapping and population genetical parameter estimation from sequencing data. Bioinformatics. 2011;27(21):2987–2993. doi:10.1093/bioinformatics/btr509.

41. Jombart T, Devillard S, Balloux F. Discriminant analysis of principal components: a new method for the analysis of genetically structured populations. BMC Genetics. 2010;11(1):94. doi:10.1186/1471-2156-11-94.

42. Jombart T. adegenet: a R package for the multivariate analysis of genetic markers. Bioinformatics. 2008;24:1403–1405. doi:10.1093/bioinformatics/btn129.

43. Jombart T, Ahmed I. adegenet 1.3-1: new tools for the analysis of genome-wide SNP data. Bioinformatics. 2011;doi:10.1093/bioinformatics/btr521.

44. Guagliardo SAJ, Roy SL, Ruiz-Tiben E, Zirimwabagabo H, Romero M, Chop E, et al. Guinea worm in domestic dogs in Chad: A description and analysis of surveillance data. PLOS Neglected Tropical Diseases. 2020;14(5):1–17. doi:10.1371/journal.pntd.0008207.

45. Eberhard ML, Ruiz-Tiben E, Hopkins DR, Farrell C, Toe F, Weiss A, et al. The peculiar epidemiology of dracunculiasis in Chad. The American Journal of Tropical Medicine and Hygiene. 2014;90(1):61–70.

46. Denver DR, Morris K, Lynch M, Vassilieva LL, Thomas WK. High Direct Estimate of the Mutation Rate in the Mitochondrial Genome of Caenorhabditis elegans. Science. 2000;289(5488):2342–2344. doi:10.1126/science.289.5488.2342.

47. Hamming RW. Error Detecting and Error Correcting Codes. Bell System Technical Journal. 1950;29(2):147–160. doi:10.1002/j.1538-7305.1950.tb00463.x.

48. Hijmans RJ. geosphere: Spherical Trigonometry; 2019. Available from: `https://CRAN.R-project.org/package=geosphere`.

49. Clauset A, Shalizi CR, Newman MEJ. Power-Law Distributions in Empirical Data. SIAM Review. 2009;51(4):661–703.

50. Team RC. R: A Language and Environment for Statistical Computing; 2020. Available from: `https://www.R-project.org/`.

51. Wickham H. ggplot2: Elegant Graphics for Data Analysis. Springer-Verlag New York; 2016. Available from: `https://ggplot2.tidyverse.org`.

52. Efron B. Large-scale inference: empirical Bayes methods for estimation, testing, and prediction. vol. 1. Cambridge University Press; 2012.

53. Eberhard ML, Ruiz-Tiben E, Hopkins DR, Farrell C, Toe F, Weiss A, et al. The Peculiar Epidemiology of Dracunculiasis in Chad. The American Journal of Tropical Medicine and Hygiene. 2014;90(1):61–70. doi:10.4269/ajtmh.13-0554.

54. McDonald RA, Wilson-Aggarwal JK, Swan GJ, Goodwin CE, Moundai T, Sankara D, et al. Ecology of domestic dogs Canis familiaris as an emerging reservoir of Guinea worm Dracunculus medinensis infection. PLOS Neglected Tropical Diseases. 2020;14(4):e0008170.

55. Cotton JA, Berriman M, Dalén L, Barnes I. Eradication genomics—lessons for parasite control. Science. 2018;361(6398):130–131.

Fig 1. Characteristics of genetically characterized Guinea worm cases in Chad from 2012-2018**.** A.) Number of cases per host species collected from 2012-2018, with GPS matched sample distributions across the Chari River. B.) The number of GPS matched samples that belong to barcode sets. Note, not all barcode colors are ~~show~~ shown in the left figure. Barcodes with less than 10 samples in the population are colored in light gray[illegible]r visual clarity. C.) Samples belonging to barcode sets 2, 3 and 4 are shown in their respective ranges. Smoothed kernel densities for the three barcode sets show the mass of the distributions correlate with the spatial connectedness of samples.

Fig 2. Distance density estimates of pairwise similarity for identical and non-identical barcodes. A.) Pairwise comparisons of identical barcodes in black ($n=10,695$) to a null of non-identical barcodes in gray ($n=79,830$). This ~~distrubtion~~ distribution at 400 kilometers is the max distance between sampled cases along the Chari River. This enrichment of ~~pirwise~~ pairwise sample distance around 400 kilometers should not be considered the transmission ~~upperbound~~upper bound. B.) The number of comparisons for each barcode are the unique pairs of all worms sharing that barcode identifier. The null distribution in gray of non-identical barcodes is subset to only include comparisons where one of the common gray must be found in the pair ($n = 56,913$).

Fig 3. ~~Opportunistic sampling does not drive spatial connectedness of samples~~Opportunistic sampling does not drive apparent spatial connectedness of samples. ~~Rarefaction~~ Accumulation curves of ~~index~~ worm cases on geographical proximity of barcode diversity shows a consistent addition of diversity in quantitatively identified transmission ranges of barcodes sets (<50 kilometers). The left figure shows all ~~rarefaction analyses~~ accumulation curves for each worm case, and the right figure is separated by the barcode set number (1-10 for common barcodes, other for barcodes found in less than 10 samples). The numbers in the lower right corner are the number of worms in each barcode set.

Fig 4. Distribution of all-by-all pairwise genetic similarity of variants within or outside of currently sampled loci. Within loci variants were determined with CO3, cytB, and ND3-5 genomic ranges ~~provided by PlasmoDB~~specified in the mitochondrial reference genome. Smoothed density kernel distributions are overlayed on pairwise genetic ~~similairty~~ similarity distribution histograms for the different genomic regions.

Supporting information

S1 Table. Barcode counts for sequenced samples. GPS columns indicate the number of samples for each barcode used in geospatial analyses by species. Columns starting with 'All' indicates the number of worms assigned to each barcode, including samples that were not linked to GPS data for each host species. Temporal presence/absence of barcodes can be tracked in S1 FigA per species.

S1 Fig. ~~**Phylogenetic comparison of worm barcodes identified per species.** This graph demonstrates that barcodes were shared among humans and dogs alike, and were often present over the course of multiple years. Tree tip labels represent a single barcode per species. Refer to S1 Table for sample counts of each barcode by species. Barcode identifying numbers are represented more than once if observed in both species. A blue filled cell next to each barcode is indicative that barcode was represented at least once in the respective year.~~ **Molecular barcode comparison of worm barcodes identified per species.**
A. The presence of different barcodes per host by year. A blue filled cell next to each barcode is indicative that barcode was represented at least once in the respective year. Refer to S1 Table for sample counts of each barcode by species. B. Discriminant analysis of principal components on the molecular barcodes by host. Barcodes found in samples from humans only were grouped with barcodes found in samples from humans and dogs to create more even groups with barcodes found only in samples from dogs (humans only = 5 barcodes, both = 12, dogs only = 24). The overlap of discriminant distributions predicted with five principal components suggests molecular barcodes do not differ by host.

S2 Fig. Spatiotemporal modeling of barcode relatedness collapsed by a single base pair difference. Number of pairwise comparisons for identical barcodes $= 13,967$, for non-identical barcodes $= 76,558$.

S3 Fig. Permutation of distance between cases on barcode relatedness densities. The x-axis is the permuted distance between ~~worm~~ pairs of worms and the y-axis represents the density for population genetic similarity scores for all worms (n=426). Each line represents worm pair distance permutation (n=100) for the population. The lines for identical and non-identical barcodes are consistent between permutations and overlap.

S4 Fig. Barcode identity within hosts and genetic similarity distribution by shared location.
A. Each multi-infected host with the number of worms pertaining to each barcode set. "Not common" refers to barcodes found in less than 10 samples in the population for visual clarity.
B. Distributions of genetic similarity between worms with the same reported GPS coordinates, colored by whether the pair is obtained from the same or different hosts.

S5 Fig. Host subsampling on barcode relatedness densities. The x-axis is the distance between ~~worm~~ pairs of worms and the y-axis represents the density for population genetic similarity scores with one worm per host (n = 282). Each line represents a bootstrap (n=100) of a single worm per host.

S6 Fig. ~~**Location of common barcodes in Southern Chad.**~~ **Location of common and not common barcodes in Southern Chad.** Samples are colored by their barcode identifier, and shapes represent the host species. Barcodes 3, 4, and 10 appear have ~~similiar~~ similar spatial distributions. Barcodes 2 and 5 have similar spatial distributions and are highly focal. ~~some~~ Some barcodes span a very large geographic area, which could suggest they are ancestral sequences that have diffused over time or are transmitting due to human behaviors.

S7 Fig. Geographic distance between cases for each worm. The geographic distance between worms relative to a single worm were organized in ascending order. The x axis is the cumulative worm ~~index~~ count by distance and the y axis is the cumulative distance from each index worm. Spans of a flattened curve are indicative of geographic stretches that do not contain any samples and support the lack of barcode diversity observed in ~~cerain~~ certain geographic distances in Fig 3.

S8 Fig. Distribution of pairwise similarity using GATK variants found in greater than one worm. The x-axis is the measured genetic similarity for variants within current loci (n=35) and extending to the rest of the ~~mitochondria~~ mitochondrial genome (n=100), and y-axis is the number of pairwise comparisons (19*19 = 361). Filled regions show the smoothed density estimates for histograms.

S9 Fig. Orthogonal validation of population pairwise similarity using variants identified by bcftools mpileup. The x-axis is the measured genetic similarity for variants within current loci (n=47) and extending to the rest of the ~~mitochondria~~ mitochondrial genome (n=138), and y-axis is the number of pairwise comparisons (19*19 = 361). Filled regions show the smoothed density estimates for histograms.
