# Supplementary material for: Linked surveillance and genetic data uncovers programmatically relevant geographic scale of Guinea worm transmission in Chad
Source: PLoS Negl Trop Dis. 2021 Jul 26;15(7):e0009609. doi: 10.1371/journal.pntd.0009609 (PMC8341693; doi:10.1371/journal.pntd.0009609)
Supplement: Attachment — Submitted filename: PNTD-D-20-01771_R2_reviewer.pdf [file pntd.0009609.s018.pdf]

PLOS Neglected Tropical Diseases

Linked surveillance and genetic data uncovers programmatically relevant geographic scale of Guinea worm transmission in Chad

--Manuscript Draft--

**Manuscript Number:**	PNTD-D-20-01771R2
**Full Title:**	Linked surveillance and genetic data uncovers programmatically relevant geographic scale of Guinea worm transmission in Chad
**Short Title:**	Surveillance & genetic data uncovers geographic scale of Guinea worm transmission in Chad
**Article Type:**	Research Article
**Keywords:**	phylodynamics, genetic surveillance, transmission dynamics, mitochondria, surveillance, programmatic recommendations
**Abstract:**	Background<br>Guinea worm (Dracunculus medinensis) was detected in Chad in 2010 after a supposed ten year absence, posing a challenge to the global eradication effort. Initiation of a village-based surveillance system in 2012 revealed a substantial number of dogs infected with Guinea worm, raising questions about paratenic hosts and cross-species transmission.<br>Methodology/Principal Findings<br>We coupled genomic and surveillance data from 2012-2018 cases to investigate the modes of transmission between dog and human hosts and the geographic connectivity of worms. Eighty-six variants across three loci in the mitochondrial genome identified 41 genetically distinct worm genotypes. Spatiotemporal modeling revealed worms with the same genotype ('genetically identical') were within a median range of 18.6 kilometers of each other, but largely within approximately 50 kilometers. Genetically identical worms varied in their degree of spatial clustering, suggesting there may be different factors that favor or constrain transmission. Each worm was surrounded by five to ten genetically distinct worms within a 50 kilometer radius. As expected, we observed a change in the genetic similarity distribution between pairs using markers across the mitochondrial genome of worms in an independent population.<br>Conclusions/Significance<br>In the largest study linking genetic and surveillance data to date of Guinea worm cases in Chad, we show genetic similarity and modeling can contribute to understanding local transmission. The co-occurrence of genetically distinct worms in quantitatively identified transmission ranges highlights the necessity for genomic tools to link cases. The improved discrimination between worm pairs from variants identified across the complete mitochondrial genome indicates expanding genomic markers could link cases at a finer scale. These results suggest that scaling up genomic surveillance for Guinea worm may provide additional value for programmatic decision-making critical for monitoring cases and intervention efficacy to achieve elimination.
**Additional Information:**	
**Question**	**Response**
**Financial Disclosure**<br><br>Enter a financial disclosure statement that describes the sources of funding for the work included in this submission. Review the submission guidelines for detailed requirements. View published research articles from *PLOS NTDs* for specific examples.<br><br>This statement is required for submission	This publication is based on research funded in part by the Bill & Melinda Gates Foundation, including but not limited to models and data analysis performed by the Institute for Disease Modeling at the Bill & Melinda Gates Foundation by JVR, NL, HL, GCC, JLP.<br><br>JAC was supported by funding from The Carter Center and Wellcome, via their core support for the Wellcome Sanger Institute (grant WT206194).<br><br>The funders had no role in study design, data collection and analysis, decision to publish, or preparation of the manuscript.

Powered by Editorial Manager® and ProduXion Manager® from Aries Systems Corporation

and **will appear in the published article** if the submission is accepted. Please make sure it is accurate.<br><br>**Unfunded studies**<br><br>Enter: *The author(s) received no specific funding for this work.*<br><br>**Funded studies**<br><br>Enter a statement with the following details:<br>• Initials of the authors who received each award<br>• Grant numbers awarded to each author<br>• The full name of each funder<br>• URL of each funder website<br>• Did the sponsors or funders play any role in the study design, data collection and analysis, decision to publish, or preparation of the manuscript?<br>• **NO** - Include this sentence at the end of your statement: *The funders had no role in study design, data collection and analysis, decision to publish, or preparation of the manuscript.*<br>• **YES** - Specify the role(s) played.<br><br>* typeset	
**Competing Interests**<br><br>Use the instructions below to enter a competing interest statement for this submission. On behalf of all authors, disclose any competing interests that could be perceived to bias this work—acknowledging all financial support and any other relevant financial or non-financial competing interests.<br><br>This statement **will appear in the published article** if the submission is accepted. Please make sure it is	The authors have declared that no competing interests exist.

Powered by Editorial Manager® and ProduXion Manager® from Aries Systems Corporation

accurate. View published research articles from *PLOS NTDs* for specific examples.<br><br>**NO authors have competing interests**<br><br>Enter: *The authors have declared that no competing interests exist.*<br><br>**Authors with competing interests**<br><br>Enter competing interest details beginning with this statement:<br><br>*I have read the journal's policy and the authors of this manuscript have the following competing interests: [insert competing interests here]*<br><br>* typeset<br><br>This statement is required for submission and **will appear in the published article** if the submission is accepted. Please make sure it is accurate and that any funding sources listed in your Funding Information later in the submission form are also declared in your Financial Disclosure statement.	
**Data Availability**<br><br>Authors are required to make all data underlying the findings described fully available, without restriction, and from the time of publication. PLOS allows rare	No - some restrictions will apply

exceptions to address legal and ethical concerns. See the PLOS Data Policy and FAQ for detailed information.<br><br>A Data Availability Statement describing where the data can be found is required at submission. Your answers to this question constitute the Data Availability Statement and **will be published in the article**, if accepted.<br><br>**Important:** Stating 'data available on request from the author' is not sufficient. If your data are only available upon request, select 'No' for the first question and explain your exceptional situation in the text box.<br><br>Do the authors confirm that all data underlying the findings described in their manuscript are fully available without restriction?	
**Describe where the data may be found in full sentences. If you are copying our sample text, replace any instances of XXX with the appropriate details.**<br><br>• If the data are **held or will be held in a public repository**, include URLs, accession numbers or DOIs. If this information will only be available after acceptance, indicate this by ticking the box below. For example: *All XXX files are available from the XXX database (accession number(s) XXX, XXX.)*.<br>• If the data are all contained **within the manuscript and/or Supporting Information files**, enter the following: *All relevant data are within the manuscript and its Supporting Information files.*<br>• If neither of these applies but you are	Genomic sequences are available on Genbank as MW257241 - MW257699 for CO3, MW257700 - MW258158 for cytB, and MW258159 - MW258617 for ND3-5 loci. Analysis code can be accessed at https://github.com/InstituteforDiseaseModeling/GWSpatialGenetics. The epidemiological data containing the case coordinates must be requested from and approved by the Chad Ministry of Public Health (contact via Tchonfienet Moundai, tchomcalvin@yahoo.fr)

Powered by Editorial Manager® and ProduXion Manager® from Aries Systems Corporation

able to provide **details of access elsewhere**, with or without limitations, please do so. For example:<br><br>*Data cannot be shared publicly because of [XXX]. Data are available from the XXX Institutional Data Access / Ethics Committee (contact via XXX) for researchers who meet the criteria for access to confidential data.*<br><br>*The data underlying the results presented in the study are available from (include the name of the third party and contact information or URL).*<br>• This text is appropriate if the data are owned by a third party and authors do not have permission to share the data.<br><br>* typeset	
Additional data availability information:	Tick here if the URLs/accession numbers/DOIs will be available only after acceptance of the manuscript for publication so that we can ensure their inclusion before publication.

Manuscript - Revision 2
Click here to access/download;Manuscript;GuineaWormPhylodynamics_2021

Linked surveillance and genetic data uncovers programmatically relevant geographic scale of Guinea worm transmission in Chad

Jessica V. Ribado[1], Nancy Li[1], Elizabeth Thiele[2], Hil Lyons[1], James A. Cotton[3], Adam Weiss[4], Philippe Tchindebet Ouakou[5], Tchonfienet Moundai[5], Hubert Zirimwabagabo[4], Sarah Anne J. Guagliardo[4,6], Guillaume Chabot-Couture[1], Joshua L. Proctor[1*]

1 Institute for Disease Modeling, Global Health Division of the Bill and Melinda Gates Foundation, Seattle, WA, USA
2 Vassar College, Poughkeepsie, NY, USA
3 Wellcome Sanger Institute, Hinxton, Cambridgeshire, UK
4 The Carter Center, Atlanta, GA, USA
5 National Guinea Worm Eradication Program, Ministry of Public Health, N'Djamena, Chad
6 Centers for Disease Control and Prevention, Atlanta, GA, USA

* Corresponding author
Email: jproctor@idmod.org

Abstract

Background

Guinea worm (*Dracunculus medinensis*) was detected in Chad in 2010 after a supposed ten-year absence, posing a challenge to the global eradication effort. Initiation of a village-based surveillance system in 2012 revealed a substantial number of dogs infected with Guinea worm, raising questions about paratenic hosts and cross-species transmission.

Methodology/Principal Findings

We coupled genomic and surveillance data from 2012-2018 cases to investigate the modes of transmission between dog and human hosts and the geographic connectivity of worms. Eighty-six variants across three loci in the mitochondrial genome identified 41 genetically distinct worm genotypes. Spatiotemporal modeling revealed worms with the same genotype ('genetically identical') were within a median range of 18.6 kilometers of each other, but largely within approximately 50 kilometers. Genetically identical worms varied in their degree of spatial clustering, suggesting there may be different factors that favor or constrain transmission. Each worm was surrounded by five to ten genetically distinct worms within a 50 kilometer radius. As expected, we observed a change in the genetic similarity distribution between pairs using markers across the mitochondrial genome of worms in an independent population.

Conclusions/Significance

In the largest study linking genetic and surveillance data to date of Guinea worm cases in Chad, we show genetic identity and modeling can contribute to understanding local transmission. The co-occurrence of genetically non-identical worms in quantitatively identified transmission ranges highlights the necessity for genomic tools to link cases. The improved discrimination between pairs of worms from variants identified across the complete mitochondrial genome confirms that expanding the number of genomic markers could link cases at a finer scale. These results suggest that scaling up genomic surveillance for Guinea worm may provide additional value for programmatic decision-making critical for monitoring cases and intervention efficacy to achieve elimination.

Author summary

The global eradication effort for Guinea worm disease has dramatically decreased the global burden of the disease and enabled 187 countries to be certified by the World Health Organization to be free of endemic transmission. However, several countries continue to have endemic transmission; in Chad, a long absence of reported cases was interrupted with the identification of Guinea worm cases. Surveillance and intervention efforts have substantially scaled up in Chad. Here, we study the value of increasing genomic surveillance as a tool for programmatic evaluation of surveillance and intervention efforts in Chad. Linking surveillance and genomic samples, parsimonious spatial models help reveal a consistent geographic clustering of similar genetic sequences across Chad. We also demonstrate that expanding the sequencing can offer better resolution for distinguishing Guinea worm samples. In this retrospective study, we found evidence that scaling up genomic surveillance can be an important monitoring and evaluation tool for the eradication program in Chad.

Introduction

The eradication campaign for dracunculiasis, Guinea worm disease, has made substantial progress since the first set of World Health Assembly resolutions aimed at elimination efforts passed during the International Drinking Water Supply and Sanitation Decade (1981-1990) [1]. The Guinea worm eradication campaign has decreased the global burden of disease by more than 99% [2], enabled the World Health Organization (WHO) to certify 187 countries as free from endemic dracunculiasis transmission [3], and decreased the significant economic loss associated with Dracunculiasis in rural settings [1]. Despite the substantial progress, endemic transmission has persisted in Angola, Chad, Ethiopia, Mali, and South Sudan [2]. In Chad, a decade-long absence of reported cases was interrupted with a detected resurgence of Guinea worm cases in 2010 [4]; moreover, human cases continue to be reported [5] with a growing understanding of the role of animal reservoirs in transmission [6–10]. The stalled progress in Chad has led to a substantial increase in surveillance and programmatic efforts [11] as well as investments in research and novel interventions [12, 13].

Genetic analysis is an important tool for the control of other pathogens. In the case of polio eradication, for example, these tools have been used to detect silent transmission across geographic areas and thus inform programmatic decision-making [14–16]. The inclusion of strain differentiation techniques into programmatic decision-making for poliomyletis is feasible due to the continual mutation events in the virus genome, a growing global library of samples and isolates, and a mature set of mathematical and statistical methodologies to track specific lineages. For parasites, such as Guinea worm, well-established phlyogenetic methodologies to infer high-fidelity ancestry and lineages do not yet exist [10, 17]. Nonetheless, genetic data collected from Guinea worms in Chad has already revealed research and programmatically relevant insights: human and dog hosts share a common genetic population suggesting transmission between species [10]. Genome-wide data from a much smaller sample of worms confirms this finding [12]. We build upon those research insights using linked genetic and surveillance data from Chad to investigate the geographic connectivity of genetically defined worm populations. Spatial epidemiological models have been a key tool for inferring the connectivity of different populations and providing insights into the propagation of infectious diseases. Spatial models, such as the gravity [18] and Leǘy flight [19], have been utilized to describe the movement of humans and animals as a key component to the spread of infectious diseases [20–26]. These models and sophisticated inference algorithms have helped characterize the movement of pathogens and provide insights for programmatic

decision-making [27, 28]. Genetic data has the potential to bypass the need for proxy human movement data by *indirectly* using pathogen movement [29] even in a partially sampled transmission network [16, 30, 31]. Using genetic and epidemiological data to inform infectious disease models is a growing field, which encompasses phylodynamics [32–35]. Similar to phylogenetic methodologies, phylodynamic analyses and models are most mature for virus and bacteria pathogens [16, 27, 30, 31, 36]. However, there have been recent innovations of using the biological characteristics of parasites clonally propagating in order to build phylodynamic models of malaria parasite movement within neighborhoods of Thiés, Senegal [37]. We demonstrate that probabilistic spatial models informed by Guinea worm genetic data can reveal new insights into the geographic connectivity of worm populations in Chad.

In this article, we investigate the programmatic potential of leveraging genetic data for enhanced surveillance efforts by performing a retrospective analysis and modeling of the epidemiological and genomic data collected in Chad from 2012-2018, excluding 2014. We leverage both previously reported [10] and new genetic sequences linked with surveillance data to build spatial models that reveal a consistent geographic connectedness of worm populations in Chad. We also confirm the value of expanding the number of loci sequenced for Guinea worm in order to provide higher resolution in linkages between samples. These results are followed by a discussion on the implications of increasing the scope of genetic sequencing for programmatic surveillance and decision-making.

Materials and methods

0.1 Ethics statement

The World Health Organization and the country ministries of health have sanctioned the extraction of emergent adult worms to contain and treat Guinea worm infections. Trained program and ministry staff extract female worms from active cases and are stored in ethanol as described in Eberhard et al. [53]. Samples from human cases are anonymized prior to study inclusion.

0.2 DNA extraction and sequencing

Extraction was attempted on 712 worms collected in Chad from 2012-2018, excluding samples from 2014 due to availability. Whole genomic DNA was extracted from 5-15mm

sections of adult female worm tissue with one of two methods: a modified Puregene DNA extraction protocol as detailed in Thiele et al. 2018 [10], or with the DNeasy Blood and Tissue Kit (Qiagen, Germantown, MD, #69582) according to the manufacturer's protocol for tissue extraction.

Five hundred and ninety-five samples had successful amplification and sequencing for at least one of the three targeted mitochondrial loci (CO3, cytB, and ND3-5). Sanger sequencing and base calls from chromatograms were performed as detailed in Thiele et al. 2018 [10]. Four hundred and sixty-one samples had successful sequencing at all three targeted loci. Thirty three of these samples were reported in previous literature on the genomic characterization in Chadian Guinea worms [10]. The remaining 428 samples have not been published previously.

0.3 Alignment and variant identification

0.3.1 Targeted CO3, cytB, ND3-5 loci

Sequences for CO3, cytB, and ND3-5 for 461 worms were aligned to the *Dracunculus medinensis* mitochondria genome version JN555591.1 from the European Nucleotide Archive with the BWA v0.7.17 software package [38] to confirm amplification of the correct loci. Amplification fragments spanned the genomic coordinates of $3,690$ to $4,308$ for CO3, $2,628$ to $3,234$ for cytB, and $12,562$ to $14,523$ for ND3-5. Bases (A, C, G, T, or other for ambiguous or missing) were counted for each position in the alignment ranges for the population. Missing bases were concentrated at the beginning and/or ends of the gene ranges due to amplification variation. Positions in the alignment ranges were excluded from variant discovery if more than two samples with successful amplification for that gene had the designation of missing or ambiguous.

Positions with at least two different bases (excluding other) were variant candidates and checked for irregularities. Two worms samples exhibited highly irregular singleton characteristics, contributing more than 50% of the singleton variants in a single gene. These two worm samples were excluded from variant discovery and analyses. From the remaining worms, we identified 86 variant positions concatenated to create a molecular barcode. Forty-one unique barcodes were identified across 459 worms. Six variants in the barcode were singletons in the population. Three barcodes contained an ambiguous base 'N', assigned to four samples in the population. Barcodes were assigned an identifying number based on the number of samples belonging to each barcode. For methods describing the accumulation of barcode diversity in the population at different

loci, refer to S1 Appendix.

0.3.2 Complete mitochondria genome

Next-generation shotgun sequencing from nineteen publicly available *Dracunculus medinensis* mitochondrial genomes were downloaded from the European Nucleotide Archive (PRJEB1236) [12]. Low quality bases with a minimum mapping quality of 20 were trimmed from the ends of reads. Trimmed reads were aligned to the *D. medinensis* mitochondria genome with the BWA v0.7.17 software package [38]. Variants were called on de-duplicated, uniquely aligned reads following best practices outlined by GATK v4.1.4. Known variants are typically recommended to correct sequencing errors that lead to spurious variant calls. A set of known variants is not available for the *D. medinensis* mitochondria genome. Instead, bootstrap base recalibration was done using higher confidence calls (QD >2.0, FS >60.0, MQ <40.0, MQRankSum <-12.5, ReadPosRankSum <-8.0) until the final calls converged (2-3 steps) [39]. Final variants were filtered using the following input parameters for GATK: FS $<=$ 13.0 or missing, SOR <3.0 or missing, and $-3.1 <=$ ReadRankPos, BaseQRankSum, MQRankSum, ClippingRankSum $<=$ 3.1 [12]. Given the low sample size for recalibration, orthogonal variant calling was performed using the bcftools program [40]. Genotype maximum likelihoods were determined using the mpileup command with parameters specifying a minimum mapping quality of 20 and ploidy of 1. For both variant call sets, variants found within positions 5950-6670 were removed due to high heterozygosity and $2\times$ mapping coverage. For all other variants, a minimum depth of 10 reads was required across all samples to be included in downstream analyses.

0.4 Clustering of molecular barcodes

Discriminant analysis of principal components (DAPC) was used to infer population structure based on the molecular barcodes using the R package adegenet v2.1.3 [41–43]. Populations were defined by host identity. Barcodes found in samples from humans only were grouped with barcodes found in samples from humans and dogs to create more even groups (humans only = 5 barcodes, both = 12, dogs only = 24). To investigate whether the barcodes differentiated by host species, we specified one discriminant axis for the two classifications. We tested the inclusion of 2, 5, 10 principal components on discrimination results. The results were comparable across the tested principal component range, with overlapping distributions in the first discriminant axis.

0.5 Linking genomic samples to epidemological data

We linked the sequenced samples with corresponding surveillance data using national case reports and standardized surveys, described in detail in [44]. Case data was collected from an active and passive village-based surveillance system by the Chad Guinea Worm Eradication Program (CGWEP) and the Ministry of Public Health (MOHP) [45]. Summary case reports at the village level were compiled across surveillance sites by the CGWEP program. Information on infected dogs was collected through a standardized survey with the self-identified owner. Where available, global position system (GPS) coordinates from the standardized surveys were validated with village coordinates from the national case reports. The GPS coordinates were consistent across national case reports and standardized surveys for 207 samples. In instances where coordinates differed between the standardized surveys and the national case report, the national case report coordinates were assigned (181 samples). For cases reported with a village name in the standardized survey but without GPS coordinates, the GPS coordinates associated with the village name in the national case report was assigned to that sample (2 samples). For cases reported with a village name and GPS coordinates, but the village name was not in the national case report, standardized survey case coordinates was assigned to that sample (37 samples). Four hundred and twenty-six worms were linked across 245 hosts.

0.6 Probabilistic spatial models of Guinea worm connectivity

0.6.1 Spatial models

Spatial movement models have demonstrated that distance is an important factor influencing the spread of pathogens. [21–23, 30]. Here, due to the yearly prepatent cycle of the Guinea worm parasite [1], we specified a spatial model similar to a probabilistic diffusion model for a discrete spatial network representing the villages affected by Guinea worm in Chad. The model describes the probability of a worm being transmitted from village i to village j based on the geographic distance d between a pair of worms such that $p_{ij} = C_i \mathrm{f}(d_{ij})$. C_i is a scaling factor and f is informed by the pairwise geographic distance between pairs of worms with identical or non-identical barcode identity. Similar to other parametric formulations such as the diffusion, gravity [22], or Lévy flight models [30], our non-parametric formulation could be used to predict where a future linked Guinea worm case might appear.

0.6.2 Genetic identity and geographic distance between samples

To infer f in the model above, we used the genetic identity between samples and the associated GPS coordinates for villages. We estimated the number of variant differences across the mitochondrial genome that may connect a direct lineage (i.e. a maternal-offspring pair of worms) from a spontaneous mutation rate. Without a known organismal mutation rate, we assumed a similar mutation rate as the *Caenorhabditis elegans* spontaneous mitochondrial mutation rate estimated at $1.05e-7$ site/generation [46]. From this rate, we expected 0.0015 mutations for the $14,628$ base pair *Dracunculus medinensis* mitochondrial genome between generations. Given this extrapolated generational mutation rate less than one, worms were grouped by exact barcode identity. We did not make any assumptions of pairwise genetic distance from models of DNA evolution for analyses comparing genetic similarity between pairs of worms. We calculated the pairwise genetic similarity as 1-the Hamming distance from the molecular barcodes to obtain the number of shared variants [47]. The haversine distance was used to compute the geographic distance between each pair of worms d using the R package geosphere v1.5 [48].

Grouping barcodes by exact identity may exclude pairs of samples that share a recent lineage in the event of an underestimated mutation rate or an unlikely, but possible, sequencing error. We tested the impact of a single base pair difference in barcodes between a pair of worms on geographic clustering (see section 0.6.4). Relaxing thresholds on a pairwise basis avoided issues clustering barcodes with more than a single base pair difference. For example, barcodes A and B had two base pair differences, but barcode A and C and barcode B and C each have a single base pair difference.

0.6.3 Spatial models by molecular barcode sets

To characterize f, we partition the samples according to their genetic identity. Samples with identical barcodes are grouped; for each barcode k, all of the pairwise geographic distances within the set are used to compute a barcode functional form f_k. The set of functions f_k are then averaged to produce a single f. We also investigated the impact of partitioning samples with a less restrictive criterion based on the assumed mutation rate as described above. Similar to research in the comparison of power-law distributions [49], we utilized the empirical cumulative densities f using stat_ecdf and compare distributions using the Kolmogorov-Smirnov test with base R v3.6.3 [50]. We plotted the empirical distributions as smoothed kernel density distributions using the function stat_density contained in the R package ggplot2 v3.3.0 [51].

0.6.4 Sensitivity analyses

We investigated the results by performing a series of sensitivity analyses including statistical bootstrapping. Distance permutations between pairs of worms included 100 iterations of sampling without replacement for the all-by-all pairwise geographic distances. Multi-infected host bootstrap subsampling included 100 iterations of choosing a single case worm at random for each host. In instances where only one case is observed per host, that case was consistently the representative. Each iteration was considered an independent distribution and analyzed as described above. In addition, we also tested the impact of relaxed similarity thresholds for defining matched identity of the molecular barcodes between pairs on the geographic clustering.

0.6.5 Analyzing the diversity of genetic samples by geography

A cumulative barcode diversity score was calculated for each worm with GPS coordinate data (426 worms). The number of unique barcodes was counted for an expanding radius around each worm until every other worm in the study was included in the radius. We also investigated the effect of opportunistic sampling on barcode geographic clustering by comparing the cumulative distance from each case to every other worm in the population. From each worm, we expected the number of samples to increase at variable rates depending on sample clusters. Deviations from an increase (i.e. a sustained flat line) between samples would indicate geographic breaks between sample clusters.

0.7 Analyzing sensitivity of additional loci outside of CO3, cytB, and ND3-5

Variants in the complete mitochondrial genome were grouped by whether the variant was in the CO3, cytB, and ND3-5 loci ranges ('within loci') or in other regions of the genome ('outside of loci', including non-coding regions). Loci bounds were determined by the start and end positions of genes in the *Dracunculus medinensis* mitochondria genome version JN555591.1 instead of the amplicon alignment ranges due to the differences in sample processing and sequencing technology. The genomic ranges for the loci are $3,778$-$4,543$ for CO3, $2,619$-$3,720$ for cytB, and $12,550$-$14,467$ for ND3-5. Pairwise genetic similarity was calculated from barcodes created with all variants, variants within loci, or variants outside of loci. For bootstrap subsampling tests, variants outside of loci ranges were randomly selected to contain the same number of variants found within loci to create a new barcode for genetic similarity comparisons.

Pairwise genetic similarity comparisons for the different loci groups were robustly tested across two variant calling pipelines (GATK and bcftools mpileup, see 0.3.2) with all variants and singleton variants excluded. Smooth kernel density distributions were generated with ggplot2 v3.3.0 option "stat_density". A two sided Kolmogorov-Smirnov test was used to compare genetic similarity distributions of different loci classes with base R v3.6.3 [50].

Results

0.8 Genetic and epidemiological characteristics of Guinea worm cases.

Four hundred and fifty-nine worms (30 worms from humans hosts, 429 worms from dog hosts) collected from 2012-2018 were successfully sequenced (Fig 1A). Four hundred and twenty-six successfully sequenced worms were matched to GPS coordinates for each reported case. Worm cases mainly clustered along the Chari River (Fig 1A). Identical mitochondrial sequences were observed for cases across years (Fig 1B). Eighty six variants were identified in the CO3, cytB, and ND3-5 loci of 618, 606, and $1,961$ base pairs, respectively. The concatenation of the 86 variants resulted in 41 unique molecular barcodes (S1 Table).

Barcode accumulation curves suggest the 41 barcodes in this study did not saturate the potential diversity of the unobserved population. We estimated the number of barcodes that may be uncovered with more sequencing. A standard negative binomial and an empirical Bayes approach [52] estimated we may observe 25-40 additional barcodes for a sampled population size of 5000. Despite the differences between methods, both indicated that modestly increasing the number of samples sequenced is not likely to capture the complete underlying genetic diversity. However, the extrapolation was hindered by the available data; it is unclear whether the abundance of low-frequency barcodes in the currently sampled population are representative of the population. See S1 Appendix for further methodological details and numerical investigations.

0.9 Molecular barcode similarity and persistence between host species.

Twelve of the 41 identified barcodes were shared between human and dog hosts. There were more unique dog barcodes driven by the high number of reported cases in dogs (Fig 1A). From the years included in this study it does not appear worms were shifting from one host species to the other (S1 FigA). Barcodes were likely to appear in dogs and humans in the same year (barcodes 2, 6, 8, 10, 36), dogs a year prior to humans (barcodes 5, 7, 13, 21), or in humans a year prior to dogs (barcodes 3, 4, 14). In addition, DAPC of barcodes highlighted a challenge to discriminate between lineages of human and dog hosts suggesting they share a genetically similar Guinea worm parasite population (S1 FigB).

0.10 Genetically identical barcodes are geographically clustered.

0.10.1 Population level comparisons

Genetically identical barcodes were spatially clustered in Chad. Identical barcodes were within a median 18.6 kilometer range (standard deviation = 82.5 kilometers), and often within an approximately 50 kilometer radius (Fig 2A). Non-identical barcodes were more evenly distributed within a median 222.2 kilometers (standard deviation = 157.5 kilometers), with slight inclination to be clustered within 100 kilometers or 400 kilometers (Fig 2A). The spatial distributions between identical and non-identical barcode distributions were statistically different (Kolmogorov–Smirnov test, $p < 0.001$). Reclassifying pairs of worms with a single variant difference to account for potential underestimated mutation rates or a sequencing error as identical robustly replicated geographical clustering within a 50 kilometer radius (S2 Fig). Permuting the distance between pairs of worms removed spatial clustering for identical worms (S3 Fig).

Geographic clustering of barcodes was not due to the similarity of parasites within hosts that carry multiple infections. Sixty-five of the 245 hosts had multiple worm infections, with a median of 4 worms (range = 2-24) per multi-infected host (S4 Fig). Worms captured from the same host were not always genetically identical. The distribution of genetic similarity for worms from the same host did not differ from worms with the same GPS coordinates from different hosts (S4 Fig). Bootstrap subsampling of a single worm from each host maintained spatial clustering with low variability (S5 Fig).

0.10.2 Individual barcode comparisons

Individual barcodes showed variation in their geographical range (Fig 2B). For example in Fig 1C, cases with Barcode 2 were found in the southeast mouth of the Chari River, while cases with Barcode 3 and 4 were spread along the entire river. The geographic range for samples in each common barcode can be seen in S6 Fig. We replicated the smooth kernel density estimates for 10 of the 38 barcodes; note that three of the barcodes do not contain associated GPS locations for any sample. The geographic variability was not correlated to the total number of worms assigned to a barcode (S1 Table). Two of the barcodes seen in Fig 2B represented with pink and mint were more similar to the null distribution and found in a broader geographic range in Chad.

The variation in geographical ranges for barcode groups was not driven by a geographical isolation. For most worms, we observed a steep increase in unique barcodes up to 200 kilometers (Fig 3). Worms with a slow increase in the unique barcodes after 200 kilometers corresponded to the human cases observed furthest east in Fig 1A. These worms followed a similar steep increase once the radius includes samples along the Chari River. The plateau of diversity observed in most worms around 18-20 unique barcodes is an artifact of opportunistic sampling proximity (S7 Fig).

0.11 Expanding genetic markers can improve sensitivity for comparing worm populations.

The extraction protocol of Guinea worm from an infected host results in degraded parasite DNA and a microbial mixture that is problematic for standard extraction and untargeted sequencing protocols. This limitation has dampened large scale genetic studies of Guinea worm using additional markers on the mitochondrial and nuclear genome. It was unclear whether variants in other regions of the mitochondrial genome would significantly alter population worm relatedness to consider protocol development for other markers. To determine the value of including variants outside of the loci targeted in this study, we compared variants in other regions of the mitochondrial genome from an independent set of 19 worm samples with successful DNA extraction and shotgun sequencing [12].

Including mitochondrial variants outside of CO3, cytB, and ND3-5 loci changed the distribution of genetic similarity between pairs of worms (Fig 4). Forty-seven variants were found within loci sampled in this study, and 129 variants outside of loci. Variants within loci differentiated two peaks in the density estimates compared to all variants

and variants outside of loci that differentiated three peaks in the density estimates. The distribution of genetic similarity between worms using the 47 loci variants were statistically different than the distribution of genetic similarity using the 129 non-loci variants (Kolmogorov–Smirnov test, $p < 0.001$). The broader pairwise similarity distribution of outside loci was robust to the number of variants in the pairwise calculation (Fig 4). Distributions of randomly subsampled 47 outside loci variants compared to the distribution of 47 loci variants showed that in some instances the population was less genetically identical with major peaks shifted to the left, and maintaining a higher density in the 0.75-0.85 pairwise genetic similarity range. However, because of the small sample size without accompanying GPS coordinates of this independent population, we could not extrapolate the effect of barcode sets on the spatial links between identical and non-identical pairs.

Despite requiring a minimum of 10 reads for each variant at a position to ensure true variant signals, we additionally confirmed the change in pairwise genetic similarity excluding variants observed in one sample in the population. Excluding variants found in only one worm, we dropped from 176 variants to 135 variants for comparison across the mitochondrial genome. The shape of the pairwise distribution including variants identified outside of the CO3, cytB, and ND3-5 loci had three mass peaks compared to two mass peaks when only considering variants within the CO3, cytB, and ND3-5 loci (S8 Fig, Kolmogorov–Smirnov test $p = 0.001$). This robust trend also observed when including singleton variants showed the pairwise distance between pairs or worms was not driven by unique variants in the population. Variants were called with an orthogonal method to circumvent any issues with genotype calling on a small sample size in GATK. More variants across the mitochondrial genome were identified calling with bcftools than GATK (185 versus 175 variants). Variants calls with bcftools replicated the different distributions for variants within loci and outside loci with a new mass peak around 0.75 pairwise genetic similarity. (Kolmogorov–Smirnov test, $p = 0.001$, S9 Fig).

Discussion

To our knowledge, this is the largest retrospective study linking surveillance and genetic data to understand Guinea worm transmission in Chad to date, encompassing samples from 2012-2018, excluding 2014. Knowing the geographical range of transmission for genetically linked samples has the potential to be an important monitoring and evaluation tool for the campaign. We identified 41 unique molecular barcodes from the

459 Chadian worm samples. Among the 459 samples, 426 samples had associated GPS data. The analyses in this study provide quantitative boundaries on the geographic range of most transmission within a median 18.6 kilometer radius, and often within an approximately 50 kilometer radius. These results suggest the most effective interventions should consider case sweeps, water monitoring, and abatement approximately 20 kilometers around a reported case to reduce the spread of genetically related parasites. The population distribution falling under 50 kilometers implies that extending these efforts from 20-50 kilometers would largely dampen the dispersion of genetically related worms, with diminishing returns for efforts greater than 50 kilometers from a reported case. However, worms linked by barcode identity are not geographically isolated populations. Samples are surrounded by worms representing five to ten different barcodes within a 50 kilometer radius (Fig 3), and individual barcodes can vary in their transmission range from three to 150 kilometers. Geographically imposed interventions from these results need to consider the dispersion range variation for cases identified in areas with co-occurring lineages.

The modeling results presented in this article are consistent with previous analyses, but also expand the scope of geographic and genetic relatedness. Overlapping barcodes of worms collected from human and dog hosts supports the earlier conclusion by Thiele et al. [10] that humans and dogs share a similar Guinea worm population in their analysis of 75 samples (S1 FigA). Barcodes did not consistently shift from one host species over time, suggesting a fluid transmission of *D. medinensis* between humans and dogs. Previous genetic analyses using spatial principal components analysis had identified a geographic trend of genetic relatedness down the Chari River in Chad [10, 44, 53]. The research in this article broadens that analysis by revealing that genetically identical and near-identical samples cluster geographically for multiple areas across Chad. (Fig 1C).

The findings of this work also align with the known epidemiology of the disease and biology of the parasite. Recent surveillance efforts involving the collaring and GPS tracking of dogs in Chad showed dogs visit water sources within a 10 kilometer range with variation across study sites [54]. The substantial geographic distance distribution for genetically identical pairs of worms under 50 kilometers are broadly in agreement with the dog roaming range and variation by geography (Fig 2). Both of these research efforts support the current epidemiological intuition and hypotheses about geographic connectivity of infections and the role of dogs as a reservoir [10, 45, 54].

The numerous sets of identical or nearly identical molecular barcodes among the 459

samples was expected. We anticipated some barcode sets would be maintained throughout the few years represented in this study due to the parasite biology of a year-long prepatent period and the suspected low spontaneous mutation rate (see section 0.8). Even though the probability of having observed a direct transmission event in the available genetic samples is quite low (given the total number of reported cases), the molecular barcodes demonstrate distinct value even though the transmission network is only partially observed. The direct maternal inheritance of mitochondrial DNA and the persistence of barcodes across available years suggests a sustained population of worms are related through transmission. Taken together, these findings strongly suggest an epidemiological connection identified using the genetic data and can help inform the local epidemiology of Guinea worm in Chad.

There are several limitations to the analysis and modeling in this article. The samples that were sequenced and included in this analysis were retrospectively selected in order to span the geography of Chad and not through a systematic sampling frame. As mentioned, this led to an absence of 2014 samples in this study. We evaluated the robustness of each conclusion with sensitivity analyses to address the challenges posed by data constraints and methodologies (Fig 1 and 2) We demonstrated the geographic clustering results were not sensitive to dogs with multiple worm infections which could have artificially inflated the geographic proximity between pairs of worms (S5 Fig).

In addition, the characteristics of the *Dracunculus medinensis* genome is not as well-understood as viruses, bacteria, or other parasites. Without knowledge of baseline genetic diversity or a mutation rate for Guinea worm, we could not more accurately assess the genetic similarity threshold to group lineages. Despite these constraints, we showed that allowing for a single base pair difference in barcodes between a pair of worms maintained spatial clustering relative to two or more base pair differences in barcodes between a pair of worms (S2 Fig). Lineage representation is likely limited by the use of three mitochondrial loci coupled with the small sample size relative to the number of observed and unobserved total cases from 2015-2017. In an independent population of samples with complete coverage of the mitochondrial genome, we confirmed variants in other regions of the mitochondrial genome changed the distribution of population pairwise genetic relatedness (see section 0.11). This trend was replicated with a combination of bootstrapping and variant callers. Due to small sample sizes of available whole mitochondrial DNA, we were unable to conclude whether the extra variants refine geographic clustering. The substantial number of additional variants in the mitochondrial genome strongly suggests that access to more of the

genome will better resolve identical lineages for Guinea worm.

Despite these limitations, the modeling and analyses in this article have important implications for policy makers and elimination programs. From 2010-2019, there was a concurrent increase in both reported cases and surveillance efforts. A complete characterization of the genetic diversity could help distinguish whether Guinea worm prevalence was increasing or a consequence of improved surveillance. The continued appearance of genetically identical worms across years suggests genomic data is informative for understanding transmission, surveillance, and even interventions. Monitoring the genetic landscape could provide programmatic evidence for the effectiveness of geographically localized interventions by observing the potential elimination of barcode lineages. Sustained barcodes are particularly useful in instances where case reports may be disrupted due to insecurity or inaccessibility.

A comprehensive bank of all genomic samples paired with geographic data would allow a broader set of analyses to help the elimination program; for example, outbreak analysis, importation versus local circulation characterization, and potentially reveal unknown animal reservoirs. We cannot provide programmatic guidance on a specific number of samples that should be collected and sequenced to capture the genetic diversity in Chad due to the high diversity of the *currently* sampled population (S1 Appendix). If a subset of historical samples share barcodes with the currently sampled population we would likely be powered to define a sample size for genetic surveillance. Conversely, more unique barcodes in historical samples would support the need to sequence all available worm samples due to the high diversity of the population. Current surveillance protocols already collect emerging worms in addition to a standardized survey for all reported cases. Given the reported case counts and access to high-throughout sequencing technologies, it is tractable to sequence a large subset or all of the available retrospective and prospective Guinea worm samples.

The confirmed change in population pairwise genetic similarity across the whole mitochondrial genome suggests expanding the marker set is an important future research direction. An expanded marker set would complement innovations constructing phylogenies from whole genome sequencing of the Guinea worm larvae [55] and microsatellites of the worm nuclear genome [10]. A holistic program of sequencing strategies and analytic methodologies will help translate research insights into programmatic input for the elimination of Guinea worm in Chad. Furthermore, additional analyses of the full range of epidemiological data collected by the CGWEP alongside linked genomic data are warranted, and may further elucidate transmission

dynamics in Chad. The parasite genome has the potential to be an integral tool for the end-game strategy in Chad and beyond.

Acknowledgments

We would like to thank Dr. Fernando Torres for his helpful discussions regarding Guinea worm eradication efforts and Dr. Albert Lee for modeling discussions. This work would not be possible without the field and surveillance teams of the national Guinea worm eradication program in Chad. Thank you for the efforts of all involved to collect the epidemiological data and samples.

Funding statement

This publication is based on research funded in part by the Bill & Melinda Gates Foundation, including but not limited to models and data analysis performed by the Institute for Disease Modeling at the Bill & Melinda Gates Foundation by JVR, NL, HL, GCC, JLP. JAC was supported by funding from The Carter Center and Wellcome, via their core support for the Wellcome Sanger Institute (grant WT206194). The funders had no role in study design, data collection and analysis, decision to publish, or preparation of the manuscript.

Data availability

Genomic sequences are available on Genbank as MW257241 - MW257699 for CO3, MW257700 - MW258158 for cytB, and MW258159 - MW258617 for ND3-5 loci. The epidemiological data containing the case coordinates must be requested from and approved by the Chad Ministry of Public Health (contact via Tchonfienet Moundai, tchomcalvin@yahoo.fr). Analysis code can be accessed at https://github.com/InstituteforDiseaseModeling/GWSpatialGenetics.

Disclaimer

The findings and conclusions in this report are those of the author(s) and do not necessarily represent the official position of the Centers for Disease Control and Prevention/the Agency for Toxic Substances and Disease Registry.

References

1. Biswas G, Sankara DP, Agua-Agum J, Maiga A. Dracunculiasis (guinea worm disease): eradication without a drug or a vaccine. Philosophical Transactions of the Royal Society B: Biological Sciences. 2013;368(1623):20120146.

2. Hopkins DR, Weiss AJ, Roy SL, Zingeser J, Guagliardo SAJ. Progress Toward Global Eradication of Dracunculiasis—January 2018–June 2019. Morbidity and Mortality Weekly Report. 2019;68(43):979.

3. World Health Organization: Dracunculiasis eradication;. https://www.who.int/dracunculiasis/certification/en/.

4. Sreenivasan N, Weiss A, Djiatsa JP, Toe F, Djimadoumaji N, Ayers T, et al. Recurrence of Guinea worm disease in Chad after a 10-year absence: risk factors for human cases identified in 2010–2011. The American Journal of Tropical Medicine and Hygiene. 2017;97(2):575–582.

5. Center for Disease Control and Prevention: Morbidity and Mortality Weekly Report. Progress Toward Global Eradication of Dracunculiasis- January 2018 -June 2019;. https://www.cdc.gov/mmwr/volumes/68/wr/mm6843a5.htm.

6. Callaway E. Dogs thwart effort to eradicate Guinea worm. Nature News. 2016;529(7584):10.

7. Eberhard ML, Cleveland CA, Zirimwabagabo H, Yabsley MJ, Ouakou PT, Ruiz-Tiben E. Guinea worm (Dracunculus medinensis) infection in a wild-caught frog, Chad. Emerging Infectious Diseases. 2016;22(11):1961.

8. Molyneux D, Sankara DP. Guinea worm eradication: Progress and challenges—should we beware of the dog? PLoS Neglected Tropical Diseases. 2017;11(4).

9. Cleveland CA, Eberhard ML, Thompson AT, Smith SJ, Zirimwabagabo H, Bringolf R, et al. Possible role of fish as transport hosts for Dracunculus spp. larvae. Emerging Infectious Diseases. 2017;23(9):1590.

10. Thiele EA, Eberhard ML, Cotton JA, Durrant C, Berg J, Hamm K, et al. Population genetic analysis of Chadian Guinea worms reveals that human and non-human hosts share common parasite populations. PLOS Neglected Tropical Diseases. 2018;12(10):e0006747.

11. The Carter Center and the Center for Disease Control and Prevention: Guinea worm Wrap-Up, #267—April 7 2020;. https://www.cartercenter.org/resources/pdfs/news/health_publications/guinea_worm/wrap-up/267.pdf.

12. Durrant C, Thiele EA, Holroyd N, Doyle SR, Sallé G, Tracey A, et al. Population genomic evidence that human and animal infections in Africa come from the same populations of Dracunculus medinensis. PLOS Neglected Tropical Diseases. 2020;14(11):e0008623.

13. Garrett KB, Box EK, Cleveland CA, Majewska AA, Yabsley MJ. Dogs and the classic route of Guinea Worm transmission: an evaluation of copepod ingestion. Scientific Reports. 2020;10(1):1–5.

14. Pallansch MA, Sandhu HS. The eradication of polio—progress and challenges. New England Journal of Medicine. 2006;355(24):2508–2511.

15. Cowger TL, Burns CC, Sharif S, Gary Jr HE, Iber J, Henderson E, et al. The role of supplementary environmental surveillance to complement acute flaccid paralysis surveillance for wild poliovirus in Pakistan–2011–2013. PLOS ONE. 2017;12(7).

16. Famulare M, Hu H. Extracting transmission networks from phylogeographic data for epidemic and endemic diseases: Ebola virus in Sierra Leone, 2009 H1N1 pandemic influenza and polio in Nigeria. Int Health. 2015;7(2):130–138.

17. Dalmat R, Naughton B, Kwan-Gett TS, Slyker J, Stuckey EM. Use cases for genetic epidemiology in malaria elimination. Malaria Journal. 2019;18(1):163.

18. Tinbergen J. Shaping the world economy; suggestions for an international economic policy. vol. 46. Twentieth Century Fund, New York; 1962.

19. Bayati BS. Fractional diffusion-reaction stochastic simulations. The Journal of Chemical Physics. 2013;138(10):104117.

20. Brockmann D, Hufnagel L, Geisel T. The scaling laws of human travel. Nature. 2006;439(7075):462–465.

21. Riley S. Large-Scale Spatial-Transmission Models of Infectious Disease. Science. 2007;316(5829):1298–1301. doi:10.1126/science.1134695.

22. Balcan D, Colizza V, Gonçalves B, Hu H, Ramasco JJ, Vespignani A. Multiscale mobility networks and the spatial spreading of infectious diseases. Proc Natl Acad Sci USA. 2009;106(51):21484–21489. doi:10.1073/pnas.0906910106.

23. Yingcun X, Bjornstad ON, Grenfell BT. Measles Metapopulation Dynamics: A Gravity Model for Epidemiological Coupling and Dynamics. Am Nat. 2004;164(2):267–281. doi:10.1086/422341.

24. Jandarov R, Haran M, Bjornstad O, Grenfell B. Emulating a gravity model to infer the spatiotemporal dynamics of an infectious disease. J R Stat Soc Ser C Appl Stat. 2014;63(3):423–444. doi:10.1111/rssc.12042.

25. Viswanathan GM. Ecology: Fish in Levy-flight foraging. Nature. 2010;465:1018–1019.

26. Humphries NE, Weimerskirch H, Queiroz N, Southall EJ, Sims DW. Foraging success of biological Lévy flights recorded in situ. Proc Natl Acad Sci USA. 2012;109(19):7169–7174. doi:10.1073/pnas.1121201109.

27. Lemey P, Rambaut A, Bedford T, Faria N, Bielejec F, Baele G, et al. Unifying Viral Genetics and Human Transportation Data to Predict the Global Transmission Dynamics of Human Influenza H3N2. PLOS Pathogens. 2014;10(2):1–10. doi:10.1371/journal.ppat.1003932.

28. Backer JA, Wallinga J. Spatiotemporal analysis of the 2014 Ebola epidemic in West Africa. PLOS Computational Biology. 2016;12(12):1–17.

29. Klepac P, Metcalf CJE, McLean AR, Hampson K. Towards the endgame and beyond: complexities and challenges for the elimination of infectious diseases; 2013.

30. Gustafson KB, Proctor JL. Identifying spatio-temporal dynamics of Ebola in Sierra Leone using virus genomes. Journal of the Royal Society Interface. 2017;14(136):20170583.

31. Didelot X, Fraser C, Gardy J, Colijn C. Genomic Infectious Disease Epidemiology in Partially Sampled and Ongoing Outbreaks. Mol Biol Evol. 2017;34(4):997. doi:10.1093/molbev/msw275.

32. Rasmussen DA, Ratmann O, Koelle K. Inference for Nonlinear Epidemiological Models Using Genealogies and Time Series. PLOS Computational Biology. 2011;7(8):1–11. doi:10.1371/journal.pcbi.1002136.

33. Ypma RJF, van Ballegooijen WM, Wallinga J. Relating Phylogenetic Trees to Transmission Trees of Infectious Disease Outbreaks. Genetics. 2013;doi:10.1534/genetics.113.154856.

34. Rasmussen DA, Boni MF, Koelle K. Reconciling Phylodynamics with Epidemiology: The Case of Dengue Virus in Southern Vietnam. Molecular Biology and Evolution. 2013;31(2):258.

35. Rasmussen DA, Volz EM, Koelle K. Phylodynamic Inference for Structured Epidemiological Models. PLOS Computational Biology. 2014;10(4):1–16.

36. Jombart T, Cori A, Didelot X, Cauchemez S, Fraser C, Ferguson N. Bayesian Reconstruction of Disease Outbreaks by Combining Epidemiologic and Genomic Data. PLOS Computational Biology. 2014;10(1):1–14. doi:10.1371/journal.pcbi.1003457.

37. World Helath Organizaton: Technical consultation on the role of parasite and anopheline genetics in malaria surveillance; 2019. `https://www.who.int/malaria/mpac/mpac-october2019-session7-report-consultation-on-genomics.pdf`.

38. Li H, Durbin R. Fast and accurate short read alignment with Burrows–Wheeler transform. Bioinformatics. 2009;25(14):1754–1760. doi:10.1093/bioinformatics/btp324.

39. Van der Auwera GA, Carneiro MO, Hartl C, Poplin R, del Angel G, Levy-Moonshine A, et al. From FastQ Data to High-Confidence Variant Calls: The Genome Analysis Toolkit Best Practices Pipeline. Current Protocols in Bioinformatics. 2013;43(1):11.10.1–11.10.33. doi:10.1002/0471250953.bi1110s43.

40. Li H. A statistical framework for SNP calling, mutation discovery, association mapping and population genetical parameter estimation from sequencing data. Bioinformatics. 2011;27(21):2987–2993. doi:10.1093/bioinformatics/btr509.

41. Jombart T, Devillard S, Balloux F. Discriminant analysis of principal components: a new method for the analysis of genetically structured populations. BMC Genetics. 2010;11(1):94. doi:10.1186/1471-2156-11-94.

42. Jombart T. adegenet: a R package for the multivariate analysis of genetic markers. Bioinformatics. 2008;24:1403–1405. doi:10.1093/bioinformatics/btn129.

43. Jombart T, Ahmed I. adegenet 1.3-1: new tools for the analysis of genome-wide SNP data. Bioinformatics. 2011;doi:10.1093/bioinformatics/btr521.

44. Guagliardo SAJ, Roy SL, Ruiz-Tiben E, Zirimwabagabo H, Romero M, Chop E, et al. Guinea worm in domestic dogs in Chad: A description and analysis of surveillance data. PLOS Neglected Tropical Diseases. 2020;14(5):1–17. doi:10.1371/journal.pntd.0008207.

45. Eberhard ML, Ruiz-Tiben E, Hopkins DR, Farrell C, Toe F, Weiss A, et al. The peculiar epidemiology of dracunculiasis in Chad. The American Journal of Tropical Medicine and Hygiene. 2014;90(1):61–70.

46. Denver DR, Morris K, Lynch M, Vassilieva LL, Thomas WK. High Direct Estimate of the Mutation Rate in the Mitochondrial Genome of Caenorhabditis elegans. Science. 2000;289(5488):2342–2344. doi:10.1126/science.289.5488.2342.

47. Hamming RW. Error Detecting and Error Correcting Codes. Bell System Technical Journal. 1950;29(2):147–160. doi:10.1002/j.1538-7305.1950.tb00463.x.

48. Hijmans RJ. geosphere: Spherical Trigonometry; 2019. Available from: `https://CRAN.R-project.org/package=geosphere`.

49. Clauset A, Shalizi CR, Newman MEJ. Power-Law Distributions in Empirical Data. SIAM Review. 2009;51(4):661–703.

50. Team RC. R: A Language and Environment for Statistical Computing; 2020. Available from: `https://www.R-project.org/`.

51. Wickham H. ggplot2: Elegant Graphics for Data Analysis. Springer-Verlag New York; 2016. Available from: `https://ggplot2.tidyverse.org`.

52. Efron B. Large-scale inference: empirical Bayes methods for estimation, testing, and prediction. vol. 1. Cambridge University Press; 2012.

53. Eberhard ML, Ruiz-Tiben E, Hopkins DR, Farrell C, Toe F, Weiss A, et al. The Peculiar Epidemiology of Dracunculiasis in Chad. The American Journal of Tropical Medicine and Hygiene. 2014;90(1):61–70. doi:10.4269/ajtmh.13-0554.

54. McDonald RA, Wilson-Aggarwal JK, Swan GJ, Goodwin CE, Moundai T, Sankara D, et al. Ecology of domestic dogs Canis familiaris as an emerging reservoir of Guinea worm Dracunculus medinensis infection. PLOS Neglected Tropical Diseases. 2020;14(4):e0008170.

55. Cotton JA, Berriman M, Dalén L, Barnes I. Eradication genomics—lessons for parasite control. Science. 2018;361(6398):130–131.

Fig 1. Characteristics of genetically characterized Guinea worm cases in Chad from 2012**-**2018**.** A.) Number of cases per host species collected from 2012-2018, with GPS matched sample distributions across the Chari River. B.) The number of GPS matched samples that belong to barcode sets. Note, not all barcode colors are shown in the left figure. Barcodes with less than 10 samples in the population are colored in light gray for visual clarity. C.) Samples belonging to barcode sets 2, 3 and 4 are shown in their respective ranges. Smoothed kernel densities for the three barcode sets show the mass of the distributions correlate with the spatial connectedness of samples.

Fig 2. Distance density estimates of pairwise similarity for identical and non-identical barcodes. A.) Pairwise comparisons of identical barcodes in black (n=$10,695$) to a null of non-identical barcodes in gray (n=$79,830$). This distribution at 400 kilometers is the max distance between sampled cases along the Chari River. This enrichment of pairwise sample distance around 400 kilometers should not be considered the transmission upper bound. B.) The number of comparisons for each barcode are the unique pairs of all worms sharing that barcode identifier. The null distribution in gray of non-identical barcodes is subset to only include comparisons where one of the common barcodes must be found in the pair ($n = 56,913$).

Fig 3. Opportunistic sampling does not drive apparent spatial connectedness of samples. Accumulation curves of worm cases on geographical proximity of barcode diversity shows a consistent addition of diversity in quantitatively identified transmission ranges of barcodes sets ($<$50 kilometers). The left figure shows all accumulation curves for each worm case, and the right figure is separated by the barcode set number (1-10 for common barcodes, other for barcodes found in less than 10 samples). The numbers in the lower right corner are the number of worms in each barcode set.

Fig 4. Distribution of all-by-all pairwise genetic similarity of variants within or outside of currently sampled loci. Within loci variants were determined with CO3, cytB, and ND3-5 genomic ranges specified in the mitochondrial reference genome. Smoothed density kernel distributions are overlayed on pairwise genetic similarity distribution histograms for the different genomic regions.

Supporting information

S1 Appendix. Predicting the increase in barcode variety with additional sequencing.

S1 Table. Barcode counts for sequenced samples. GPS columns indicate the number of samples for each barcode used in geospatial analyses by species. Columns starting with 'All' indicates the number of worms assigned to each barcode, including samples that were not linked to GPS data for each host species. Temporal presence/absence of barcodes can be tracked in S1 FigA per species.

S1 Fig. Molecular barcode comparison of worm barcodes identified per species.
A. The presence of different barcodes per host by year. A blue filled cell next to each barcode is indicative that barcode was represented at least once in the respective year. Refer to S1 Table for sample counts of each barcode by species. B. Discriminant analysis of principal components on the molecular barcodes by host. Barcodes found in samples from humans only were grouped with barcodes found in samples from humans and dogs to create more even groups with barcodes found only in samples from dogs (humans only = 5 barcodes, both = 12, dogs only = 24). The overlap of discriminant distributions predicted with five principal components suggests molecular barcodes do not differ by host.

S2 Fig. Spatiotemporal modeling of barcode relatedness collapsed by a single base pair difference. Number of pairwise comparisons for identical barcodes $= 13,967$, for non-identical barcodes $= 76,558$.

S3 Fig. Permutation of distance between cases on barcode relatedness densities. The x-axis is the permuted distance between pairs of worms and the y-axis represents the density for population genetic similarity scores for all worms (n=426). Each line represents worm pair distance permutation (n=100) for the population. The lines for identical and non-identical barcodes are consistent between permutations and overlap.

S4 Fig. Barcode identity within hosts and genetic similarity distribution by shared location.
A. Each multi-infected host with the number of worms pertaining to each barcode set. "Not common" refers to barcodes found in less than 10 samples in the population for visual clarity.
B. Distributions of genetic similarity between worms with the same reported GPS coordinates, colored by whether the pair is obtained from the same or different hosts.

S5 Fig. Host subsampling on barcode relatedness densities. The x-axis is the distance between pairs of worms and the y-axis represents the density for population genetic similarity scores with one worm per host (n = 282). Each line represents a bootstrap (n=100) of a single worm per host.

S6 Fig. Location of common and not common barcodes in Southern Chad. Samples are colored by their barcode identifier, and shapes represent the host species. Barcodes 3, 4, and 10 appear have similar spatial distributions. Barcodes 2 and 5 have similar spatial distributions and are highly focal. Some barcodes span a very large geographic area, which could suggest they are ancestral sequences that have diffused over time or are transmitting due to human behaviors.

S7 Fig. Geographic distance between cases for each worm. The geographic distance between worms relative to a single worm were organized in ascending order. The x axis is the cumulative worm count by distance and the y axis is the cumulative distance from each index worm. Spans of a flattened curve are indicative of geographic stretches that do not contain any samples and support the lack of barcode diversity observed in certain geographic distances in Fig 3.

S8 Fig. Distribution of pairwise similarity using GATK variants found in greater than one worm. The x-axis is the measured genetic similarity for variants within current loci (n=35) and extending to the rest of the mitochondrial genome (n=100), and y-axis is the number of pairwise comparisons (19*19 = 361). Filled regions show the smoothed density estimates for histograms.

S9 Fig. Orthogonal validation of population pairwise similarity using variants identified by bcftools mpileup. The x-axis is the measured genetic similarity for variants within current loci (n=47) and extending to the rest of the mitochondrial genome (n=138), and y-axis is the number of pairwise comparisons (19*19 = 361). Filled regions show the smoothed density estimates for histograms.

Figure 1

Click here to access/download;Figure;Figure1.tif

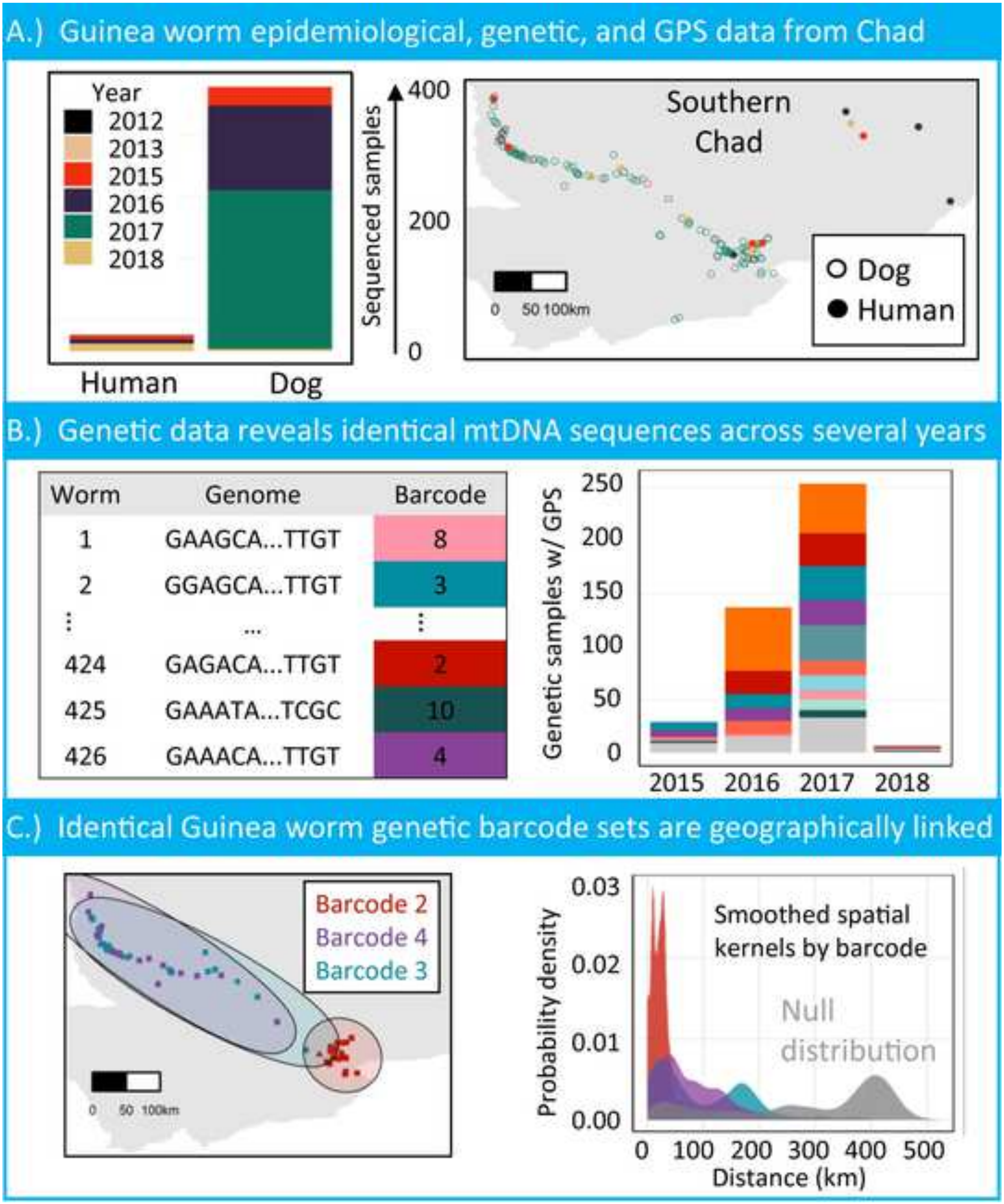

Figure 2

Click here to access/download;Figure;Figure2.tif

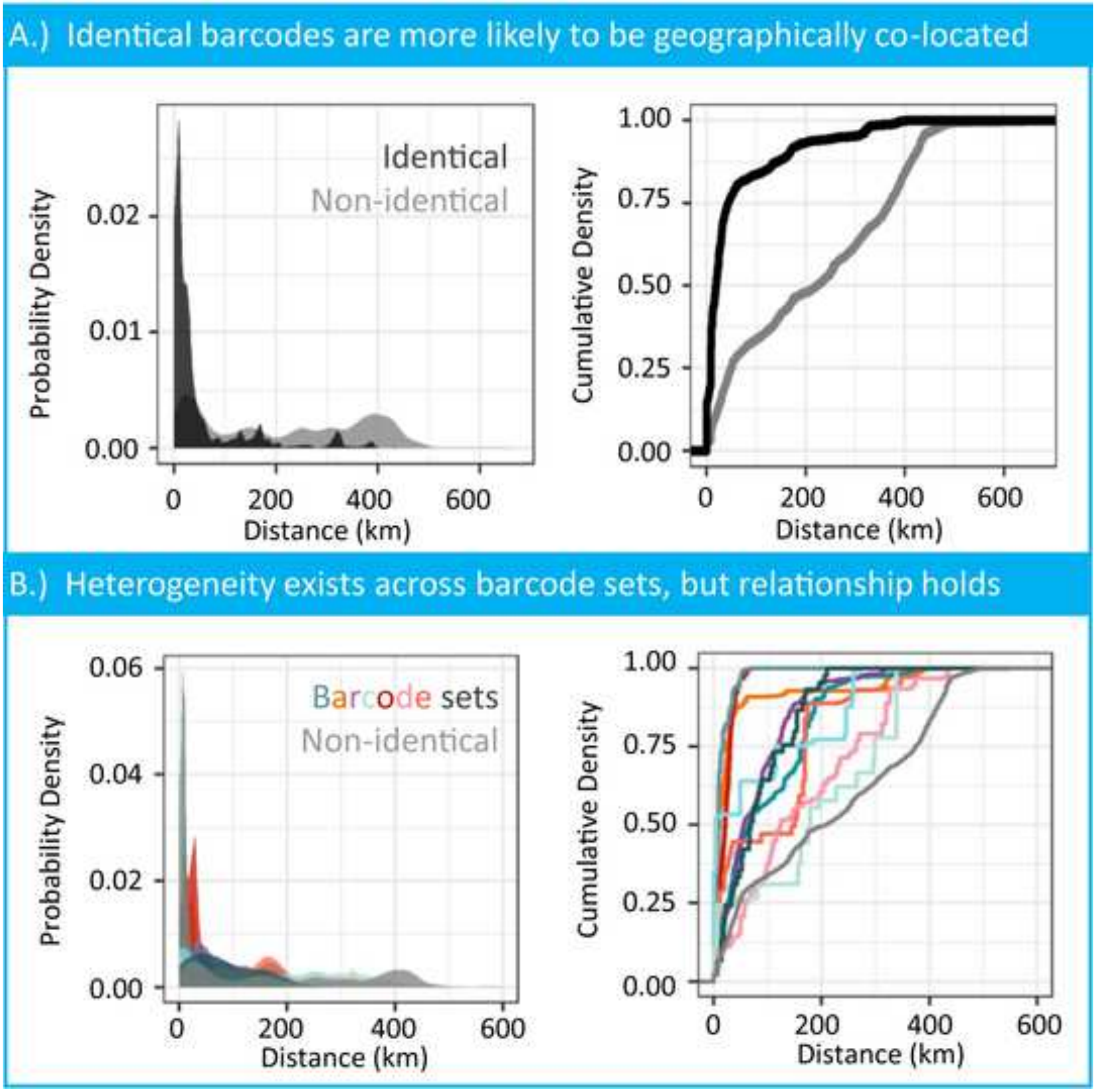

Click here to access/download;Figure;Figure3_updatedTitle.tif

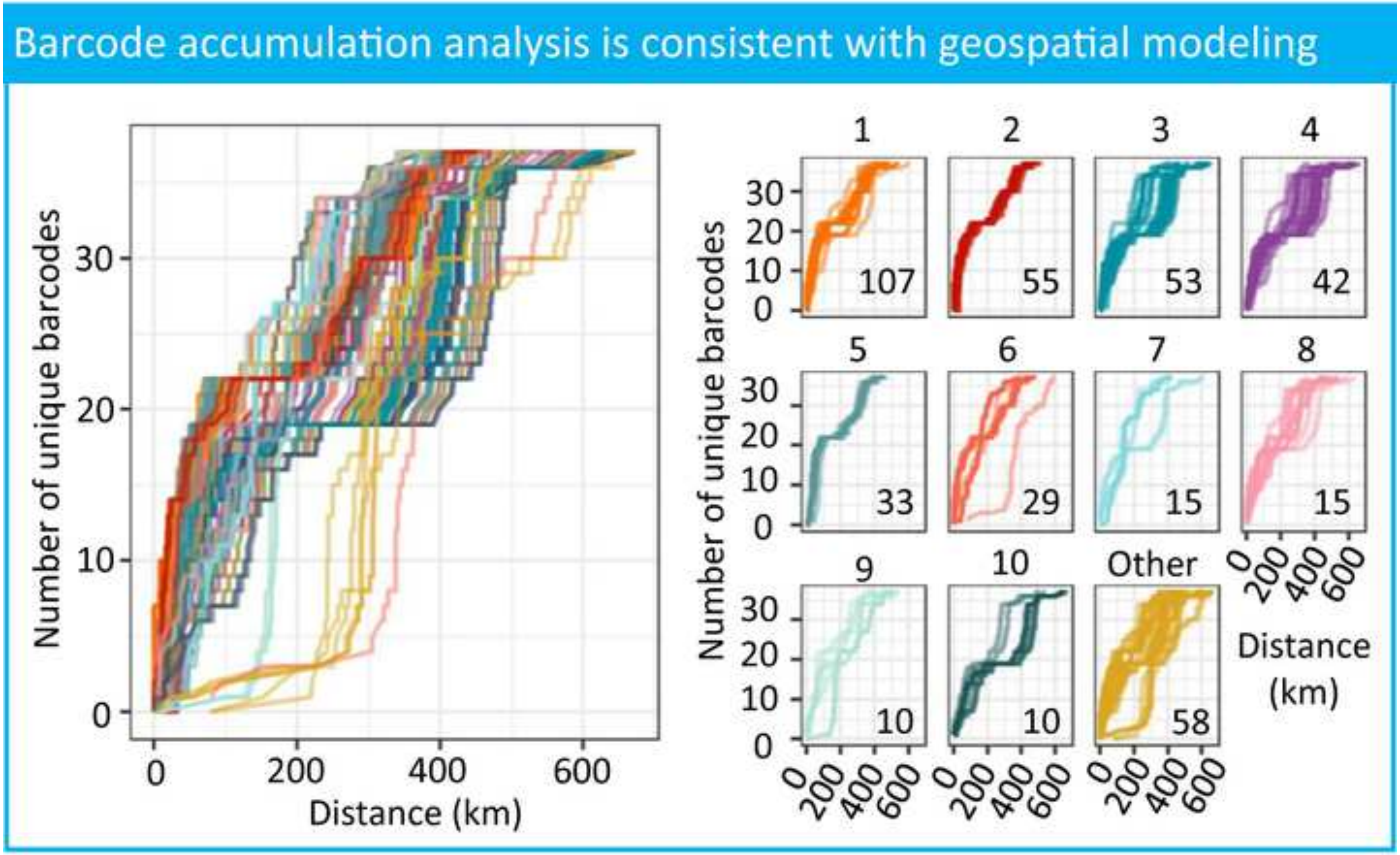

Figure 4

Click here to access/download;Figure;Figure4.tif

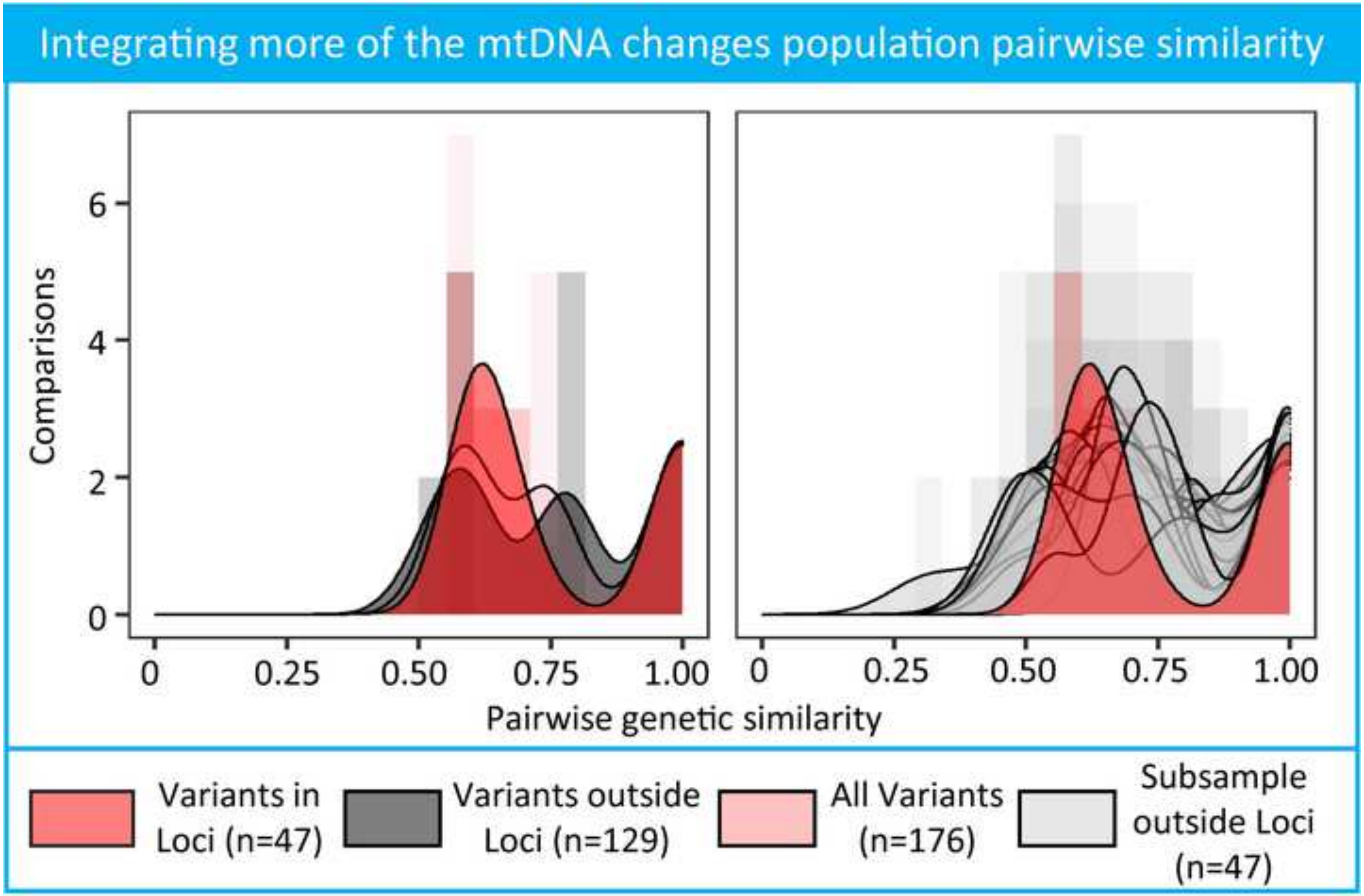

Click here to access/download
Supporting Information
SupplementAppendix_202103.pdf

S1 Table

Click here to access/download

Supporting Information

S1Table.pdf

S1 Fig

Click here to access/download
Supporting Information
S1Fig_dapc.tif

S2 Fig

Click here to access/download
Supporting Information
S2Fig_BC1Off.tif

S3 Fig

Click here to access/download
Supporting Information
S3Fig_densityDistPerm.tif

S4 Fig

Click here to access/download

Supporting Information

S4Fig_hostCounts.tif

S5 Fig

Click here to access/download
Supporting Information
S5Fig_densitySingHost.tif

S6 Fig

Click here to access/download
Supporting Information
S6Fig_mapBarcodeColors.tif

S7 Fig

Click here to access/download
Supporting Information
S7Fig_wormRarefactionDistances.tif

S8 Fig

Click here to access/download

Supporting Information

S8Fig_gatkParams_rm5950-6670_singEx.tif

S9 Fig

Click here to access/download
Supporting Information
S9Fig_bcftoolsVariantCheck.tif

We are grateful to the editors and reviewers for their thoughtful comments that have significantly improved the manuscript.

We have provided a point-by-point response to the comments below. Changes in the revised manuscript are highlighted in blue. To briefly summarize how we addressed recurring comments from reviewers:

1) We added details describing how true variant singletons were distinguished from noise. True singleton variants were retained in the molecular barcode generated from all variants identified in the sampled population.
2) We removed the supplemental phylogenetic analysis from our study following a recommendation to use a better population analysis method for our goal to distinguish barcodes by host species.
3) For the complete mitochondrial genome analyses in an independent Guinea worm population, we added the motivation for exploring more variants across the mitochondrial genome. Additionally, we added supporting details that "within loci" covers any variants that fall within the genomic ranges of the three CO3, cytB, and ND3-5 loci targeted in the spatial analyses. "Outside loci" covers any variants found in coding and non-coding regions of the genome outside of the ranges for the three loci.
4) A "Data Availability" section with the accession numbers for sequencing data has been provided. In-country partners are the owners of the epidemiological data and cannot be shared without their approval. We have added this statement along with the contact information for other researchers interested in getting data access to this section.

From the editor:
The reviewers emphasized a few points that I also noticed and that need to be addressed. First among these is the point about “singletons” and how they were treated. I found this very hard to follow but understanding what you mean is important for comprehension of later analyses. A simple, brief, explanation is likely possible and I hope to see that in a revised version.

Thank you for this important comment. We have made changes to the manuscript to improve the clarity of the description of singletons. Specifically, singleton variants identified at positions were checked for randomness across samples. Two samples that had more than 50% of the singleton variants in two different genes were considered sequencing and alignment artifacts and were removed from the study. Six singletons that were found in different samples across the three mitochondrial loci amplified in the study were retained as true variants.

For the complete mitochondrial genome analyses, we perform the same analyses with singleton variants included and singleton variants excluded due to the low number of samples (19). We checked the genetic pairwise differences between worms was not an artifact of single variants due to the large number of variants (>100) relative to the population size. The robust changes in the shape of the pairwise genetic similarity distribution with the inclusion or exclusion of singleton variants suggests that the distribution of relatedness is not an artifact of including more, lower frequency variants.

The mitochondrial genome in animals is not subject to recombination: it is effectively a single locus. Different parts of the mt genome may accumulate mutations at different rates, but all should tell the same story, subject to the range of error. So, I was confused at first about use of the word “locus” and the idea that some variants were apparently outside loci and others inside. It became clear that you were referring to a protein-coding gene as a “locus” and therefore regarded variants in non-coding regions as “outside” the locus. I found this usage confusing. It would be better to replace “locus” with “protein-coding gene” or similar.

Thank you for this observation. Locus is used to describe the genomic ranges of the four genes, CO3, cytB, and ND3-5 that were targeted for amplification. Variants identified ‘outside the loci’ are any variants that fall in any portion of the genome that does not overlap the genomic ranges of the amplified genes. We have updated the manuscript to provide this definition for within and outside the loci.

Other small points.
Line 7. I don’t think that “dracunculiasis” should start with an upper-case letter.
Correct capitalization has been updated in the manuscript.

Line 10. Hyphenate “decade-long”.
The manuscript has been updated with this edit.

Line 79. “Alleles”? I would have called them “variants”, as elsewhere.

We have updated the manuscript to be consistent with variant terminology.

Line 232.”…30 human hosts…”
The manuscript has been updated to include “worms from [species] host”

Line 233. “Of” at the end of the line seems unnecessary.
Additional word removed from the manuscript.

Line 266. What is meant by “Only four barcodes from this set (7, 21, 40 and 41) were observed as a singular event.” Maybe “only four barcodes were each found in a single worm”? In places, the language is over-complicated: this is one example.
This section of the manuscript was rewritten and condensed for clarity. The counts of individual barcodes and their respective host(s) are referenced in S1 Table. We have also attempted to address other instances of over-complicated language throughout the manuscript.

Line 423. Something is wrong here. The word “between” should maybe be deleted.
This sentence has been rewritten.

Caption to Fig. 2 has a couple of typos.
Typos have been corrected in all manuscript figures.

Reviewer's Responses to Questions
Key Review Criteria Required for Acceptance?
As you describe the new analyses required for acceptance, please consider the following:
Methods
-Are the objectives of the study clearly articulated with a clear testable hypothesis stated?
-Is the study design appropriate to address the stated objectives?
-Is the population clearly described and appropriate for the hypothesis being tested?
-Is the sample size sufficient to ensure adequate power to address the hypothesis being tested?
-Were correct statistical analysis used to support conclusions?
-Are there concerns about ethical or regulatory requirements being met?

Reviewer #1: The objectives of the study are articulated; the study design is appropriate; the population is clearly described with a sample size that is excellent for a population genetic study.

(1) Section 0.2.1 The description of how singletons were handled was a bit confusing. If I understand correctly, two worms both had “inflated” singletons (how many singletons count as inflated?), which suggests sequencing errors, and were excluded. For the remaining worms, six sites contained singletons and were removed from downstream analyses as they are not informative about relatedness between individual worms.
Thank you for bringing up this point on clarity. We have updated this section to better describe how variants were identified, criteria for determining which variants are true signals versus sequencing noise, and retaining the true variants to inform population diversity.

(2) Section 0.2.2 The use of the term “untargeted” can be removed as it's confusing—the entire mitochondrial genome was used, not just the portions that were not amplified and sequenced, so just saying “complete mitochondrial genome” is sufficient. Were optical duplicate reads and reads that mapped to more than one location in the genome removed in the analysis? If not, then this would need to be done; if it is part of the pipeline, please state this explicitly.

Thank you for pointing out the confusing terminology. We intended to highlight the difference between the targeted amplicon method of the three loci and the shotgun NGS approach from the downloaded samples from Durrant et al. 2020. We have updated the title to “complete mitochondria genome” and clarified the shotgun portion in the methods for this section. Only deduplicated, uniquely mapped reads were used in this analysis and we have added the deduplicated and unique mapping specification to the pipeline description.

(3) Section 0.3 In the phylogenetic and population genetic research communities, UPGMA has been regarded for decades as a poor estimator of phylogenetic relatedness. Other methods are expected to be more accurate if the input data are sufficiently informative (maximum likelihood, Bayesian approaches, or even neighbor joining). For their phylogenetic analysis, the authors assume the simplest model of sequence evolution. This is an assumption that needs to be tested prior to running the analysis. There is no attempt to measure statistical support for the tree; in a maximum likelihood framework, bootstrapping can provide as a commonly used metric for statistical support, while in a Bayesian framework, posterior probabilities indicate the strength of support for a particular relationship. Finally, changes over time cannot be explored on the basis of this tree because the tree is not rooted, which is an essential component to directional analysis. Phylogenetic methods are used viral epidemiology because viruses evolve very quickly within a single host. This is not true for parasites. I suspect that a phylogenetic tree built using solid phylogenetic methods would end up being a polytomy (i.e., no strong support for relationships between haplotypes). As an alternative to a phylogenetic approach, I might recommend instead applying population genetic methods that examine differences/changes in allele frequencies (i.e., the proportion of haplotypes in a given year). For example, discriminant analysis of principal components (DAPC) has easy-to-follow tutorials available online (Jombart et al. 2010). This could also be applied to produce a lovely visual comparison of how human vs dog haplotypes cannot be discriminated. That said, my intent here is not to push one particular method, but rather, to encourage the authors to increase the sophistication of whatever method they do use so that their conclusions are robust and well-supported statistically.

Thank you for this very thoughtful comment pointing out the limitations of UPGMA. Sequenced samples predominantly spanned three years with a few exceptions. This is relatively short compared to the 10-14 month life cycle of a worm from infection to emergence and the low mutation rate of the mitochondrial genome. We first tried UPGMA given that it’s a simple model of evolution and thought it would sufficient to distinguish any samples that might be different due to importation events or geographic isolation. We also achieved similar results implementing a neighbor joining clustering method.

We now recognize the limitations to those approaches and have implemented DAPC as suggested by the referee. We agree that a population genetic method is more appropriate than even an improved phylogenetic tree algorithm. The results from DAPC support the previous conclusions that there is a significant overlap in genetic similarity between worms collected from humans and dogs. We have added a new methods section outlining this procedure and a new figure (S1 Figure). We again thank the reviewer for their suggestion.

(4) Section 0.5.2 The authors calculate an expected diversity in the sample based on the mutation rate/generation x the length of the mitochondrial genome. This is a mis-application of this kind of mutation rate—it is not a substitution rate. The mutations Denver et al. quantify occur during oogenesis/gamete development, and any expected diversity that could be calculated would be between a mother and her offspring. It can't be used simply to get an estimate of population diversity. The usefulness of the mutation rate estimate based on C. elegans for population genetics is that it can be used to parameterize coalescent models, which are extremely useful for a range of questions in population genetics and in some applications in phylogenetics.

Thank you for allowing us to clarify the goal of using this mutation rate to place our downstream choices in context. We used the mutation rate from C. elegans to approximate the number of variants in a barcode that could link direct transmission events (i.e. maternal and child pair) in the event of a spontaneous mutation. The extrapolation of acquiring less than one mutation per generation suggests two worms with a single base pair difference in the molecular barcodes are likely not connected by a recent lineage. We have revised section 0.5.2 to highlight our intended goal. We removed the statement equating an average number of variants per samples collected to the identity groupings since the expected mutations per generation is sufficient.

(5) Section 0.5.3 If I understand correctly, f is an average uncorrected pairwise distance between communities. This is a metric that has been previously described in the population genetic literature and should be cited.

Thank you for highlighting the inconsistent language. We have updated the method section language to be explicit that the pairwise geographical distance is the basis for the analyses and not a pairwise genetic distance. Sample pairs are grouped by exact identity or at least a single base pair difference, which differs from other genetic distance methods that account for degrees of differentiation within and between populations.

(6) Section 0.6 When comparing the diversity of COIII, cytb, and ND2-5 to other parts of the genome, there is no consideration of why variation may be higher in other regions of the genome based on genome annotation. The reason this is important is that some regions of the genome evolve differently than other regions. For example, regions that are non-coding (less of the mitochondrial genome than nuclear in animals) tend to have a higher diversity than coding regions. In contrast, some genes are expected to be under stronger selective constraints than

others. Genes that code for rRNA/tRNA tend to evolve differently than genes that code for proteins. Finally, primers for PCR-based studies are designed to bind to conserved regions to ensure robust and repeatable PCR across diverse samples. Thus, comparing the diversity as has been done here is not informative about future marker/barcode development. Rather than re-run these analyses taking into account how evolution is expected to act differently on variation in different parts of the mitochondrial genome, another option is to simply remove these analyses from the paper entirely. The take-home message is that sequencing more of the genome results in more information to distinguish worms and that some regions of the mitochondrial genome might be more variable than others, neither of which is novel or unexpected, and the same point could be made in the discussion by citing existing literature.

Thank you for this thoughtful response to this section. We agree that our results of finding more variants with a larger genomic range is not novel. We have framed the section to highlight the motivation for these analyses specifically for Guinea worm. Applying standard shotgun sequencing approaches is difficult in Guinea worm due to sample collection protocols that can result in degraded parasite DNA and microbial contamination.

The original loci (CO3, cytB and ND3-5) were targeted because of their variation and ability to amplify. It was unclear before our study whether incorporating more regions of the genome into analyses would justify a new protocol development, which requires time and financial support. In showing that the pairwise genetic similarity distributions differ when including additional coding and non-coding regions, it supports the recommendation for an improved protocol.

Reviewer #2: I find the methods confusing and not easy to follow. References are lacking for many of the methods used. It is also not clear as to how the Sanger sequences were checked and edited to rule out errors. The terminology needs to be reviewed in places to articulate what is an analysis term versus a biological term. For example "worm pairs" is used a lot and this must relate to between sample analyses rather than pairs of worms?

Thank you for bringing these points to our attention. We have added clarity to highlight that the measurements in our study are based on identity groupings correlated to true geographic distance, and genetic similarity is based on Hamming distances. We will refer to worm pairs as pairwise comparisons between worms to clear up terminology.

No ethics are detailed and as several of the samples are from humans this needs including in the methods. Also, anything related to live animals.

Thank you for this comment. The study uses national case surveillance data, de-identified case surveys, and extracted worms that are routinely collected from a confirmed case. We have added an “Ethics statement” section to the method section confirming extraction and data de-identification for human subjects.

Reviewer #3: Yes to all of the above except the last question. I see no ethical or regulatory issues.

Results
-Does the analysis presented match the analysis plan?
-Are the results clearly and completely presented?
-Are the figures (Tables, Images) of sufficient quality for clarity?

Reviewer #1: The results as presented are adequate.

Reviewer #2: I feel the results could be much clearer. There is some very good data but unless you have a modelling background then it is not easy to follow. The main outputs from this study will be of interest in terms of the biology of transmission and so the methods and results should be more tailored to that audience.
Thank you for this broad comment about readability. We have simplified language in the results section to improve clarity.

Sequence data should also be made available on a database such as Genbank.
The data been made available on Genbank and we have added accession numbers to the article under the new "Data Availability" section.

Reviewer #3: Figures, Supplementary Figures, and Supplementary Information:

Please see the marked up copy with specific suggestions. There are several typos within the legends and some suggestions (like the addition of scale bars to maps), that may improve the figures. Also, in the current formatting, I found the figures to be too small. For example, it was hard to see distribution of the dots in Figure 1a and 1c. In addition, the labels in S4 were hard to read.
We'd like to thank Reviewer 3 for their time and effort to provide thorough comments on grammar and typos that were missed during editing in the main manuscript and the S1 Appendix. We have updated the manuscript accordingly and improved figures where possible based on suggestions. Due to the close geographic proximity of samples in the study, increasing the size of the dots for each sample overpowered the ability to distinguish samples in certain regions in Figure 1. The replication of these geographic distributions in S6 Figure for individual barcodes helps counteract some of the limitations of capturing all samples in a single figure. Changing the landscape of S4 Figure to increase the host number labels did not fit page limits.

Conclusions
-Are the conclusions supported by the data presented?

-Are the limitations of analysis clearly described?
-Do the authors discuss how these data can be helpful to advance our understanding of the topic under study?
-Is public health relevance addressed?

Reviewer #1: The authors make a convincing case based on their analyses about the utility of parasite genetic data to inform strategies for elimination.

Reviewer #2: The conclusions are discussed together with the limitations but better structure is needed for this particularly as the limitations seem to far outweigh the conclusions.

Reviewer #3: Yes to all of the above.

Editorial and Data Presentation Modifications?
Use this section for editorial suggestions as well as relatively minor modifications of existing data that would enhance clarity. If the only modifications needed are minor and/or editorial, you may wish to recommend "Minor Revision" or "Accept".

Reviewer #1: (No Response)

Reviewer #2: See more detailed comments below

Reviewer #3:

Abstract
1) Background, consider hyphenating "ten-year absence".
The addition of a hyphen was to the abstract.

2) Methodology, specify "dog and human hosts" given the mention of paratenic hosts in the previous sentence.
The addition of "dog and human hosts" was added to the abstract.

3) Methodology, rephrase the last sentence to improve quality. A suggestion is in the attached manuscript.
Thank you for the recommendation. Sentence has been updated in the manuscript.

4) Methodology, replace "three loci on" with "three loci in"
The word change has been updated in manuscript.

5) Consider revising the last sentence to improve clarity. A suggestion is in the attached manuscript. Also, this part of the abstract makes the least sense as there is no reference to "worm

pairs” in earlier parts of the abstract. More of a transition to this part of the study in the Abstract would improve clarity.
Thank you for the suggestion for the last sentence. We added the motivation for the complete genome analyses as a transition for the results.

6) Conclusions/Significance, revising a sentence will improve clarity – see suggestion in the attached manuscript.
Thank you for the suggestion. The sentence has been updated in the manuscript.

Introduction
1) Line 8, specific that these losses are due to Dracunculiasis
Added “losses due to Dracunculiasis” to this sentence.

2) Line 10, hyphenate “decade-long”
The addition of a hyphen was to the introduction.

3) Line 27, hyphenate “genome-wide”
The addition of a hyphen was to the introduction.

4) Line 38, delete “directly”. Using genetics to track the movement of parasites is an indirect way of assessing movement. You are making inferences which is based on host sample size, parasite sample size, variability of loci and number of loci, etc.
Thank you for pointing out this distinction. We have changed “directly” to “indirectly” in this sentence.

5) Line 43, improve clarity by revising sentence.

Methods and Materials
1) Line 69-71, clarify what “samples overlapped” means – the individual worm was part of the previous study? Also, delete extra “.”
The sample overlap was in reference to samples that were a part of a previous study. We have updated the language to make this clearer.

2) Line 77-78 add the unit “bp” behind each reported range as the current formatting initially caused confusion.
The updated the sentence to specify genomic range in the manuscript.

3) Line 82, was comparing singleton counts among sequences the only method of quality control? Given that Sanger sequencing was used, were chromatograms examined for the sequences that were excluded?

Thank you for allowing us to clarify this point. Consensus sequences from chromatograms were obtained as previously described in Thiele et al 2018, referenced in the previous section. We did

have a few (4) samples with ambiguous calls, now mentioned in the section. These sequences were then aligned to the reference genome to confirm the correct sequence was targeted. The variants that were identified once in the population were checked against the worm that harbored the variant. Two worms with 50% of the singleton variants identified in the population in separate loci were excluded from variant discovery and analyses.

4) Can line 83 ("Singleton variants identified in these two samples were excluded") be deleted if these two sample were excluded (see previous line)?
Simplified with the above clarification.

5) Line 84-85. Why was the position missing a base call? Was this a quality issue or a deletion or insertion?
Thank you for bringing up this point about our definition of missing. The ranges in this study were defined by the alignment ranges of all samples. A missing base call is due to technical variation of amplification and fall at the start and end of ranges. We have updated this section with this detail, with clarification that positions with a missing base or an ambiguous call in more than 2 samples were not considered candidates for the molecular barcode. Indels were not observed otherwise.

6) Line 90, revise the subheading to help remind readers why this part is included in the study.
We have added the motivation for this section in the results.

7) Line 92, revise this sentence. A suggestion is provided in the attached manuscript.
Thank you for the suggestion. "Identified as Dracunculus" was added to this sentence.

8) Line 149, it is not clear what constitutes a "data-driving, empirical form".
Thank you for pointing out that this was unclear. This sentence has been updated to be direct that the function form is derived from the pairwise geographic distance between identical and non-identical barcodes.

9) Line 179, what does this symbol mean, or is this a requirement of the journal? Replace with "see section 0.5.4?" Do this in other places is applicable.
The symbol has been updated to "see section" throughout the manuscript.

10) Line 203, clarify what "population worm cases" are. How does one go from 459 to 425 worms?
We have updated "population worm cases" to be clearer with "worm samples with matched coordinate data". The drop out of samples from the genetic comparison to the spatial analyses is described in Section 0.7.

11) Line 217, add a unit here to clarify that this is bp. Also, is the reference to PlasmoDB correct? If so, more explanation is needed.

Thank you for catching this reference error. The gene coordinates were pulled from ENA refence genome used for alignment. We have corrected this in the manuscript.

12) Line 238-252, the methods associated with the barcode accumulation curve analysis should be included in the Methods section rather than the Results section to foreshadow the result.
Thank you for the suggestion. We believe these accumulation curves help guide our recommendation to sequence all available worms but constitute a separate section to remain as the supplement. We have added a reference to the accumulation curve supplemental material in the methods section 0.3.1 to preface the methods prior to the results.

13) Reorder the methods to follow the order of the Results section. Put 0.2.2 last.
Thank you for the suggestion. While moving the methods for the complete mitochondrial DNA section does flow with the results, we paired "Alignment and variant identification" into a single section to ensure that the differences between calling variants were easy to compare.

Discussion

1) Line 357, expand on why it is important to recognize diversity – especially this type of genetic diversity "mtDNA) in the discussion.

Thank you for bringing this to our attention. This sentence was meant as a precaution for imposing interventions in boundaries identified from this study might not be sufficient for barcodes that have a broader range. We have rewritten this sentence to be more explicit on this point.

2) Line 396, replace "lead" with "led"

This typo has been corrected in the manuscript.

3) Line 404, move "other" to in front of "parasites".

The placement of "other" was updated in this sentence.

4) As someone who works on non-model parasites, I was curious as to what constitutes "a tailored set of phylogenetic or phylodynamic methodologies".

Thank you for allowing us to clarify this point. This sentence was a preface the unknown mutation rate to build time-corrected phylogenies. We have removed this sentence from the manuscript to eliminate redundancy.

5) Line 423, What is "between resolution". Consider revising as "better resolution of genetic similarity between Guinea worms in Chad. Why would this only improve the resolution in Chad? Wouldn't it improve it analyses of genetic similarity in other countries too?

We have updated our manuscript to improve the idea of better resolution. We mean that there are different distributions of genetic similarity, not a shift in the distribution, suggesting more sites

will change how samples are related. We agree more sites would improve any Guinea worm analyses in any country from principle and have removed “in Chad” from this statement.

Summary and General Comments
Use this section to provide overall comments, discuss strengths/weaknesses of the study, novelty, significance, general execution and scholarship. You may also include additional comments for the author, including concerns about dual publication, research ethics, or publication ethics. If requesting major revision, please articulate the new experiments that are needed.

Reviewer #1: In this paper, the authors use an impressive number of samples from Chad to explore the relationships of genetic variation to geographic variation with the goal of building a model that can be used to estimate the risk of recrudescence should apparent elimination of Guinea worm in humans be achieved. This is an admirable and excellent goal, and the introduction and discussion are well-written and make an excellent argument for genetic epidemiology as a valuable approach. The authors convincingly build a case that identifying correlations between genetic diversity and geographic distance has practical applications for elimination of parasites. This research is a good first step.

However, the paper lacks diversity in citations of other literature on population genetics of parasites, primarily relying on previously published work on Guinea worms, and this is reflected in some problematic aspects of their analysis. There are some analyses that would need careful attention before I would recommend publication of the manuscript as-is. In particular, the methods used to generate the phylogenetic tree are inadequate, and the data need to be re-analyzed or replaced with a population genetic approach for answering the same questions (i.e., regarding dog and human sample clustering and changes in genetic diversity over time); the exploration of genetic diversity in the mitochondrial genome as a whole will need additional analysis to add more depth, or could be removed entirely.

Reviewer #2: This is an important study that aims to investigate the transmission dynamics of Guinea Worm to support eradication efforts. There is a large dataset analysed providing important and novel data, however the paper lacks clarity and is hard to follow with the key points lost within the analyses. Details are shown below and I suggest the authors revise the paper to make it more clear to the non-modelling audience.

Abstract
- worm pairs ? do you mean samples or worm pairs.
We have updated all instances of “worm pairs” to “pairs of worms” in the manuscript.

Line 16: re-phrase the line. Genetic sequencing is not a tool for control but supports control through the understanding of transmission. Also change “Genetic sequencing” to “genetic analysis”. Sequencing is just one of many methods.

“Sequencing” was updated to “analysis” in the manuscript.

Methods
A section on sample information is needed. E.g. what were the hosts for the 712 worms ? Dates of collections etc. A supplementary table would be useful.

We include the year and host for the 459 worms that were successfully sequenced at three loci and included in the study as summary Figure 1A. Specific details about cases, such as dates tied to names, need approval from the CGWEP .

Lines 66-71 – make it more clear about the data from the samples as it is currently confusing. Maybe add a table of what DNA regions are available for each region. Also, was the data from reference 10 used in the analysis or was new data obtained from the samples used both studies. If the data was used then accession numbers need to be included etc. Make this much more clear. Add more information about how the data was obtained and edited. It is in reference 10 but good also to include some basic information and not just the reference. Include the loci etc. In the introduction it should also be stated that you are building on the data already analysed in reference 10.
The 459 samples included in variant discovery had successful sequencing at the CO3, cytB, and ND3-5 loci as mentioned in Methods 0.3.1. We have included the accession numbers for all previously published and new samples in this study user the “Data Availability” section. We mention the inclusion of previously published samples in the concluding paragraph of the introduction.

Line 74-89 – A typical sanger sequencing approach that the data was generated by allows for sequence editing and checking using sequence chromatograms. Can you confirm that this was done for the sequence data prior to alignment and allow you to produce contigs for each sample which can then be analysed. The methods described are confusing e.g. what do the genomic ranges relate too ? why is there missing data ? missing data would be due to sequence error and not as a variant ? you would not normally see deletions within a species ? What do you mean by singletons and why are some discarded and some kept ? I am also assuming that your barcode gives you a genotype so you identified 41 genotypes within the 459 samples ? It might also be worth investigating the SNP’s in terms of synonymous versus non-synonymous mutations. Was there also clear differences in the frequency of the different genotypes.

Thank you for this thoughtful comment. We have expanded the analyses to summarize calling methods from chromatograms as described in Thiele et al 2018. The ranges refer to the genomic location on the mitochondrial genome that the reads spanned after alignment to the reference genome. Missing positions are driven by amplification and alignment variation at the start and end of the which were identified from the population. We had a few samples (4) that had ambiguous base calls which we have now mentioned in this section. We were previously considering any non-ATCG base as “missing” but has been updated more details. With the described exclusion of positions with more than two missing or ambiguous positions as

candidates, we eliminate the variable start and end site positions in the molecular barcode. We do not observe indels in other regions of the gene.

We also added details of how true singleton variants were distinguished from noisy variants; six true variants were retained in the molecular barcode. The barcode is a genotype representation of variants identified these three loci.
We agree there is interesting biology regarding the type of mutations identified, however for modeling groups of identical and non-identical barcodes, we would treat a variant in either mutation class similarly. If I understood correctly about genotype meaning molecular barcode from this comment, there are differences in the frequency of barcodes. The frequency of different barcodes by host species is provided in S1 Table.

Lines 90 109 – so you need to explain that this is NGS data as this is annotated and aligned in a different way to the sanger data, particularly in the way that errors and quality is assessed. This data also needed to be included in some sort of sample table showing what data comes from what sample and what methods etc. It is unclear if the whole mt genome was used in the analyses or just the target genes.

Thank you for allowing us to clarify the sequencing methods. The information for these samples can be found in the ENA accession metadata for the Durrant et al. 2020 for mitochondria samples from Chad. We have provided a link to the Git repository with the sample accession numbers to the "Data Availability" section. These samples were not included in the construction of the molecular barcode and spatial analyses; they were independently assessed to investigate the value of additional markers in Guinea worm. The complete genome was used in the analyses regarding variant discovery and pairwise genetic similarity of the population. We compared the pairwise genetic similarity calculated from variants found in the amplified genes, outside the amplified genes, and all variants.

It is not clear why the barcode method is better than a full sequence analysis, with model selection, bootstrapping, AA changes etc.
Following a recommendation from Reviewer 1, we have dropped the phylogenetic analyses from the supplement and pivoted to another method that achieves the goal of barcode comparison between hosts.

Line 131 – this GPS data should also be added to the sample master file so the full data is available.
Thank you for this point. The GPS data are protected under data sharing agreements by the country of Chad. We will provide a matrix of the geographic distance between pairs that are necessary for the spatial correlations to be transparent within specified limits.

Line 139 – refer to a table that details to number of worms analysed per host and what the hosts were.

We specify 65 of the 245 hosts in this study had multiple worms in Results Section 0.10.1. The hosts with multiple worms are plotted in S4 Fig for reference.

Line 144 -explain what you mean by yearly transmission cycle. Is it to do with pre-patent periods or seasonality etc.
Thank you for the clarification on the yearly transmission cycle. We updated this sentence to be clearer about the yearlong pre-patent period of Guinea worm infection.

Spatial Analysis – would the spatial analysis be based on water bodies that are connected to allow spread not just distance between water bodies that allow movement of hosts between water bodies.
This is an excellent point based on the transmission mode of Guinea worm. We only have information on the village coordinates upon case confirmation. We do not have enough epidemiological information about movement of humans and dogs to infer the body of water that may have caused the infection.

Line 169 – you refer to sequencing error here. Sequencing error should be very low for the Sanger data so it is confusing as to why more sequence editing was not done. Also, could a genetic clustering analysis (e.g. a haplotype network or similar helps with grouping population clusters which could then have been analysed spatially. Also was the clustering analysed in terms of host ?

Thank you for bringing up this point. Sequencing error should be very low for Sanger sequencing. DNA degradation can be an issue in Guinea worm. The sequences were aligned to the reference genome to confirm we had targeted the correct regions for each worm across all loci. No sequences were edited in this study, but ambiguous calls were checked. Given the short time scale for the analyses over a few years in relation to the expected mutation rate in terms of lineage identity, having 100% identity is ideal for transmission tracking within the limitations of mitochondrial DNA. In sensitivity analyses, we allowed for a single base pair difference to be considered for identity to account for potential sequence errors (despite an expectation for this to be low) and/or a potential underestimation of the mutation rate that is unknown in Guinea worm.

Analyses for human and dog hosts were computed separately but not included in this study. There are few human host samples in this study; we are not adequately powered to perform the analyses on humans between identical and non-identical barcodes. Dog only analyses mirrored the analyses presented in the manuscript because they dominate the sample population. Please see the results below for dogs only.

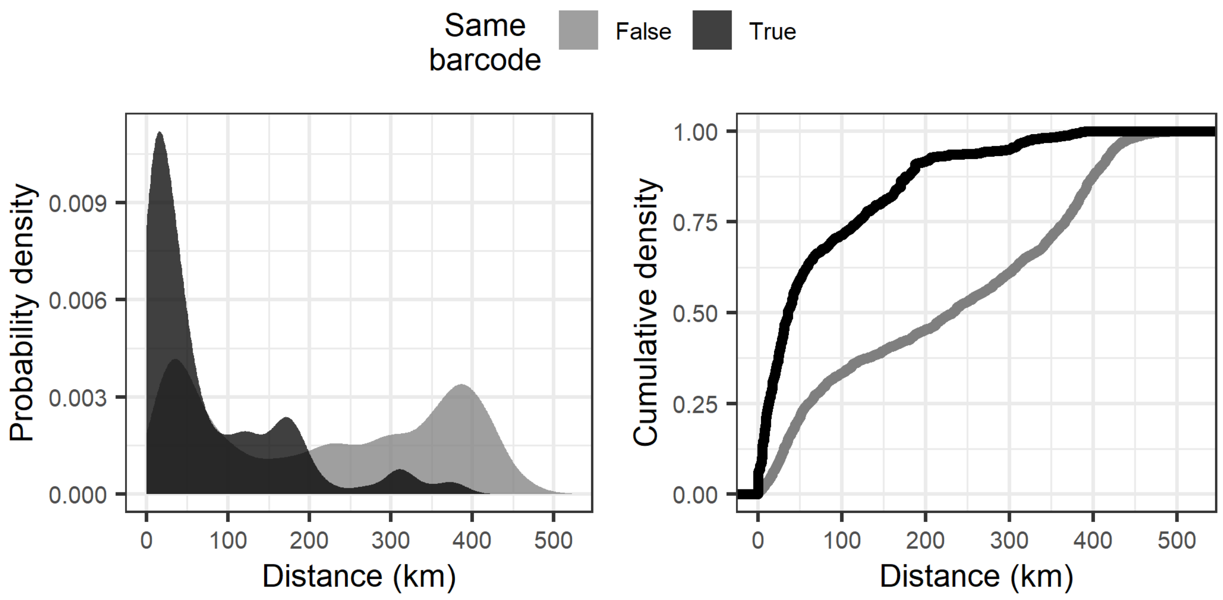

Line 194 – refrain from using the term worm pairs as this suggests male and female pairs. Use a different terminology.
Thank you for bringing up this terminology distinction. We have updated instances of "worm pairs" to "pairs of worms" throughout the manuscript.

Sensitivity analyses – is this the correct term or should it be statistical analyses.
Thank you for this point. While all the methods in the paper are statistical analyses, the sensitivity analyses are those that test the assumptions of our barcode grouping and other epidemiological features that may confound results.

What does distance vector relate to – is it the disease vector (copepod). Do try and help the readers that are not modellers.
Thank you for bringing up this point. We have changed that sentence to be clear that the distance vector is the all-by-all pairwise geographic distances.

Paragraph 202 – are there references to these methods used. You also need to be more clear on how this is done and refrain from complexed terminology e.g. what is the index worm and are the population worm cases just samples ?
Thank you for highlighting the complexity of this section as originally written. We have rewritten this section to remove specific terminology and reference that a technique is applied to each worm in the study.

Paragraph 214 – more references need to be included for bit of the analyses used. It is difficult to understand what data the variants outside of the loci is based on ?

Thank you for allowing us to clarify this point. We have expanded the section to clarify that variants outside loci are coding and non-coding regions that are not in the CO3, cytB, and ND3-5 loci used to characterize Guinea worm in the spatial analyses of the study.

Line 232 – Figure 1A says number of sequences per host for each year but this should be number of samples analysed there will be more than one sequence for each sample?
We have updated the figure to reflect the number or sequenced samples per year.

Line 234 – case coordinates means geographical location of the infection ? make this more clear.
Case coordinates refers to the geographical location the reported case after emergence; the location of the infection is unknown. This sentence was updated for clarity.

Line 241 – this Binomial analyses should be detailed in the methods ?
We have added a reference to the appendix containing the binomial analyses to the methods section 0.3.1.

Line 267 – the analyses are based on mitochondrial DNA so diversity will be high and increase with sample size as seen in the dogs. Based on the mt DNA you do not know if the worms are genetically identical, they are the same genotype based on maternal inheritance but you cannot tell if they are clones ?
Thank you for bringing up this excellent point. We do not know if worms in a pair are genetically identical beyond the variants identified in the sampled population at these positions. Will update to state identical barcodes.

Line 278 – what is a sequencing mutation ? do you mean error ?
We have updated "mutation" to "error" in the manuscript.

Line 309 – this is the information that was needed in the methods.
This information is replicated in the methods.

Figure S4 – add better scale to the A – is the lower limit 1 – also the last host has lots of worms – this kind of extreme should note some discussion. The Y axis numbers are also unclear. For B use yes and no instead of false and true as false tends to mean unreliable.
Thank you for the suggestions to improve our figure. We have specified plot legend the graph only contains hosts with more than one infection. The Y axis is the number of sequenced worms collected per host. Figure B has been updated from True/False to No/Yes.

Figure S6 – you have non common barcodes shown too so change the figure title.
Thank you for catching the not common barcodes exclusion in the title. We have updated the title to specify the non-common barcode geographies also.

Line 326 – do you mean single variants – variants found in only one worm suggests you get variation within the worm ?

We have updated this sentence to be clearer that we are repeating the analyses without variants identified in a single worm in the population.

Line 327 – I think you need to make it more clear what you mean by within and outside loci.
The distinction for with and outside loci have been added to method section 0.6.

Line 343 – you did not analyse 459 sequences but 459 samples ?
This sentence has been updated to samples instead of sequences.

Line 345- is quantitative bounds the correct term ? do you mean boundaries ?
Bounds has been updated to "boundaries" in the manuscript.

In your discussion on limitations why was a full phylogenetic analysis not performed on the data and it was just based on the mutation barcode – this could have provided resolution to the analyses.
Due to the low mutation rate of the mitochondrial genome, we originally chose to build a tree from the variants due to partial clonality of the molecular barcodes. However, we have pivoted to a better population genetics approach to compare the barcodes between host species and have removed the phylogenetic analyses from the supplement.

Also data should also be made available through a data base such as Genbank.
We have uploaded the sequences to Genbank and list the accession numbers in the new 'Data Availability' section.

I think the discussion could be tightened to show the clear findings better. There are clearly many limitations to the study and these are discussed but I do not find the discussion well structured.

Thank you for this suggestion. We balance the limitations of the marker set used to characterize the population with sensitivity analyses to support the conclusions while providing evidence for other sequencing technologies. We believe the current structure will allow individuals that guide programmatic decisions to evaluate the strengths and current limitations of genomic surveillance in Guinea worm.

Generally the paper shows important findings and advances the needed analyses for understanding the animal reservoirs for or the zoonotic transmission of Guinea Worm but the paper is very hard to follow, with non-uniform terminology and also terms that mean several things e.g. could relate to the biology of the parasite or the analyses. The authors need to consider the audience and provide clearer methods and results.
Thank you for this comment. We have updated various portions of the paper for clarity to address inconsistent terminology between fields and simplify methods to improve readability.

Reviewer #3: This manuscript builds upon previous molecular-ecology approaches to better understand the distribution of genetic diversity of Dracunculiasis in Chad. The dataset is expanded by considering more loci and more samples in space and time. The results show that worms with the same mtDNA multi-locus barcode tend to be clustered within a relatively small geographic range (within 50 km). However, these same barcodes are not partitioned by year or by host suggesting some fluid transmission dynamics. These findings are the result of comprehensive analyses and complimented by a further exploration of what these patterns would be like if the entire genome was analyzed. Perhaps unsurprisingly, modelling with genomes suggested that more loci would give better resolution into the genetic relationships among samples and would improve the resolution that genetic data could provide for programmatic surveillance and decision-making.
This manuscript stands out for its comprehensive set of analyses to investigate the validity of its various results. Also, I enjoyed reading the Discussion section. I recommend publication pending minor revisions. I included a list of these comments, but also copies of the MS and the supplemental materials with my comments annotated throughout.

Editor Revision Round 2

I have flagged this in the system as “major revision”, but that might be excessive. Some specifics. But you need to read the entire paper for flow and consistency. It remains verbose and could benefit from skilled editing.

I’ll refer to line numbers in the document PNTD-D-20-01771_R1_trackedChanges.pdf.

We’d like to thank the editors for guiding further improvements of our paper before sending for a second round of review. The new tracked changes document, which includes many of the recommendations below, is in reference to the original submission.

Abstract (no lines numbers for the abstract in the document).

Wrong use of the word “paratenic”

Thank you for highlighting the use of paratenic in this sentence. The rise in dog cases suggested that there may be another paratenic host (e.g. fish or frogs) that are facilitating transmission.

Are you sure you mean “…genetically identical worm populations…”?

Thank you for this comment. Genetically identical worm populations refers to worms that share an identical molecular barcode. We have included ‘genetically identical’ after the description of genotypes to clarify that identity is defined by the genotypes from the molecular barcode.

The sentence “we investigated the value of…” should be reviewed. And it is hardly surprising that the addition of a longer region of the mt genome will distinguish more lineages.

We agree that capturing larger portion of the genome will uncover more combinatorial variants to define a lineage. The main manuscript was expanded to include the motivation for this work; it supports the value of protocol development to include more markers than a novel insight. We have updated this section of the abstract to include “as expected” and the protocol development.

In the third line of the “conclusions/significance” section of the abstract, is “co-occurrence” better than “overlap”? I have trouble envisaging two worms overlapping.

Overlap changed to co-occurrence.

Line 23. You talk about “phylogenetic methodologies…”. But the phylogeny previously in the paper has been removed. Do you want to retain this part of the introduction?

Thank you for highlighting this point. While the phylogenetic analysis is not relevant for our point of tracking recent transmission, this referenced literature does highlight the relatedness of worms in Chad on larger time scales.

Line 66. Something is missing after “successfully”. Should it be “amplified”? “sequenced”?

Added amplified and sequenced to this sentence.

Line 70. Maybe “reported in previous literature”?

Updated “included in” to “reported in”

Lines 75, 98 and elsewhere. The adjectival form “mitochondrial” would be better.

Mitochodria update to mitochondrial in the manuscript.

Senetence starting on line 77. Is it needed? The sentence that follows seems to provide necessary information.

Line 78. Phrasing. I would say “The regions sequenced were from sites 3,690 to 4,308 for CO3…etc.” See also around lines 250-253.

Sentence updated to remove introduction sentence and specify genomic ranges.

Lines 84-93. Here, the attempt to clarify the way in which singletons were dealt with has not made things much clearer. Please revisit this section. I quite like “inflated singleton counts”, which you have removed in this revision. The next few points also relate to this section.

Thank you for highlighting this point. We expanded this section to provide further detail on candidate variant positions and included “inflated singletons” and their definition in the explanation.

Line 86. The sentence starting “Non-singleton variants were retained if…” confuses me. If a base (variant?) is present in "all but one sample", then surely that one sample represents a singleton?

Line 87. The sentence starting “Four samples had an other position…” does not make much sense.

Thank you for bringing up these points on lines 86 and 87. We have expanded on the details of this section to specify an “other” designation at a position (true missing due to amplication variation at start and end of gene ranges, or an ambiguous call). The “all but one sample” was referring to additional filtering of candidate variant positions after the removal of inflated singletons where a sample had more than one missing base or ambiguous call at that position. We allowed for two samples in the population have the designation of other to allow for both a missing and ambiguous call at one site, which has been corrected in the manuscript. This allowed us to retain informative variant positions in the barcode for the population while allowing some flexibility for technical variation in a minority of samples.

We coded ambiguous and missing bases similarly in the barcode and have corrected that the “missing” bases are ambiguous base calls and not deletions from the Sanger reads. We updated the sentence describing that 4 samples had an ambiguous call in a position in the barcode that represent 3 barcodes in the study. This was the highlight that the barcodes does not contain missing positions, and a small fraction of samples (<1%) were affected by technical variation.

Line 89. “These criteria”. Given the new material added to the paragraph, the reader might have trouble working out what criteria these are.

We removed “These criteria” from the revision because the sentences describing the positions, removal of inflated variants, and allowing for the inclusion on an ambiguous base is described.

Line 92. Remove “see”.

Removed additional word from sentence.

Line 95. Surely it is not Dracunculus (note spelling and lack of italics) samples that were publicly available, but rather Dracunculus mitochondrial genomes?

Thank you for this correction, it is Guinea worm mitochondrial sequences. The sentence has been updated.

Line 126. Rather than “of the molecular barcodes” I would say “based on the molecular…”

Updated to “based on the molecular…”

Line 131. Should the word not be “discriminant”?

Updated discriminate to discriminant.

Line 159. This sentence needs attention. I don’t think you can refer to a “pre-patent cycle”. Maybe something like “Here, given the annual life-cycle of guinea worm, …”

Thank you for this point. We followed a suggestion from reviewer 2 to clarify that the prepatent cycle is indeed the correct terminology since we are specifying the time between host infection and skin breakthrough by the adult female worm to release eggs.

Line 164. “…is a the…”?

Extra word “a” removed from the sentence.

Line 170 and elsewhere. How often do you need to say “the functional form of f”? I found the phrase at least three times. And should “f” be in italics or not? Be consistent.

Thank you for noticing this repetition. We have removed the extra instances of this phrase and removed the italicization of f to be consistent.

Lines 175-186. Present and past tenses both used. Reporting what you did, the past tense is better. This switching between tenses is throughout the Methods and Results sections. Please check and be consistent. Results should also be in the past tense: reporting what you actually did.

Thank you for pointing the tenses out to us. The manuscript has been updated to the past tense in the Method and Results sections. Additional updates in the

Line 205. The sentence starting “Note” is hard to follow.

The sentence has been rewritten for clarity.

Line 214. “criteria” is a plural form.

Updated criteria to criterion.

Line 222 “worms pairs”?

Worm pairs updated to pairs of worms.

Line 238.”The number … was counted…”

Were updated to was.

Lines 247-256. Revisit and try to increase brevity and clarity.

Thank you for highlighting the length of this section. We have rewritten this section.

Lines 2720273. “…for each reported case”?

“The” updated with “each” in this sentence.

Line 273. The map in Fig. 1 does not identify the Chari River.

Thank you for highlighting this point. We will continue to search for river shape files that meet the journal’s attribution requirements.

Line 360. I would replace “with” with “using”.

“With” updated to “using”.

Lines 399-403. Some of this seems to be stated also around lines 377-379. Check for repetition.

Thank you highlighting this repetition. The second instance of describing the distribution of pairwise similarity is the sensitivity analyses excluding singleton variants that held the same trend when including variants. We have added the new figure after the sentence to clarify that sentence that is does refer to a different analysis.

Line 413. Maybe “Knowing the geographical range of transmission”?

Added geographical to the original sentence.

Line 436. Maybe “…and this suggests…”?

This added to the original sentence.

Line 516. “Sequencing more historical samples could either decrease genetic diversity…”. I don’t follow this.

Thank you for bringing up this point. We have updated this sentence to highlight that sequencing more worm samples could either help with sample size estimation or support the need to sequence all worms.

Lines 521-524. This is an example of a very wordy sentence. Try to edit it (and others) to a more digestible form.

Thank you for highlighting a wordy sentence as an example. We have tried to edit similarly structured sentences with this guidance.

Fig 2B, caption. Would better phrasing be “For each barcode, pairwise distances among all pairs of worms with that barcode were calculated“?

Thank you for this suggestion. This figure does show the pairwise geographic distances on a per barcodes basis. The original caption specifies that each molecular barcode has a different number of pairwise comparisons used in the estimate due to uneven representation of barcodes in the study, as provided for the population analyses.

Fig. 3, caption. Maybe “… does not drive apparent spatial connectedness”?

Apparent added to caption title.

Click here to access/download
Revised Article with Changes Highlighted
GuineaWormPhylodynamics_20210409_trackedChanges.pdf
