# Supplementary material for: Linked surveillance and genetic data uncovers programmatically relevant geographic scale of Guinea worm transmission in Chad
Source: PLoS Negl Trop Dis. 2021 Jul 26;15(7):e0009609. doi: 10.1371/journal.pntd.0009609 (PMC8341693; doi:10.1371/journal.pntd.0009609)
Supplement: Attachment — Submitted filename: GuineaWormPhylodynamics_ReviewerComments_202106.pdf [file pntd.0009609.s019.pdf]

Dear Dr. Ribado,

Thank you very much for submitting your manuscript "Linked surveillance and genetic data uncovers programmatically relevant geographic scale of Guinea worm transmission in Chad" for consideration at PLOS Neglected Tropical Diseases. As with all papers reviewed by the journal, your manuscript was reviewed by members of the editorial board and by several independent reviewers. The reviewers appreciated the attention to an important topic. Based on the reviews, we are likely to accept this manuscript for publication, providing that you modify the manuscript according to the review recommendations.

The paper has greatly improved, but the reviewers still noted some points they wished to see improved. The English could be better in many places. Note also comments from reviewer #3 about use of the term “loci”.

A few more details that I noticed:

Line 105. This sentence states that “Trained program and ministry staff … are stored in ethanol…”.

Corrected.

Lines 150 and 154 “mitochondrial genome”.

Corrected.

Line 163. Can a mitochondrial genome exhibit heterozygosity?

Thank you for this point. We can obtain heterozygous calls in a sample from heteroplasmy or potential nuclear mitochondrial DNA segments (unknown for Guinea worm, but biologically possible). Edited the sentence to highlight heterozygous calls likely from heteroplasmy.

Line 198. “… yearly prepatent cycle…”? Reconsider this phrasing. The life cycle is annual.

Prepatent updated to life cycle.

Various places. “barcode x” sometimes starts with a capital letter, sometimes not. Be consistent.

Thank you for pointing this out. Barcode has been converted to lowercase in the manuscript.

Line 364. Is 47 the correct number? Elsewhere 86 was stated.

Thank you for bringing up this point. The value of information analyses were conducted in an independent population with a different sequencing technology. The barcodes are comprised of the variants identified in each population. For the mtDNA targeted amplification, 86 variants were identified in the 459 samples, while 47 variants were identified in the smaller set of 19 samples.

Line 386. "… this robust trend was also observed…".

Updated this sentence to be clear about the trend of a broader distribution.

Line 391. "Variant calls…".

Fixed to "Variants called".

Reviewer's Responses to Questions

Key Review Criteria Required for Acceptance?
As you describe the new analyses required for acceptance, please consider the following:

Methods
-Are the objectives of the study clearly articulated with a clear testable hypothesis stated?
-Is the study design appropriate to address the stated objectives?
-Is the population clearly described and appropriate for the hypothesis being tested?
-Is the sample size sufficient to ensure adequate power to address the hypothesis being tested?
-Were correct statistical analysis used to support conclusions?
-Are there concerns about ethical or regulatory requirements being met?

Reviewer #1: The new analyses and clarifications have significantly improved the manuscript.

Section 0.4. Typically, cross-validation ("xval") is used to determine the optimal number of principal components to use in a DAPC. The approach used here, testing several different values for the number of PCs used in the analysis and reporting the qualitative similarities across replicates, is okay, depending on the distribution of variation in those PCs. The PC eigenvalues can be plotted relatively easily (scree.pca=TRUE). If the PCA eigenvalues reach a plateau by 5 PCs, then the number used should be acceptable. If not, then cross-validation will need to be used to determine how many PCs to include in the analysis.

Thank you for bringing up this important point. We used the xval procedure to identify the number of PCs recommended from 1 to 41 (number of unique barcodes), which suggested 15. We also evaluated the eigenvalues for each PC; we utilized an elbow method common in principal component analysis and singular value decomposition. We observed a significant decrease of contributed variance from four to five PCs. Due to the small sample size of unique barcodes in this study, we checked the suggested training set percentage (90%) was ideal for the cross-validation procedure estimates compared to 70% and 80% training set percentages. We have included the cross-validation results for the training set percent ranges, which do not show a clear arc for optimal components.

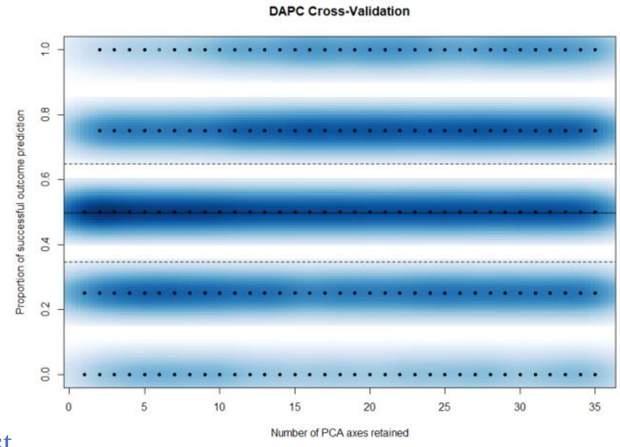

90% training, 10% test

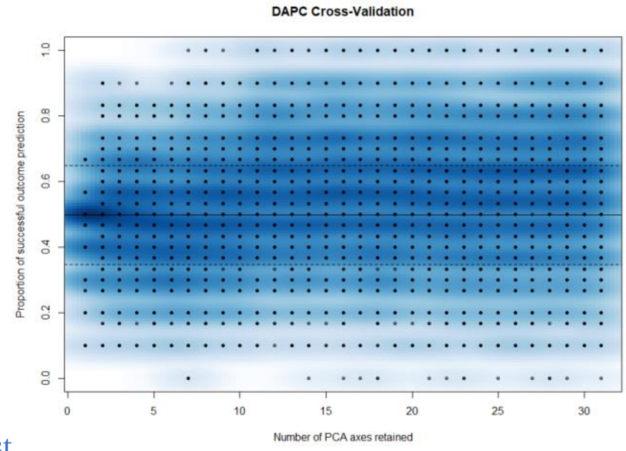

80% training; 20% test

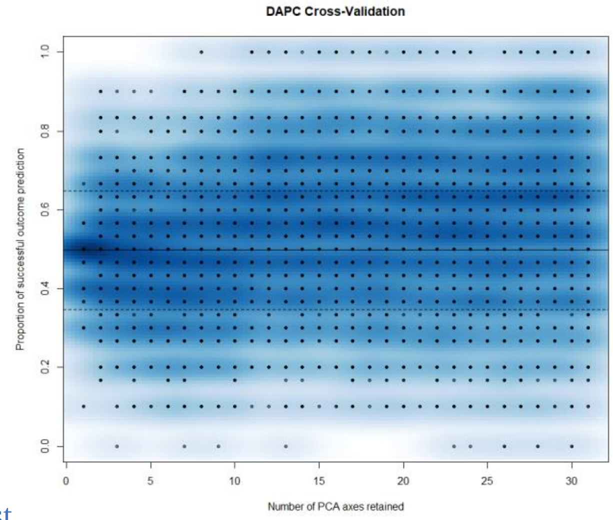

70% training, 30% test

We have updated S1Fig, methods, and the results section to include the component eigenvalue and cross-validation results.

Section 0.3.2. It is fairly standard to take the intersection of variant calls between alternative calling programs. I don't want to suggest re-doing analyses at this point, but if the barcode variants reported in the main text are found in both pipelines, it would be sufficient to indicate this rather than reporting variant distributions (lines 390-393/Fig S9).

Thank you for this comment; we did check the overlap of both pipelines; all but one variant in the GATK set was in the bcftools set. We included the number of variants (137) that had identical ref/alt genotype calls across the population; in 38 instances, bcftools had a call with one less alternative allele compared to GATK. We hope the inclusion of this detail highlights that the pairwise differences between samples are robust to variant callers.

Reviewer #2: The methods need more information on the samples used and to make clear what data is used from published datasets and what is new data as part of this study.

A table with the data specifications has been provided with the manuscript.

Reviewer #3: The revision of PNTD-D-20-01771_R2 is improved from the previous version. I have no major concerns about the methods used in this manuscript.

Results
-Does the analysis presented match the analysis plan?
-Are the results clearly and completely presented?
-Are the figures (Tables, Images) of sufficient quality for clarity?

Reviewer #1: The results and figures are acceptable.

Section 0.11/Figure 4. I am still not convinced that comparing the variants identified within loci (n=47) to the variants outside of loci (n=129) is an appropriate comparison given the limited sampling. The analysis does not discriminate between improved differentiation based on increased information (= more data) and improved differentiation based on different processes acting on variants located in these different categories. From the perspective of marker development, the comparison on the basis of whether a variant falls within or outside of the barcode used in the broader study is not useful. Comparing the 47 variants used on the larger sample to variants inferred from the whole genome is sufficient to make the point that increasing the amount of sequence data increases the information content.

Thank you for this excellent point given our limited sample size. Due to the low sample size of worms with whole mitochondrial DNA sequencing and their independence from the targeted

gene set, we cannot check for lineage differentiation improvements as mentioned in the discussion. To address your concern, we have removed this statement from the introduction to be more direct about the pairwise genetic distribution changes and potential for increased lineage resolution. This value of information analysis advocates for a tiled amplicon design across the complete genome instead of specific regions that may be undergoing different selection processes. Our analyses show that the rest of the genome would give more variants, as expected, but additionally those variants differ between pairs of worms to alter the distribution of relatedness.

Panel B of Fig 4 could be moved to supplemental, as it is not referred to in the discussion.
We have added a reference to Figure 4 in the main manuscript where this panel is called in the results section.

Reviewer #2: (No Response)

Reviewer #3: The revision of PNTD-D-20-01771_R2 is improved from the previous version. I have no major concerns about the results in this manuscript.

Conclusions
-Are the conclusions supported by the data presented?
-Are the limitations of analysis clearly described?
-Do the authors discuss how these data can be helpful to advance our understanding of the topic under study?
-Is public health relevance addressed?

Reviewer #1: The conclusions are well-supported by the data and limitations are discussed appropriately. This manuscript demonstrates that parasite genetic data has utility for programs seeking to eliminate NTDs that remain a public health problem.

Reviewer #2: The authors have responded to the necessary revisions

Reviewer #3: The revision of PNTD-D-20-01771_R2 is improved from the previous version. I have no major concerns about the results in this manuscript.

Editorial and Data Presentation Modifications?
Use this section for editorial suggestions as well as relatively minor modifications of existing data that would enhance clarity. If the only modifications needed are minor and/or editorial, you may wish to recommend “Minor Revision” or “Accept”.

Reviewer #1: line 52: dracunculiasis should not be capitalized

Case changed for word.

line 451: insert period between (Fig 1 and 2). We demonstrated...

Period inserted on this line.
line 454: change "is" to "are" ...genome are not as...

Updated is to are.

Supplemental Fig. S1: the x-axis should read "Discriminant function 1" (because more than 1 PC was used in this discriminant function)

Thank you for this point. We have updated this the axis on this figure, in addition to improvements based on additional suggestions from reviewer 1.

Reviewer #2: Minor english corrections are needed and a careful read is suggested.
We have reread the paper for additional corrections and typos.

Reviewer #3: The authors have made many changes from the original MS and provided detailed responses to the points raised by the editor and reviewers. I recommend minor revisions aimed at providing further clarity in the writing especially to how the word “loci” is used.

As the editor suggested, replacing “loci” with “genes” if referring to the 3 targeted genes in the mtDNA genome would provide clarity and more accurately reflect that mtDNA arises from a single locus. Then the term “genotype” appears to be used correctly (lines 11-12). Otherwise, “multilocus” would seem more appropriate in that particular sentence, but this is in fact NOT correct since mtDNA arises from a single locus.

Changing “locus/loci” to “gene/genes” would affect many places in the MS including: lines 11-12, 137, 162, 327-346, 451. 455, and 487. Also, the caption for Fig 4.

In addition, it would be mean changing the terminology regarding “within loci” and “outside of loci” for lines 327-346, 470. An alternative possibility is to use “among targeted genes” and “among non-targeted” genes?

Also, the authors did use “gene” in lines 150-155. So seems a good reason to stay consistent.

Thank you for this point. We have updated instances of “loci” to “gene” in the manuscript and figures.

Other minor comments:

Lines 20-22. It may be more direct to refer to several genes or >1 gene rather than markers in this sentence.

We have updated the sentence to incorporate the full genome since variant may fall outside of genes.

Line 72: correct spelling of “phlyogenetic”

Spelling has been corrected.

Line 85-88: The way this revised sentence reads to me is that people are directly studying pathogen movement to indirectly study human movement. I would suggest that the reason genetic data is indirect is that we estimate pathogen population genetics to make inferences about human and pathogen movement. Or in other words, genetic data are used to indirectly study the movements of pathogens.

Thank for this suggestion. We have updated the sentence to “Genetic data can be used to indirectly study the movement of pathogens, bypassing the need for host movement data”.

Line 104: should this refer to mtDNA genes?

Thank you for this point. We have updated this sentence to be clear that the scope of our study does only cover additional mtDNA genes.

Line 126: replace with “were successfully sequenced”

Updated in the manuscript.

Line 129: consider replacing with “first reported in a previous assessment of genetic diversity of Chadian Guinea worms”.

Thank you for this suggestion. We have updated this sentence in the manuscript.

Line 259: Insert “of”

“Of” inserted into the sentence.

Line 447: Replace “on” with “in”

“On” replaced with “in” in this sentence.

Line 457: Add space before parentheses.

Space added.

Line 466: “Non-loci” is a misnomer?

Thank you for this point. Non-loci was used for untargeted regions of the genome. We have updated the manuscript to be specific about targeted and untargeted genes as suggested.

Figures and Tables:
1) Typo in Fig 1 – see comments in manuscript.

Additional letter removed from the legend.

2) hyphenate "blue-filled" in Supp Fig 1.

Hyphen added to the legend.
3) Delete "appear" in Supp Fig S6.

Word deleted.
4) Clarify what "current loci" refers to in Supp Fig S8.

Updated to clarify targeted genes in the study.

Summary and General Comments
Use this section to provide overall comments, discuss strengths/weaknesses of the study, novelty, significance, general execution and scholarship. You may also include additional comments for the author, including concerns about dual publication, research ethics, or publication ethics. If requesting major revision, please articulate the new experiments that are needed.

Reviewer #1: Eradication of many neglected tropical diseases is facing challenges in some regions. Key to successful elimination is identifying mechanisms that underlie continued transmission despite control measures. In this paper, genetic sequence data from Guinea worms are used to confirm dogs as a significant zoonotic reservoir, and to quantify the geographic scale of transmission. Developing genetic barcodes for use in surveillance should be considered (and debated) by the NTD community as a tool for program managers to clarify when movement of people or animal reservoirs drives continued transmission. The research presented here contributes to this discussion, and I recommend its publication.

Reviewer #2: The authors have responded to the necessary revisions but there are a few minor revisions as suggested.

Reviewer #3: This study demonstrates that barcoding and mitochondrial genome analysis can be used to better understand the distribution of the genetic diversity of dracunculiasis in Chad.

Additional comments from the returned PDFs:

Line 124/146: Add the geographical location for this isolate and host

Thank you for this important detail. The single publicly available *D. medinensis* reference genome does not have additional information regarding the host without an associated manuscript.

Line 130: So were these trimmed to remove these areas so they did not affect the analysis?

Variable alignment ranges with missing positions across sample and any positions in the gene without variants did not affect the analyses. The gene ranges reported cover the start and end regions by any sample.
